# Phytochemical mediated modulation of COX-3 and NFκB for the management and treatment of arthritis

**DOI:** 10.1038/s41598-023-37729-2

**Published:** 2023-08-21

**Authors:** Dipak Biswas, Bharat Gopalrao Somkuwar, Jagat Chandra Borah, Pritish Kumar Varadwaj, Saurabh Gupta, Zeeshan Ahmad Khan, Gopinath Mondal, Asamanja Chattoraj, Lokesh Deb

**Affiliations:** 1 Natural Product Chemistry and Pharmacology Programme, Medicinal Plants and Horticulture Resources Division, Institute of Bioresources and Sustainable Development, (An Autonomous Institute of Department of Biotechnology, Government of India), Takyelpat, Imphal, 795001 Manipur India; 2 Bioinformatics and Bioresources Database Division, Institute of Bioresources and Sustainable Development, (An Autonomous Institute of Department of Biotechnology, Government of India), Takyelpat, Imphal, 795001 Manipur India; 3 Institute of Advanced Study in Science and Technology, (An Autonomous Institute Under Department of Science & Technology, Govt. of India) Vigyan Path, Paschim Boragaon Garchuk, Guwahati, 781035 Assam India; 4 Department of Applied Sciences, Indian Institute of Information Technology, Devghat, Jhalwa, Allahabad, 211015 Uttar Pradesh India; 5 Biological Rhythm Laboratory, Animal Resources Programme, Institute of Bioresources and Sustainable Development, (An Autonomous Institute of Department of Biotechnology, Government of India), Takyelpat, Imphal, 795001 Manipur India; 6 Department of Animal Science, Kazi Nazrul University, Paschim Bardhaman, Asansol, 71330 West Bengal India; 7 Institute of Bioresources and Sustainable Development-Regional Centre, Sikkim (Department of Biotechnology, Government of India), 5th Mile, Near Metro Point, Tadong, Gangtok, 737102 Sikkim India

**Keywords:** Biotechnology, Computational biology and bioinformatics, Rheumatology

## Abstract

In this study, we investigated whether zerumbone (ZBN), ellagic acid (ELA) and quercetin (QCT), the plant-derived components, can modulate the role of COX-3 or cytokines liable in arthritic disorder. Initially, the effect of ZBN, ELA, and QCT on inflammatory process was investigated using in-vitro models. In-silico docking and molecular dynamics study of these molecules with respective targets also corroborate with in-vitro studies. Further, the in-vivo anti-arthritic potential of these molecules in Complete Freund’s adjuvant (CFA)-induced arthritic rats was confirmed. CFA increases in TNF-α and IL-1β levels in the arthritic control animals were significantly (***p < 0.001) attenuated in the ZBN- and ELA-treated animals. CFA-induced attenuation in IL-10 levels recovered under treatment. Moreover, ELA attenuated CFA-induced upregulation of COX-3 and ZBN downregulated CFA-triggered NFκB expression in arthritic animals. The bonding patterns of zerumbone in the catalytic sites of targets provide a useful hint in designing and developing suitable derivatives that can be used as a potential drug. To our best knowledge, the first time we are reporting the role of COX-3 in the treatment of arthritic disorders which could provide a novel therapeutic approach for the treatment of inflammatory disorders.

## Introduction

Cyclooxygenase-1 (COX-1) is invariably constitutive and maintains homeostasis in the gastric mucosa. It is also involved in cardiovascular diseases^1^ inhibition results in gastric damage^2^^,^ stroke^3^ and alters other vital functions. COX-1 is constitutive, while COX-2 is induced response and selective inhibition with a beneficial role^4^. COX-1 and 2 share structural similarities in their binding pockets,consequently, COX-2 inhibition also impedes COX-1 action^5^. Thus, the use of selective and non-selective COX inhibitors is declining. Cyclooxygenase-3 (COX-3), a splice variant of the enzyme COX-1, was first reported to be expressed in the canine brain and later in humans. It is expressed in a varied range of concentrations in the cerebral cortex, aorta, and other tissues of the body^6^. Both isoforms are derived from the same gene however, the COX-3 gene retains the intron-1 sequence at the mRNA level resulting in an addition of 30 amino acids at the N-terminal of the enzyme^7^. The role of COX-3 has been implicated in the inflammatory pathway whose inhibition is reported by NSAIDs-approved drugs such as acetaminophen potentiates the analgesic and antipyretic activities of the drug^8,9^. The paracetamol/acetaminophen shows slight inhibition of COX-1 or COX-2 however, owing to its lipophilicity it crosses the blood–brain barrier and induces effects such as analgesia and hypothermia in mice—actions attributed to COX-3 inhibition in the CNS and PGE2 down-regulation^10^. Several carboxylates containing NSAIDs such as diclofenac and ibuprofen are also potent COX-3 inhibitors,however, their action is limited to in-vitro cell cultures showing negligible concentrations in the brain owing to their high polarity and inability to cross the blood–brain barrier^7^. Thus, COX-3 due to its distinct role and expression pattern might pave the way for new target development in the treatment of inflammatory disorders. In present study, our one of the aim was to investigated the role of COX-3 in knee joint tissues of experimental arthritic animals.

Arthritis causes disability due to joint pain and stiffness. The pain, stiffness, swelling and redness in joints due to arthritis (both osteoarthritis and rheumatoid arthritis) are mainly caused by Prostaglandins (PGs), primarily PGE2 and prostacyclin, which are essential mediators of inflammation, pain, fever and can cause chondrocyte apoptosis^11,12^. These prostaglandins are synthesized in tissues, and its (mainly PGE2) precursor is arachidonic acid which is released from membranes by phospholipase A2. Arachidonic acid is metabolized by cyclooxygenase (COX) activity to form the prostaglandin endoperoxide H2. To date, three isoforms of COX (COX-1, COX-2 and COX-3) have been cloned. Among them, COX-3 is a newly described derivative form of COX-1^7^. However, owing to the lack of selective and potent inhibitors, there is little known about the role of COX-3 in arthritis management and analgesia.

Cytokines are small signaling proteins, usually less than 80 kDa in size, having a wide range of biological functions. The pro-inflammatory cytokines include IL-1β, IL-6, TNF-α, IL-9, IL-12, IL-18 etc., which orchestrate the immune response to an infecting organism and mount an inflammatory response. Anti-inflammatory cytokines including IL-4, IL-10, TGF-β etc. serve as feedback regulators of the immune response and prevent serious tissue damage by an overtly aggressive immune system^13^.

Several approach has been adopted for treatment of arthritic disorders. Classical treatment is for management of pain by using nonsteroidal anti-inflammatory (NSAIDs) drugs such as acetylsalicylic acid, naproxen, ibuprofen etc. and steroidal anti-inflammatory drugs like corticosteroids. But their adverse effects expressed as bone-thinning, obesity, hyper glycemia, immunosuppression. Secondly, diseases modifying anti-arthritic drugs (DMARDs) such as methotrexate, hydroxychloroqyine used clinically mainly to stop the progress of joint destruction and deformity in arthritic patients. But methotrexate caused hepatic disorders, bone marrow deterioration. However, in recent time monoclonal antibodies also known as biological DMARDs are used for treatment of arthritis. They are Tumor necrosis factor (TNF) inhibitors (etanercept, infliximab, golimumab etc.), Interleukin-1 (IL-1) inhibitor (Anakinra) and Interleukin (IL-6) inhibitor (Tocilizumab). But these biological DMARDs are very much expensive, they are mostly immune suppressants, contraindicated in patients of cardiac disorders and required continuous monitoring^14,15^.

However, various plant extracts have been known to stimulate or suppress the immune response through modulation of immune cell effector functions (cytokines, chemokines, etc.) such as Zerumbone from the *Zingiber zerumbet*^13^.

The *Zingiber zerumbet* is a tuberous plant and is found growing naturally near water bodies and shady areas in mountainous slopes or valleys. The rhizomes of the plant are reported as an anti-inflammatory, analgesic, antioxidant^16^^,^ anti-proliferative, antibacterial and antiapoptotic^17,18^. *Clerodendrum colebrookianum*, a perennial shrub, is mostly growing in the North-East regions as well as the western ghat area of India and is used by folklore healers for the treatment of hypertension, diabetes, blood purification, abdominal pain, diarrhoea, dysentery, cardiac, and cough^19,20^. Another perennial plant*, Averrhoa carambola* has also been recognized to have potential anti-inflammatory, analgesic, hypoglycemic, hypotensive and antioxidant properties. Phenotypically the plant is short (about 5–7 m), and multi-stemmed with a diameter of about 20–25 ft^21^. We extracted the phytoconstituents zerumbone (ZBN), ellagic acid (ELA) and quercetin (QCT) from the respective plant *Zingiber zerumbet*, *Clerodendrum colebrookianum,* and *Averrhoa carambola*. The isolated compounds were tested In-vitro and in-vivo in anti-arthritic models and found reasonably good agreement between virtual screening and experimental findings of bioactive compounds. In this study, investigated role of COX-3 in management of arthritis in specific and also explore other pro-inflammatory biomarkers such as IL-1, IL-6, IL-10, TNF- α and NFκB.

## Experimental procedure

### Plant materials

The rhizome of *Zingiber zerumbet* Roscoe (ZzR), (Voucher Specimen No: IBSD/M/1009), leaves of *Clerodendrum colebrookianum* Walp. (CcL), (Voucher Specimen No: IBSD/M/1014) and fruits of *Averrhoa carambola* Linn. (AcF), (Voucher Specimen No: IBSD/M/1010) were collected from the Imphal West district from December 2013 to January 2014. Identification was performed by Dr B. Thongam, Scientist-D, IBSD, Imphal, and voucher specimens of all three plants were deposited in the IBSD Herbarium.

### Extraction and isolation

The dried powder of ZzR was extracted in a Soxhlet extractor with ethanol (95%) and isolated ZBN as per the method described by Huang et al.^22^. The presence of ZBN was confirmed in the isolates by comparing them with commercial ZBN (Sigma) through high-performance liquid chromatography (HPLC), and further purification and identification were performed. Similarly, the ethyl acetate fraction was isolated from the dried powder of CcL as reported in our earlier publication^20^ and ELA isolated from the ethyl-acetated fraction of CcL was extracted by the method described by Lu et al.^23^. Furthermore, raw AcF (5 kg) was crushed into small pieces and extracted in a Soxhlet extractor with hydro-alcoholic solvent (50% ethanol). The extract was concentrated under reduced pressure. The QCT was isolated from the hydro-alcoholic extract of AcF as per the method described by Deore et al.^24^. The isolated products were confirmed as ZBN, ELA, and QCT, respectively, using analytical tools under a separate project (analytical data given in supplementary file, Figs. S31–S41).

### In-vitro anti-inflammatory study

#### Protease Inhibition

The protease inhibition assay was carried out as per the method described by Bijina et al.^25^. In brief, 1 mL of trypsin (0.5 mg/mL) prepared in 0.1 M phosphate buffer (pH7) was pre-incubated with 1 mL of different concentrations (100–400 µg/mL) of test compounds (ZBN, ELA, QCT, and DfS) at 37 °C for 15 min. After incubation, 2 mL of 1% casein prepared in 0.1 M phosphate was added, and the mixture was incubated at 37 °C for 30 min. The reaction was terminated by adding 2.5 mL of 0.44 M trichloroacetic acid, after which the solution was transferred to a centrifuge tube and centrifuged at 10,000 rpm for 15 min. The clear supernatant was collected, and absorbance/optical density (OD) was measured at 280 nm. The experiment was performed in triplicate. The efficacy of the test compounds was expressed in percentage inhibition. % Inhibition calculated by the formula—[(OD Control−OD Test)**/**OD of control × 100].

#### Heat-induced haemolysis

The heat-induced haemolysis assay was carried out as per our reported method^26^. In brief, 2 mL of reaction mixture consisting of 1 mL of test sample solution (ZBN, ELA, QCT or DfS) at different concentrations (10–100 µg/mL) and 1 mL of 10% RBC suspension was added to a 2 mL micro-centrifuge tube and incubated at 56 °C for 30 min in a water bath. The reaction mixture was cooled and centrifuged at 2500 rpm for 5 min. The supernatant was collected, and its absorbance was measured at 560 nm. Saline and DfS were used as the control and standard reference, respectively. The experiment was performed in triplicate. The efficacy of the test compounds was expressed in percentage inhibition. % Inhibition calculated by the formula—[(OD Control−OD Test)/OD of control × 100].

#### Inhibition of albumin denaturation

Assessment of the inhibition of albumin denaturation was carried out as per our reported method^26^. Reaction mixtures comprising 1% aqueous solution of bovine serum albumin (Sigma) and test compounds (ZBN, ELA, QCT or DfS) at different concentrations (10–100 µg/mL) were added to centrifuge tubes, and the pH was adjusted to 6.8 using 1N HCl. The solutions were incubated at 37 °C for 20 min, followed by heating at 57 °C for 20 min. The solution was cooled, and absorbance was measured at 660 nm using a double-beam spectrophotometer. The experiment was performed in triplicate. The efficacy of the test compounds was expressed in percentage inhibition. % Inhibition calculated by the formula—[(OD Control−OD Test)/OD of control × 100].

#### Selection of COX sequences

Nine and eleven Human cyclooxygenase sequences were taken from patents obtained by Qin et al.^6^ and Simmons et al.^27–29^, respectively. These sequences were used for evolutionary analyses that were performed in MEGA X. Sequence alignment, and phylogenetic tree revealed that cyclooxygenase homologs have a similar domain with > 60% sequence identity (Fig. S1 and Table S1). This sequence analysis also indicates that they share a common catalytic site and contains hydrophobic amino acids responsible for signaling^28^. In the absence of human COX1 and COX-3 crystal structures, we have selected a human crystal structure of cyclooxygenase isoform 2 for the modelling of COX-3^30^.

#### Target selection and molecular docking

Constructed protein model of COX-3 and crystal structures of human TNF-α (PDB ID 2AZ5) and IL-10 (PDB ID 1Y6K) were used for the initial screening of selected plant extract and known drugs (Table S1) using the GLIDE module of the Schrodinger software package^31,32^. A total of twenty-three ligands were prepared by the addition of hydrogen atoms, charge neutralization, and tautomer generation. All the torsional bonds of the ligand were set free, and a maximum of four stereoisomers were prepared for each ligand. Selected target proteins were prepared using the protein preparation wizard (Prep Wizard) in Maestro (Protein Preparation Wizard 2015-2,Epik version 2.4). The grids for each protein were prepared using GLIDE to identify the site on the target proteins where the ligands can interact during the docking process. Extra precision (XP) docking was performed to determine the optimal docked complexes^33^. The best docking complexes, based on the GLIDE docking score and conformation, were used for further Molecular dynamics simulations (MDS).

#### Execution of molecular dynamics simulations

MD simulations were performed for COX-3, TNF-α, IL-10 and COX-1 in complexes with ZBN, ELA, and QCT by using Desmond (Desmond Molecular Dynamics System, 2016). Each system was embedded in an orthorhombic box containing the SPC solvent model, and charges were neutralized using sodium and chloride ions as per the system requirements^34^. Initially, steric clashes of all systems were released by executing energy minimization. Systems equilibration was performed with NVT and NPT ensembles using the SHAKE algorithm, and the system temperature of up to 300 K and pressure of up to 1 bar was maintained. After equilibration, a 30-ns molecular dynamics simulation was executed for all complexes^33,35^. After completion of the simulation, the trajectory files of each complex were used to calculate the protein and ligand root-mean-square deviation (RMSD), the protein and ligand root-mean-square fluctuation (RMSF), the protein–ligand interaction and ligand torsion.

### In-vivo anti-arthritic study

#### Animals

Male albino Sprague–Dawley rats weighing 200–250 g were procured from the Regional Institute of Medical Sciences (RIMS), Imphal. The animals were fed ad libitum, housed in polypropylene cages, and maintained at 25 ± 2 °C and a 12-h dark/light cycle. Animals were acclimatized to the housing conditions for one week. The bedding in the cages was renewed daily to ensure hygienic conditions and maximum comfort for the animals. All experimental procedures were conducted in accordance with guidelines set by the Committee for the Purpose of Control and Supervision of Experiments on Animals (CPCSEA), Government of India. Ethical clearance for animal handling and experimentation was obtained from the Institutional Animals Ethical Committee (IAEC), IBSD, Imphal (approval No.- IBSD/IAEC/FS/ICMR/21), before initiation of the experiments and reported accordance with ARRIVE guidelines. Animals were distributed in six different groups containing five in each as per the approval of IAEC. Normal healthy control (NC), CFA-induced arthritic control (AC), Zerumbone (ZBN) treated, Ellagic acid (ELA) treated, Quercetin (QCT) treated and standard diclofenac sodium (DfS) treated animal groups were taken for this experiment.

#### Induction of arthritis

Joint inflammation was induced in the rats via intra-articular injection of 0.1 mL Complete Freund’s adjuvant (CFA) (Sigma-Aldrich, F5881) into the right knee joint of rats. CFA each mL contains 1 mg Mycobacterium tuberculosis (H 37RA, ATCC 25177), heat-killed and dried, 0.85 mL paraffin oil and 0.15 mL mannide monooleate (Sigma-Aldrich, F5881). As a control, some animals were injected with 0.1 mL of normal saline^36^. The circumference of the hind paw to the knee joint was measured using a Plethysmometer (Model 7141, UGO Basile, USA) on days 0, 1, 7 and 14 after injection. Pain intensity at the knee joint of the animals was recorded using a Pressure Application Measurement (PAM) device (UGO Basile, USA) on days 0, 7 and 14 after injection as per the instruction by UGO Basile, USA for use of the machine.

#### Treatments

ZBN (50 mg/kg/day), ELA (50 mg/kg/day), QCT (50 mg/kg/day), Standard diclofenac sodium (DfS) at 50 mg/kg/day dose or pure mineralized drinking water (5 mL/kg/day; control group) was administered orally (P.O.) by using purified water as vehicle. To study the effect of the test compounds and standard drugs on secondary inflammatory reactions in arthritic animals, all chemicals and controls were administered for 7 days after the initial 7 days of CFA injection (i.e., beginning on the 8th day and ending on the 15th day of the experiment). The circumference of the hind paw was measured using a Plethysmometer (Model 7141, UGO Basile, USA) on days 0, 1, 7 and 14 after injection. Pain at the knee joint of the animals was recorded during the study using a PAM device (UGO Basile, USA) that expressed as a force in gf. On the 15th day, animals were sacrificed, and blood and knee joint tissue were collected for ELISA and western blotting analysis. The doses of ZBN, ELA and QCT, were selected as reported in published literature and a similar dose of DfS was taken to compare the potential^37–39^. Pain at the knee joint of the animals was recorded during the study^40^ using a PAM device (UGO Basile, USA) that expressed as a force in gf. On day 1 pain intensity and paw edema were recorded 1 h after administration of the drugs with an aim to investigate the acute effect of drugs as pure compounds expected to have rapid bioavailability.

#### Estimation of the levels of TNF-α, IL-10, and IL-1β in serum and knee joint tissue

The concentrations of TNF-α, IL-10, and IL-1β in serum and knee joint tissue extract were determined with ELISA kits. On the 15th day after the CFA injection, animals were anesthetized with sodium pentobarbital (50 mg/kg, IP). Blood was collected and stored at 4 °C for 30 min and then centrifuged at 15,000 rpm for 10 min in a refrigerated centrifuge (Eppendorf-5430R). Subsequently, knee joint tissues, including bone, were collected in 10% PBS, homogenized, and centrifuged at 15,000 rpm for 10 min. The supernatants were collected, treated with a protease inhibitor cocktail (Sigma) and stored at – 20 °C until further Use. The levels of TNF-α, IL-10 and IL-1β, were determined using commercially available enzyme immunoassay kits (Invitrogen, USA). The measurement was completed using an ELISA reader at 450 nm (SpectraMax® MM5e Plus, Molecular Devices, USA).

#### Assessment of COX-3 and NFκB expression by western blotting

Rat knee joints were removed and stored in RIPA buffer containing protease inhibitor (Promega, USA) at − 80 °C until further use. The samples were thawed on ice, sonicated with 10–15 short bursts in a sonicator (Vibra-Cell, SONICS, USA) and centrifuged at 17,000 rpm for 20 min in a micro-centrifuge at 4 °C. The resulting supernatant was carefully collected in a 1.5 mL micro-centrifuge tube, and the protein concentration was estimated (RC DC protein assay, Bio-Rad Laboratories). A total of 60 µg of protein per sample was mixed with 2 × SDS sample, boiled at 95 °C and then cooled on ice for 5 min before separation via SDS-PAGE on a 12% gel and subsequent transfer onto a 0.45 μm PVDF (Immobilon, Millipore, India) membrane. Membrane blocking was performed at 4 °C in blocking solution (5% non-fat milk in TBST buffer: 50 mM Tris, 100 mM NaCl, 0.1% Tween 20, pH 7.4) overnight^41,42^. The membranes were then washed with TBST buffer and incubated overnight in a blocking solution containing primary antibodies targeting COX-3 (Santa Cruz, 1:1000 dilution), NFκB (p65) (Santa Cruz, 1:1000 dilution) or β-actin (Ambion-AM4302, 1:5000 dilution, control). The membranes were then washed with TBST buffer and incubated for 1 h at room temperature with the alkaline phosphatase-conjugated secondary antibody (Sigma, 1:5000 dilutions, goat anti-mouse antibody for COX-3 and goat anti-rabbit for NFκB (p65) detection). Finally, the membranes were washed 5 times for 5 min each with TBST. The membranes were developed with BCIP/NBT (GeNeiTM). The intensity of the individual band of each immunoblot was quantified by densitometry using ImageJ software^43^.

### Statistical analysis

Average values of the raw data were expressed as the mean ± SEM, n = 5. For numerical results, one-way analysis of variance (ANOVA) with Tukey–Kramer Multiple Comparisons post-tests was performed using GraphPad InStat Version 3 (GraphPad Software). The minimum value of p < 0.05 was considered significant. *^C^p < 0.05, **^C^p < 0.01, and ***^C^p < 0.001 indicate significant differences in CFA-induced arthritic control group comparisons to the healthy control group; *p < 0.05, **p < 0.01, and ***p < 0.001 indicate significant differences of test groups comparisons to the CFA-induced arthritic control group.

Simulation scatter line plots were plotted using R version 3.4.2, and the R-code used is appended as supplementary files (RS-1, RS-2, RS-3, RS-4)^44^.

## Results and discussion

### In vitro testing of plant extract in anti-inflammatory assays

**Figure 1:**
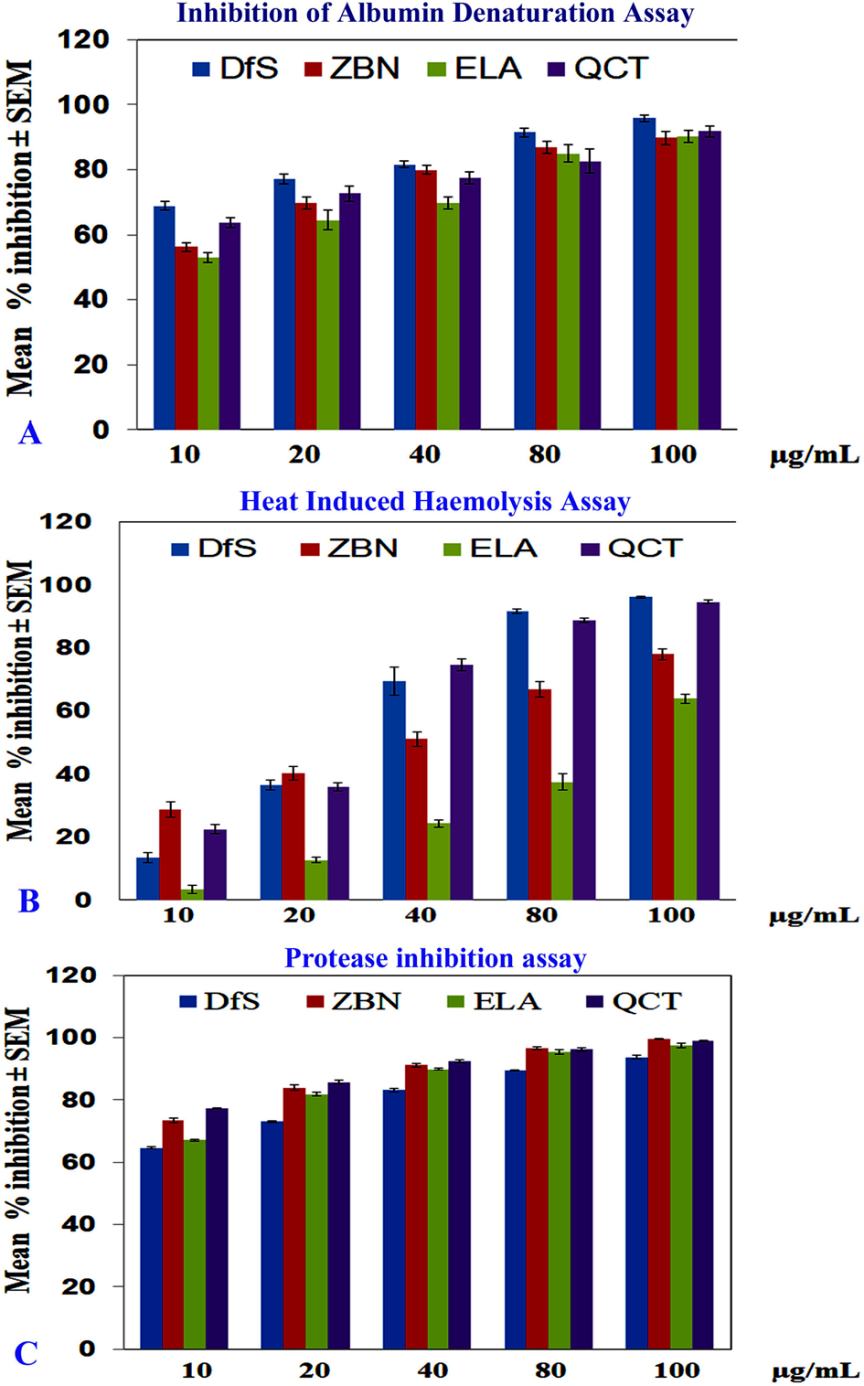
Effect of zerumbone (ZBN), ellagic acid (ELA), quercetin (QCT) and standard diclofenac sodium (DfS) on in-vitro anti-inflammatory assays. (**A**) Inhibition of albumin denaturation, (**B**) heat-induced hemolysis, (**C**) protease inhibition assay. The average values of the raw data are expressed as the mean ± standard error mean (SEM), n = 3.

In the albumin denaturation assay, external stress was applied using concentrated acid or base, inorganic salt on organic solvents, or heat. Consequently, bioactive proteins lost their function due to the breakdown of their secondary and tertiary structure, and protein denaturation has been identified as a cause of inflammatory reactions^26^. Hence, anti-inflammatory agents should have an inhibitory effect on heat-induced protein denaturation. ZBN, ELA, QCT, and DfS inhibited heat-induced albumin denaturation in a dose-dependent manner (Fig. 1A). A heat-induced hemolysis assay was carried out to determine the stability of the membrane of human red blood cells under stress. The erythrocyte membrane is akin to the lysosomal membrane, and the stability of the lysosomal membrane determines the extent of inflammatory responses by preventing the release of activated neutrophils and other enzymes, which causes several disorders due to pro-inflammatory conditions. Hence, anti-inflammatory agents should have an inhibitory effect on heat-induced hemolysis^26^. ZBN, ELA, QCT, and DfS indicated inhibitory effects on heat-induced hemolysis in a dose-dependent manner (Fig. 1B). Proteases have vital roles under arthritic conditions. The lysosomal granules of neutrophils carry numerous serine proteases. Leukocyte-derived proteases have been reported to contribute to tissue damage during inflammatory reactions, and protease inhibitors have been reported to significantly protect against this effect^25^. ZBN, ELA, QCT, and DfS also showed protease inhibition in a dose-dependent manner (Fig. 1C). These observations encouraged to study of the anti-arthritic effects of the test compounds and their mechanism of action in vivo CFA-induced arthritic animal models to further confirm the efficacy of the products.

### In-vitro observation confirmed by molecular docking and dynamics simulations studies

**Figure 2:**
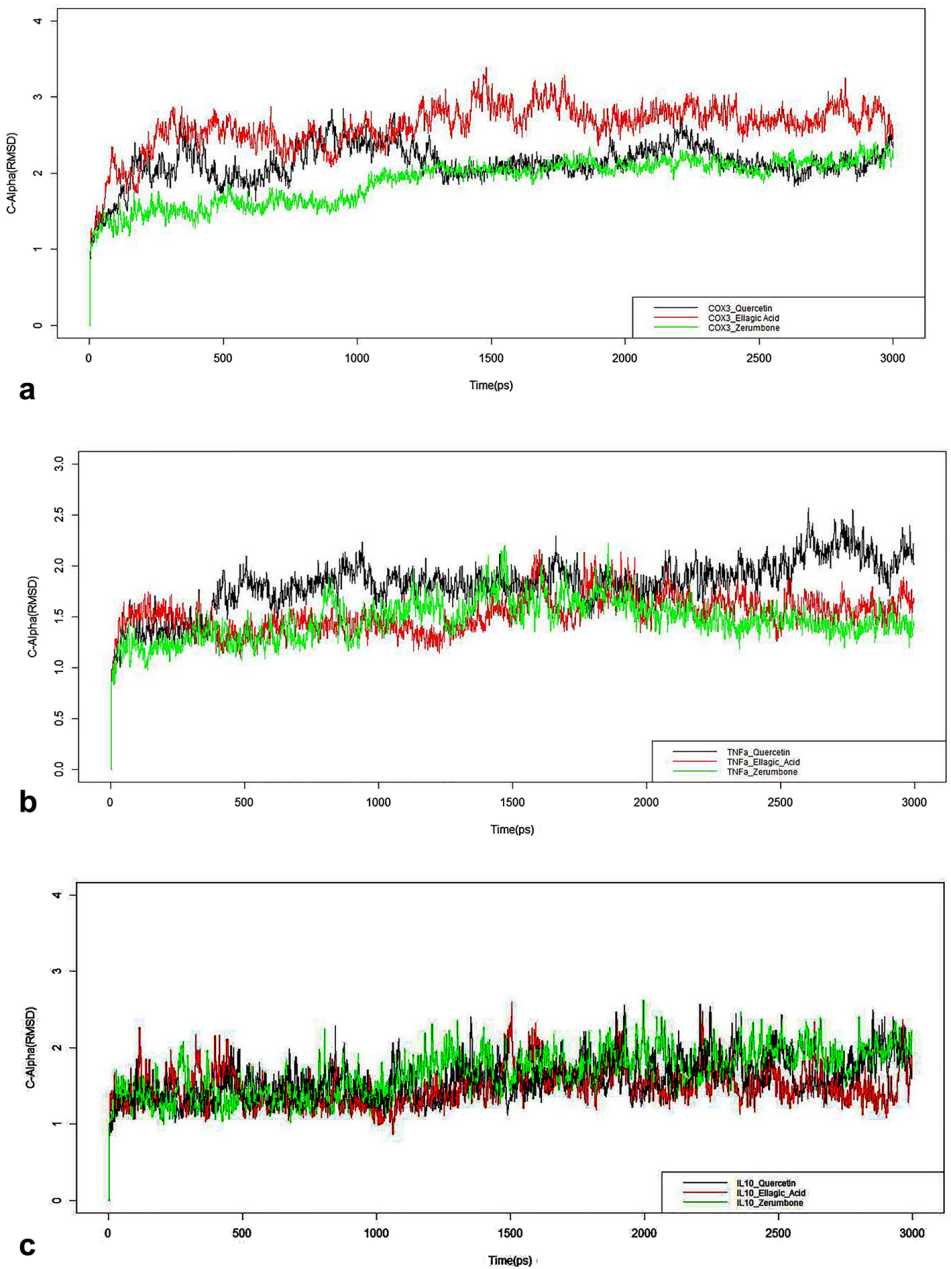
The structural stability of the protein–ligand complex monitored by the molecular dynamic simulations (30-ns). (**a**) COX-3 model bound to quercetin, ellagic acid, and zerumbone. Ellagic acid exhibits a greater RMSD value as the time progresses, while quercetin and zerumbone exhibit an almost constant stability pattern after 12 ns, except at approximately 22 ns. (**b**) TNF-α bound to quercetin, ellagic acid, and zerumbone. Quercetin exhibits a greater RMSD value as time progresses, while ellagic acid and zerumbone exhibit an almost constant stability pattern after 12 ns. (**c**) Interleukin (IL-10) bound to quercetin, ellagic acid, and zerumbone. Quercetin, ellagic acid and zerumbone exhibit almost constant RMSD values as time progresses. All the compounds (except ellagic acid) showed optimal stability at 27–30 ns.

**Figure 3:**
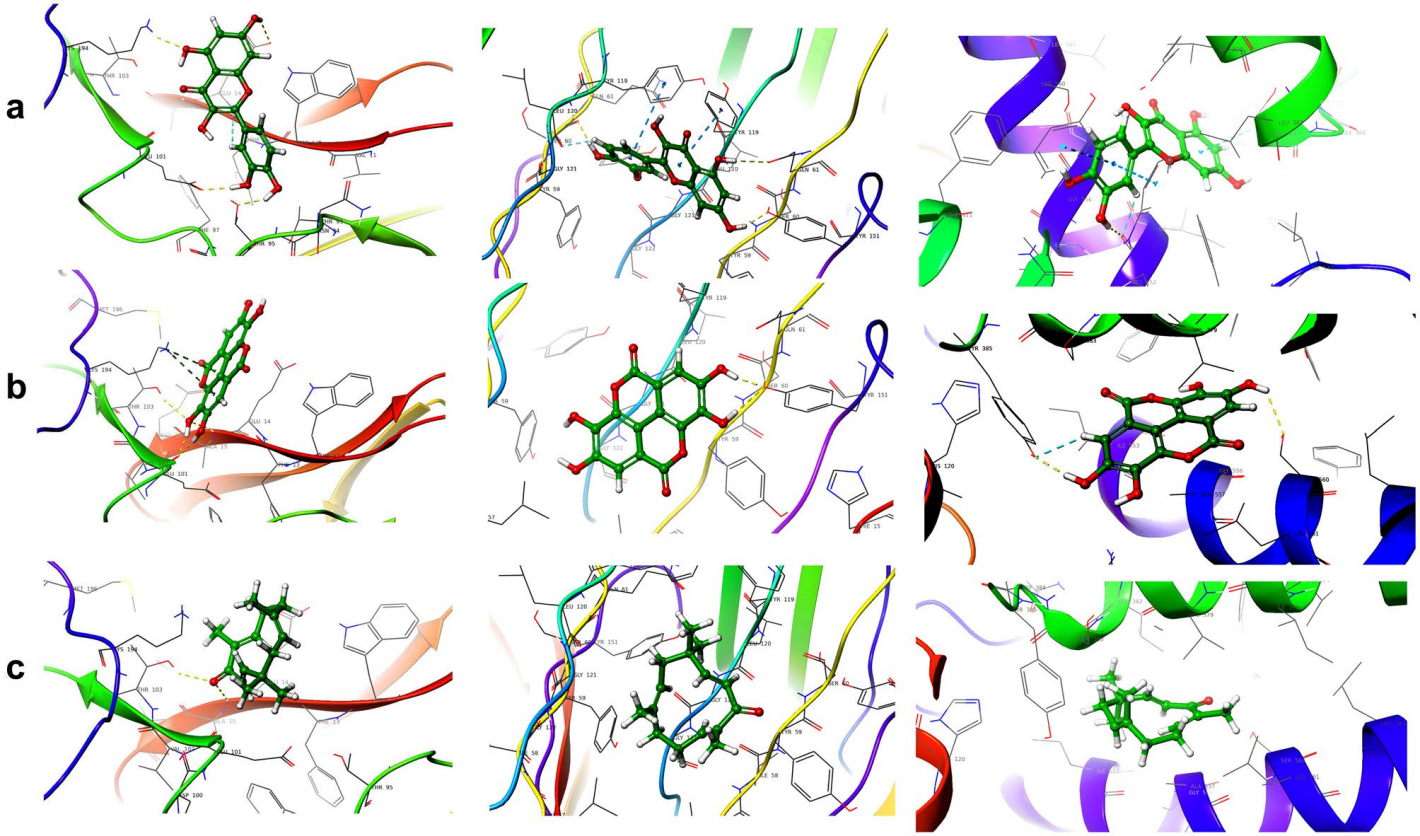
The 3D target-ligand hydrogen bond interaction of IL-10 with (**a**) quercetin (**b**) ellagic acid and (**c**) zerumbone; TNF-α with (**a**) quercetin, (**b**) ellagic acid and (**c**) zerumbone; and COX-3 with (**a**) quercetin, (**b**) ellagic acid and (**c**) zerumbone.

Docking of selective and non-selective ligands (including natural bioactive compounds) was performed to estimate the specificity and affinity of drug–receptor interactions^45^. The amino acids present in and surrounding the active site of the binding domain of the receptor play a critical role in these interactions (Supplementary Table S5). Various residues in cyclooxygenases (COX) exhibit important functions such as acetylation (TYR-385), covalent modifications and hydrophobic pocket formation (SER-560), intermolecular interactions and salt bridge formation (ARG-120), and other functions, including heme binding, hydrogen bond abstraction, stearic hindrance, preferential presentation of active site residues that favour binding with ligands and proper folding^45–47^. The docking results of twenty-three selective and non-selective ligands, including natural isolates (QCT, ELA, ZBN), with COX-3, TNF-α, and IL-10 shown in Figs. 2 and 3 and their significant docking scores depicted in supplementary Fig. S2. The 2D interaction also confirms that the docked complexes have enough evidence for ligand affinity and specificity (Supplementary Fig. S3). Along with MDS were monitored the best ligand-receptor complex stability at specific time intervals. The molecular trajectory file shows that the variation in stability in vitro is a more appropriate method to simulate target-ligand complexes as per in-vivo conditions (Fig. 2).

The simulation was executed for QCT, ELA, and ZBN for all three receptors (IL-10, TNF-α, and COX-3). ELA exhibits a greater RMSD value as time progresses, while QCT and ZBN represent an almost constant stability pattern after 12 ns, except at approximately 22 ns. QCT exhibits a greater RMSD value as time progresses, while ELA and ZBN exhibit an almost constant stability pattern after 12 ns. For IL-10, QCT, ELA, and ZBN exhibit almost constant RMSD values as time progresses; however, ELA showed the best stability at 27–30 ns. The RMSF (A^0^) and the B factor values of C alpha carbons of amino acids on COX-3, TNF-α, and IL-10 interacting with (a) QCT (b) ELA and (c) ZBN elucidate the binding affinity (Figs. S11, S16 and S21). Notably, IL-10 forms hydrogen bonds and π-cation interactions. Four hydrogen bonds formed with QCT (GLU-14, THR-95, GLU-101 and LYS-194); four hydrogen bonds (ALA-15, GLU-101- two, THR-103) and two π-cation bonds (LYS-194 two) formed with ELA; and two hydrogen bonds formed with ZBN (ALA-15, THR-103) (Supplementary Figs. S11, S17–S20). TNF-α shows hydrogen bonds and π–π stacking interactions. Specifically, four hydrogen bonds (GLN-61, SER-60-B chain, TYR-151, and TYR-151-B-chain) and two π–π stacking interactions (TYR-119-A-chain, TYR-119-B-chain) formed with QCT; two hydrogen bonds (TYR-151) formed with ELA; and no interactions were observed with ZBN (Supplementary Figs. S5, S12–S15). COX-3 exhibits hydrogen bond and π–π stacking interactions. One hydrogen bond (MET-552) and three π–π stacking interactions (TYR-385, TRP-417, TYR-415) formed with QCT; two hydrogen bonds (TYR-385, SER-560) formed with ELA; and no interaction was observed with ZBN (Supplementary Figs. S4, S7–S10). Because COX-3 is considered an isoform of COX-1, assessments of the hydrogen bonds and other interactions of the compounds with COX-1 were also performed to validate the findings. COX-1 forms three hydrogen bonds with QCT (ARG-119, TYR-354, SER-529) but no interaction was observed with ELA and ZBN at all (Fig. 3).

**Figure 4:**
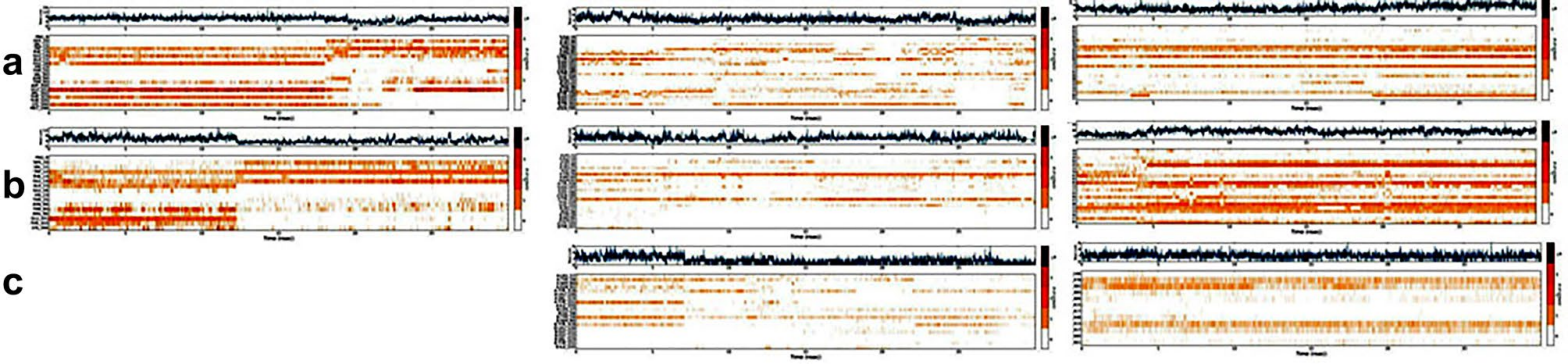
Partial least squares simulation graph at 30 ns with amino acids of IL-10 interacting with (**a**) quercetin (**b**) ellagic acid and (**c**) zerumbone; of TNF-α with (**a**) quercetin, (**b**) ellagic acid and (**c**) zerumbone; and of COX-3 with (**a**) quercetin, (**b**) ellagic acid and (**c**) zerumbone. The scale on the right side of the graph indicates no contacts between IL-10 and ellagic acid, which indicates morePLS simulation, while the lack of contact points between IL-10 and zerumbone may be due to no hydrogen bond interactions.

The residue number has a similar function as mentioned in Supplementary Table S5. From an evolutionary perspective, COX-3 was found to be an isolate of the cyclooxygenase and it is an isoform of COX-1 and has similar physicochemical properties (Table S1); hence, we also conducted MD and MDS of COX-1 with QCT, ELA, and ZBN to validate the specificity and affinity. ELA exhibits a greater RMSD value as time progresses, while QCT and ZBN exhibit an almost constant stability pattern after 12 ns. The only QCT achieves a stable trajectory after 27–30 ns (Supplementary Fig. S22). From the observations, we found that only QCT forms 3 hydrogen bonds with COX-1, while neither ELA nor ZBN bond interactions were observed (Table S4, Supplementary Figs. S23–S26). Furthermore, the residual interaction fraction and amino acid histogram for COX-1 with (a) QCT, (b) ELA and (c) ZBN showed specific residues involved in the active site of the receptor-ligand interaction (Supplementary Fig. S28). The RMSF (A^0^) and the B factor values of C alpha carbons of amino acids on COX-1 interacting with (a) QCT (b) ELA and (c) ZBN elucidate the binding affinity (Supplementary Fig. S29). The affinity and specificity observations were thus corroborated by partial least squares (PLS) graphs, which represent the number of contacts between the ligand and receptors (Supplementary Fig. S27). Similarly, PLS graphs throughout the simulations for COX-3, TNF-α, and IL-10 with QCT, ELA and ZBN were generated (Fig. 4) which indicates interactions of these ligands with catalytic domain residues of the target protein.

Based on the above observations, ZBN showed less affinity and specificity for TNF-α and COX-3. The 2D structures of QCT, ELA, and ZBN are shown in Supplementary Fig. S30 (a, b and c, respectively), and ZBN does not disrupt any drug-like properties as calculated from the open source DruLiTo, Marvin Suite 18.14 and Drug Bank (Table S2). These results were further used to perform in vivo and in-vitro studies. Experimental evidence from cell inhibition and bioassays has confirmed the presence of new cyclooxygenases in canines, rats, mice, and humans^6,7,27–29^. MD and MDS highlight the specificity and affinity of ligand-receptor interactions by calculating the docking score. The type, number of bonds, nature, and size of the amino acids at the binding site are some of the important parameters that often anticipate ineffective bond interactions. Comparative analysis of bond interactions (Supplementary Table S5) and observed cyclooxygenase-specific bond interactions (Table S4) shows the involvement of amino acid residues in the active site during docking. The docking score confirms the binding of ZBN with IL-10 (two hydrogen bonds) but not with TNF-α or COX-3, QCT binds with IL-10 (four hydrogen bonds), TNF-α (four hydrogens and two π–π stacking bonds) and COX-3 (one hydrogen and three π–π stacking bonds), and ELA can interact with IL-10 (four hydrogen bonds and two π–cation bonds), TNF-α (a hydrogen bond doublet) and COX-3 (two hydrogen bonds). The π–π stacking and π–cation bonds observed with the aromatic ring of the parent molecule essentially provide vital clues that the parent ring structure should be maintained while designing new molecules. Some of these residues are hydrophobic in nature and favour the formation of hydrogen bonds. The small and large sizes of amino acids surrounding at the active site are also accountable for binding specificity and affinity. Ligand entry into the binding pocket will be affected by small and/or large amino acids present at the point of entry in the active site. The inhibitory potential can be determined from the degree of interactions. Comparing the annotations from Table 1 and Tables S3 and S4, we found that

**QCT: TNF-α > IL-10 > COX-3 > COX-1,**

**ELA: IL-10 > COX-3 > TNF-α > COX-1,**

**ZBN: IL-10**.

### In-vivo CFA-induced arthritic model

**Figure 5:**
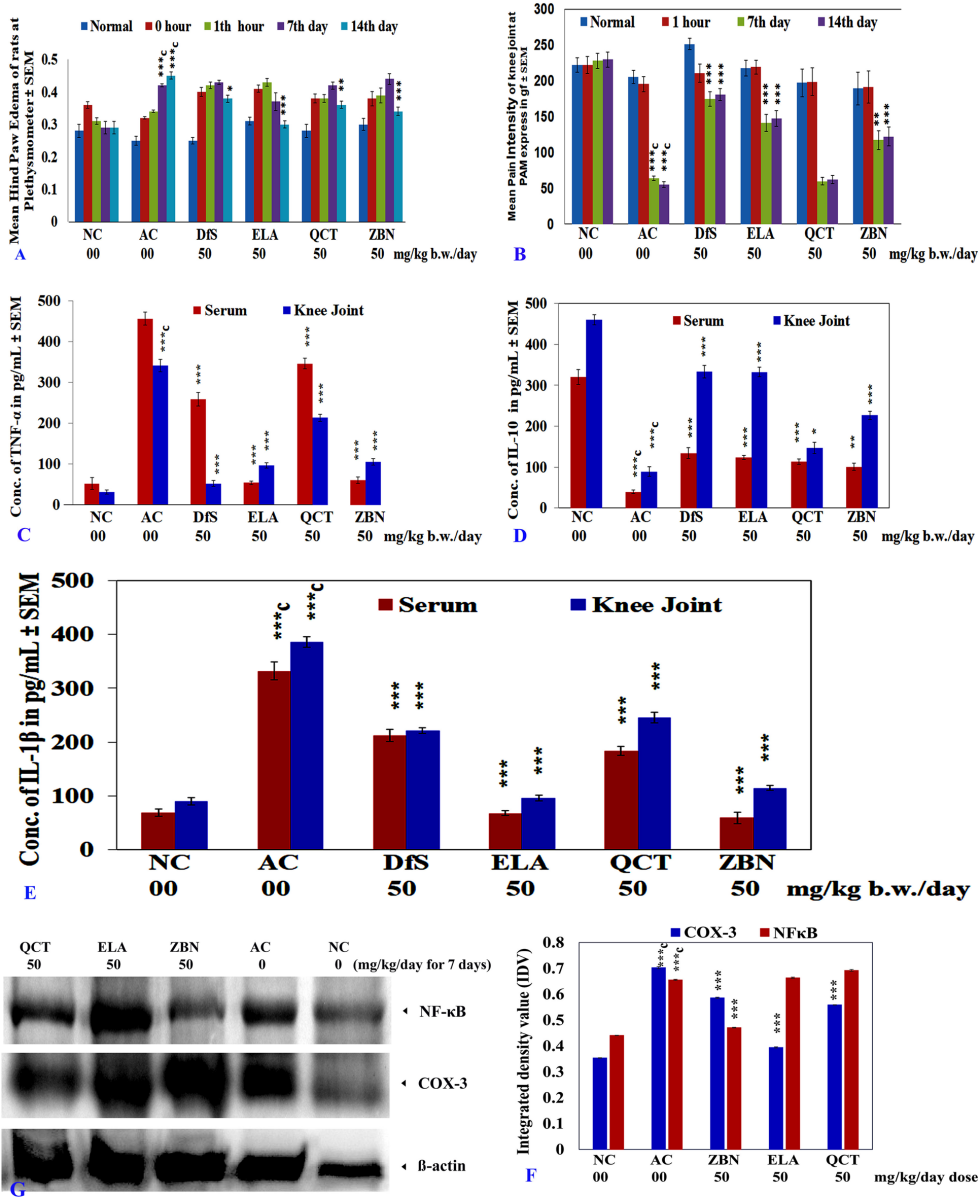
Effect of zerumbone (ZBN), ellagic acid (ELA), quercetin (QCT) and standard diclofenac sodium (DfS) on *in-vivo* anti-arthritic bioassay. (**A**) Hind paw edema of healthy (NC) and CFA-induced arthritic (AC) control rats. (**B**) Pain intensity in the knee joint of healthy (NC) and CFA-induced arthritic (AC) rats. (**C**) Levels of TNF-α insera and knee joint tissues from and AC rats. (**D**) Levels of IL-10 insera and knee joint tissues from NC and AC rats. (**E**) Levels of IL-1β insera and knee joint tissues from NC and AC rats. [Average values of the raw data were expressed as the mean ± SEM, n = 5. For numerical results, one-way analysis of variance (ANOVA) with Tukey–Kramer Multiple Comparisons post-tests was performed using GraphPad InStat Version 3 (GraphPad Software). The minimum value of p < 0.05 was considered significant. *^C^p < 0.05, **^C^p < 0.01, and ***^C^p < 0.001 indicate significant differences of CFA-induced arthritic control group comparisons to the healthy control group; *p < 0.05, **p < 0.01, and ***p < 0.001 indicate significant differences of test groups comparisons to the CFA-induced arthritic control group] (**F**) CFA-induced increases in COX-3 and NFκB expression in knee joint tissues from AC rats. A group of NC rats was used as a reference control (western blotting analysis). [Average values of the raw data were expressed as the mean ± SEM, n = 3] (**G**). Zerumbone (ZBN) inhibited CFA-induced overexpression of COX-3 and NFκB. However, ellagic acid (ELA) and quercetin (QCT) inhibited CFA-induced overexpression of COX-3 but not CFA-induced overexpression of NFκB expression. CFA-induced arthritic rats were treated with test compounds at a dose of 50 mg/kg/day for 7 days. Knee joint tissue extracts were prepared in RIPA buffer containing aprotease inhibitor and subjected to western blotting.

CFA-induced arthritis in rats is a commonly used sub-chronic or chronic inflammation model to study the pathophysiological and pharmacological events of inflammatory reactions. It is also used to evaluate the analgesic efficacy and/or anti-inflammatory potential of drugs. In CFA-induced arthritic rats shown joint/hind paw swelling, synovial-membrane inflammatory features and cartilage damage, all those akin to the clinical condition of arthritic patients^36^. In the current study, the hind paw of CFA-injected animals remained more swollen at 14 days than the paws of healthy animals. The paw edema of arthritic control animals was significantly increased at the 7th day post-CFA injection onwards compared to normal animals. However, paw edema of DfS, ELA, QCT and ZBN-treated arthritic animals was significantly (*p < 0.05, **p < 0.01, and ***p < 0.001) reduced after 14 days of treatment compared to that of the arthritic control animals (Fig. 5A). On prolonged used of the same dose of the drugs, DfS, ELA, QCT and ZBN may produce chronic therapeutic effect and edema reduced only after day 14 days of treatment. Moreover, after 1 h of the administration of test drugs, edema was measure (1 h), but edema was not reduced significantly, because may be given dose was not enough to produce acute effect even till day 7 of the treatment on CFA induced severe inflammatory condition. The pain intensity of the animals was measured using a PAM apparatus, where the animals showed a quick reflex of the knee joint in the case of more pain when the pressure was applied, the data are expressed as a force in gf. The pain intensity of the arthritic control animals was significantly increased on the 7th day post-CFA injection onwards compared to that of the healthy normal control animals. However, the pain intensity of the knee joint of arthritic animals treated with DfS, ELA and ZBN was significantly (**p < 0.01, and ***p < 0.001) reduced after 7 days of treatment compared to that in the arthritic control animals. On used of DfS, ELA and ZBN may inhibit prostaglandin release effectively due to the inhibition of COX enzymes^36^. But in QCT-treated arthritic animals, the pain intensity was not significantly reduced (Fig. 5B). The pro-inflammatory cytokines TNF-α and IL-1β have been reported to play a vital role in the pathogenesis of arthritis in animals and humans. It has been shown that TNF-α and IL-1β are expressed in the knee joint and the serum of arthritic patients^36^. TNF-α and IL-1β elevate the proliferation of fibroblasts, activates prostaglandin (PGE_2_) production, and increase the expression of other relevant cytokines. Consequently, collagen synthesized by synovial cells causes damage to cartilage and bone^36^. TNF-α has an essential role as a “master regulator” of inflammatory cytokine production in many inflammatory diseases,therefore, TNF-α is known as a therapeutic target for many inflammatory diseases^48^. In this study, a marked increase in the levels of TNF-α and IL-1β was observed in the serum and knee joint tissue extract of arthritic control animals on the 15th day after CFA treatment. However, treatment with ZBN, ELA, QCT or DfS had a marked inhibitory effect on the CFA-induced expression of TNF-α (Fig. 5C) and IL-1β (Fig. 5E). The anti-inflammatory cytokine IL-10 has been reported to play a vital role in resolving chronic inflammatory processes by inhibiting the production of pro-inflammatory cytokines, including IL-1β and TNF-α. It has been reported that the arthritic phenotypes in animal models (AMs) and arthritis symptoms in rheumatoid arthritis (RA) patients were ameliorated upon IL-10 and recombinant human IL-10 administration, respectively^36^. In the present study, CFA treatment was observed to decrease the serum IL-10 concentration compared to that in healthy control rats. However, treatment with ZBN, ELA, QCT or DfS markedly (*p < 0.05, **p < 0.01, and ***p < 0.001) reversed the CFA-induced reduction in IL-10 expression (Fig. 5D).

Higher levels of IL-1β and IL-10 were estimated in the knee joint extracts than in the sera of animals; however, the level of TNF-α was estimated to be greater in serum than in knee joint extract. The cytokine IL-10 has potent anti-inflammatory properties and is critical in preventing the host’s immune response to pathogens, thereby preventing damage to the host, and maintaining normal tissue homeostasis^49^. Nuclear factor-kappa B (NFκB) resides in the cytoplasm of unstimulated cells in all latent forms and, upon stimulation, translocate to the nucleus to execute its functions. Activation of NFκB ultimately triggers the release of pro-inflammatory cytokines such as IL-1β, IL-6, and TNF-α. Consequently, IL-1β, TNF-α, and IL-6, have been identified as safe and potential targets for the treatment of many auto-inflammatory diseases, including arthritis^50^. NFκB is found in human synovial tissue during the early stages of joint inflammation and remains at the late stages of inflammation. NFκB activation has been reported in RA patients and different AMs for RA, including adjuvant arthritis in rats^51^.

CFA Notably upregulated NFκB expression in the knee joint of arthritic animals compared to that in healthy control animals. ZBN treatment markedly downregulated CFA-induced overexpression of NFκB in rats; however, ELA and QCT treatment did not show an inhibitory effect on CFA-induced NFκB overexpression in rats (Fig. 5F and G) Several hypotheses have been proposed for the role of COX-3 in chronic inflammatory diseases, including RA^45^. In the present study, CFA markedly upregulated COX-3 expression in knee joint tissue of arthritic animals compared to healthy control animals, which was a novel finding for COX-3. ELA treatment markedly inhibited CFA-induced upregulated expression of COX-3 in knee joint tissue extracts of arthritic rats. However, QCT and ZBN treatment showed comparatively fewer inhibitory effects on the CFA-induced overexpression of COX-3 in rats (Fig. 5F and G). Tissues from the DfS-treated rats were not included in western blotting due to accidental damage to the samples during processing,however, DfS has been reported to be the most potent COX-3 and NFκB inhibitor^7^. This is the first report on the role of COX-3 in arthritic pathophysiological conditions and arthritis treatment. ELA has an inhibitory effect on CFA-induced overexpression of COX-3 but does not have an inhibitory effect on CFA-induced overexpression of NFκB, indicating that two different pathways may be involved, and two different drugs might exert varying effects under similar pathophysiological conditions. COX-3 inhibition-targeted treatment may provide novel therapeutic tools for chronic inflammatory disorders, including RA.

From the bond interaction table (Table S1) and specific bond interactions (Tables S3, S4), it is clear that ZBN forming interactions with IL-10; however, TNF-α, COX-3, and COX-1 do not show any affinity towards this molecule. Furthermore, ZBN has been shown to up and downregulate IL-10, TNF-α, and COX-3 in AMs. The ZBN has antimicrobial, antipyretic, antispasmodic, anticonvulsant, antiulcer, antioxidant, antidiabetic, antitumor, anticancer, anti-inflammatory, analgesic, antiallergenic, antiangiogenic, antiadipogenic, anticoagulant, and hepatoprotective properties^52^. The literature shows that ZBN is an active constituent in the treatment of various disease conditions. As an anticancer ZBN targets β-catenin in the cytoplasm as well as nucleus bound to the Lymphoid Enhancing Factor 1(LEF1)/Transcription Cell Factor (TCF-4). The Physicochemical properties of ZBN revealed that it is a sesquiterpene with a rigid ring system, no rotatable bonds and three chiral carbon sites. The lone pair of electrons (essential α, β—carbonyl atom) that forms Michael adducts (irreversible covalent bond by nucleophilic substitution) with a target to produce desired effects^53,54^.

Being the master regulator of apoptosis, inflammation, and immune response Nuclear Factor κB (NFκB) plays a vital role in the modulation of cell cycle response. The complex NFκB-subunit p50/p65 interaction with ZBN is unsatisfactory due to the presence of ZBN away from the binding site^53,55–58^. Also, Murakami et al.^59^ showed the formation of Michael adducts formation with ZBN and not with Humulene. The presence of α, β—carbonyl atom and Chiral Carbon at (C2–C3, C6–C7 and C10–C11) bend despite inherent rigidity in ZBN provides further exploration on the exact pathway and/or mechanism anticipate in ZBN as an important ligand used for treatment in various disease conditions. Hence, the profound quantitative structure–activity relationship, quantitative structure–property relationship, and ADMET investigations have a wide scope in the design and development of appropriate salts or drug derivatives that actively exhibit affinity and specificity towards IL-10, TNF-α, and COX-3. The membrane-bound COX-3 is expressed in the brain, heart, skeletal muscles, liver, stomach, and small intestine^28,29^. Apart from anti-inflammatory potential, ELA reported for its biological effect as an anticancer, antidiabetic, antioxidant, cardioprotective, neuroprotective and hepatoprotective agent. In clinical trials ELA reduced blood glucose on insulin resistant diabetic subjects, it was enhanced motile spermatozoa count in human fertility study and significantly reduced blood pressure in cardiovascular disease study^60^. QCT also reported for its therapeutic effect on diabetes associate with obesity and circulatory dysfunction, cardiac diseases, tumour, oxidative stress, and microbial infection. It inhibited nucleic acid synthesis and interrupts plasma membranes function in bacteria, it down regulated CDK2, cyclins A and B and upregulated p53, p58, p21, p27 in different cancer cells^61^. COX-3 exhibits physiological and pathophysiological significance relating to its expression, which varies according to cell type. The physiological roles of COX-3 include the triple response (nociception, pyresis and inflammation), thrombosis, vascular tone, ovulation, implantation, angiogenesis, parturition, etc.^6,7,62–66^.

## Conclusion

The exact role of human COX-3 is unclear to date. In the current work, the effect of ZBN, ELA, and QCT was checked first in in-vitro then in-silico and in-vivo studies have provided enough evidence that proved in humans COX-3 is a potential target for the treatment of inflammation, and Pain. The CFA induced COX-3 overexpression inhibited by ELA and QCT among all parameter studied in present work like NFκB, TNF-α and IL-1β. However, ZBN inhibited CFA induced NFκB overexpression and thereby TNF-α and IL-1β downregulation. Indeed, COX-3 is an isoform of COX-1, which has well-established homeostasis functions in various tissues. Thus, further studies using anti-inflammatory models would be useful in designing suitable strategies for clinical studies and associated disease conditions. The structure–function correlation with other isoforms of cyclooxygenases would provide further insight into the establishment of an appropriate mechanism of action with reduced or mitigated side effects. Finally, our findings further bolster the notion that an appropriate derivative of zerumbone would be essential for pharmacological action.

**Table 1:**
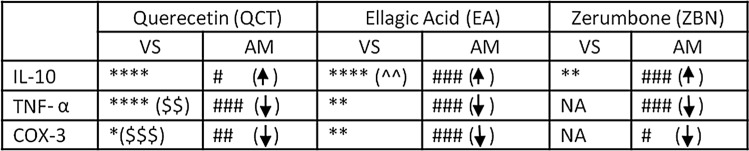
In-vitro (Virtual Screening-VS) and in-vivo (Animal Model-AM) prediction of drug (QCT, EA, ZBN)-receptor (IL-10, TNF- α, COX-3) interaction based on docking and up-down regulation in animal screening.

Up-regulation indicated by up arrow ( ) while down-regulation by ( ). Additional formation of bond with aromatic ring is represented by ($: π–π stacking bond) and (^: π–Cation bond).

### Supplementary Information

Supplementary Information.
