# Supplementary material for: Phytochemical mediated modulation of COX-3 and NFκB for the management and treatment of arthritis
Source: Sci Rep. 2023 Aug 21;13:13612. doi: 10.1038/s41598-023-37729-2 (PMC10442333; doi:10.1038/s41598-023-37729-2)
Supplement: Supplementary file 1 — Supplementary Information. [file 41598_2023_37729_MOESM1_ESM.docx]

Supplementary Information for

**Phytochemical Mediated Modulation of COX-3 and NFκB for the Management and Treatment of Arthritis**

Dipak Biswas, Bharat Gopalrao Somkuwar, Jagat Chandra Borah, Pritish Kumar Varadwaj, Saurabh Gupta, Zeeshan Ahmad Khan, [Gopinath](http://en.ustc.findplus.cn/?h=search_list&query=AR:%22Mondal%2C%20Gopinath%22) Mondal, [Asamanja](http://en.ustc.findplus.cn/?h=search_list&query=AR:%22Chattoraj%2C%20Asamanja%22) Chattoraj, Lokesh Deb*

Lokesh Deb

Email: [lokeshdeb.ibsd@nic.in](mailto:lokeshdeb.ibsd@nic.in)

**This file includes:**

Supplementary text

Figures S1 to S38

Tables S1 to S5

References for SI reference citations

**Other supplementary materials for this manuscript include the following:**

Datasets (R code) RS1 to RS4

**Also included**

Western blotting original images

Raw Original data of in-vitro anti-inflammatory and in-vivo anti-arthritic studies

Supplementary Information Text

**Subhead: Molecular docking, Molecular Dynamic Simulation and Statistical annotations.**

The discovery and function of the COX-3 isoform have been debated among the scientific community [1]. Its weaker inhibition by acetaminophen, apart from other theories of acetaminophen metabolization to N-arachidonoyl phenylamine by COX-1- and COX-2-mediated mechanisms cannot rule out the existence of novel cyclooxygenase isoforms [1, 2]. Experimental evidence from cell inhibition and bioassays has confirmed the presence of new cyclooxygenases in canines, rats, mice, and humans [3-7]. Some studies strived to show that the additional introns present in human COX-3 contribute to its gene expression. Also, an additional base pair at the intron leads to premature termination of COX-3 mRNA; thus, transcription of COX-3 often terminates at exon 2. However, recent advances in molecular biology, such as ribosome profiling (editing), ribosomal frameshifting and intron splicing modulators, have been used to overcome aberrant mRNA expression termination and achieve full mRNA expression, which subsequently proves the presence of COX-3 [2-7]. Its existence is further bolstered by the presence of a thermostable pseudoknot that might function as a repair mechanism [7]. Cyclooxygenase contains four sites of interaction: intron 1, the dimerization site, the membrane binding site and the catalytic site, each of which exerts a specific role. The non-coding intron 1 region regulates the transcription and translation of COX-3 [4]. The distinct function of COX-3 can be monitored by the regulation of its expression in various tissues [5, 8]. Posttranscriptional and translational elements may be responsible for the modulation of cyclooxygenase signaling [9]. Intron 1 has high GC content (68.4%), a steam loop, and a thermodynamic stability of -8.6 Kcal/mol and functions to regulate DNA transcription or RNA translation, as a checkpoint for the synthesis of either COX-1 or COX-3 and blocks the transfer of RNA to other subcellular compartments, which prevents glycosylation [10]. The glycosylation site is essential for the proper folding and catalysis of COXs, and COXs have also exhibited peroxidase activity [11-13]. The upregulation of cyclooxygenases indicates its active role based on the cumulative, co-operative and/or additive pathophysiological effects in normal neural cells [10]. Apart from its physiological and pathophysiological functions, COX-3 shows peculiar features compared to those of the other cyclooxygenase isoforms, which support its function as a potential receptor. It has the capability to cross the blood-brain barrier and produce the desired action, has analgesic and anti-inflammatory activity in both the central and peripheral nervous systems, and exhibits a higher affinity for acetaminophen (paracetamol) than COX-1 and COX-2, thus making COX-3 a preferred nociceptor target. Additional cofactors (e.g., glutathione or epinephrine) are not required to produce the desired effects, and lower doses can elicit an effect [3, 5-6].

Initially, models of the target proteins COX-3, TNF-α, and IL-10 were obtained from a protein database (PDB). The initial screening of selected inhibitors with target protein were performed using the GLIDE module of the Schrodinger software package. A total of twenty-three ligands with a maximum of four stereoisomers was prepared for each ligand. All target proteins were prepared using the protein preparation wizard (PrepWizard) in Maestro (Protein Preparation Wizard 2015-2; Epik version 2.4). The grids for each protein were prepared using GLIDE to identify the site on the target proteins where the ligands can interact during the docking process. Extra precision (XP) docking was performed to determine the optimal docked complexes. The best docking complexes, based on the GLIDE docking score and conformation, were used for further MDS. MD simulations were performed for COX-3, TNF-α and IL-10 in complexes with ZBN, ELA, and QCT by using Desmond (Desmond Molecular Dynamics System, 2016). Systems equilibration was performed with NVT and NPT ensembles using the SHAKE algorithm, and the system temperature up to 300 K and pressure up to 1 bar were maintained. After equilibration, a 30-ns molecular dynamics simulation was executed for all complexes. After completion of the simulation, the trajectory files of each complex were used to calculate the protein and ligand root-mean-square deviation (RMSD), the protein and ligand root-mean-square fluctuation (RMSF), the protein-ligand interaction and ligand torsion. Simulation scatter line plots were plotted using R version 3.4.2, and the R-code used is appended as supplementary files (RS-1, RS-2, RS-3, RS-4).

The membrane-bound COX-3 is expressed in brain, heart, skeletal muscles, liver, stomach, and small intestine [2, 4]. COX-3 exhibits physiological and pathophysiological significance relating to its expression, which varies according to cell type. The physiological roles of COX-3 include the triple response (nociception, pyresisandinflammation), thrombosis, vascular tone, ovulation, implantation, angiogenesis, and parturition, whereas its pathophysiological functions include neoplasia, neurodegenerative disorders, etc. [2-6, 9, 14-15].

**Supplementary Information: FIGURES**

**
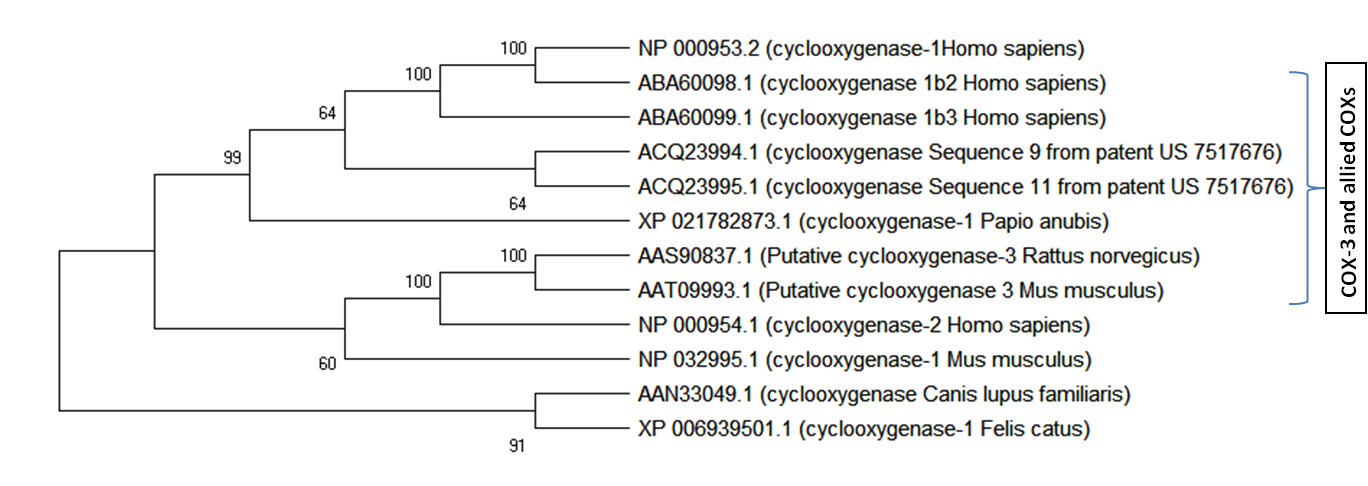
**

**Fig.S1.** Molecular Phylogenetic analysis by a Neighbour-joining method for cyclooxygenase and related proteins from various organisms. All the sequences obtained after dynamic alignment search of p-BLAST using Sequence 9 as query sequence from patent US 7517676 [16]. Multiple sequence alignment performed by ClustalWin a built plug-in of MEGA X software. All default parameters were accepted during analysis. The evolutionary history was inferred using the Neighbor-Joining method [17]. The bootstrap consensus tree inferred from 1000 replicates [17] is taken to represent the evolutionary history of the taxa analyzed [18]. Branches corresponding to partitions reproduced in less than 50% bootstrap replicates are collapsed. The percentage of replicate trees in which the associated taxa clustered together in the bootstrap test (1000 replicates) are shown next to the branches [18]. The evolutionary distances were computed using the number of differences method [19] and are in the units of the number of amino acid differences per sequence. The analysis involved 12 amino acid sequences. All positions containing gaps and missing data were eliminated. There was a total of 93 positions in the final dataset. Evolutionary analyses were conducted in MEGA X [20]. The cluster exhibits cyclooxygenase-3 as an isoform of COX-1.


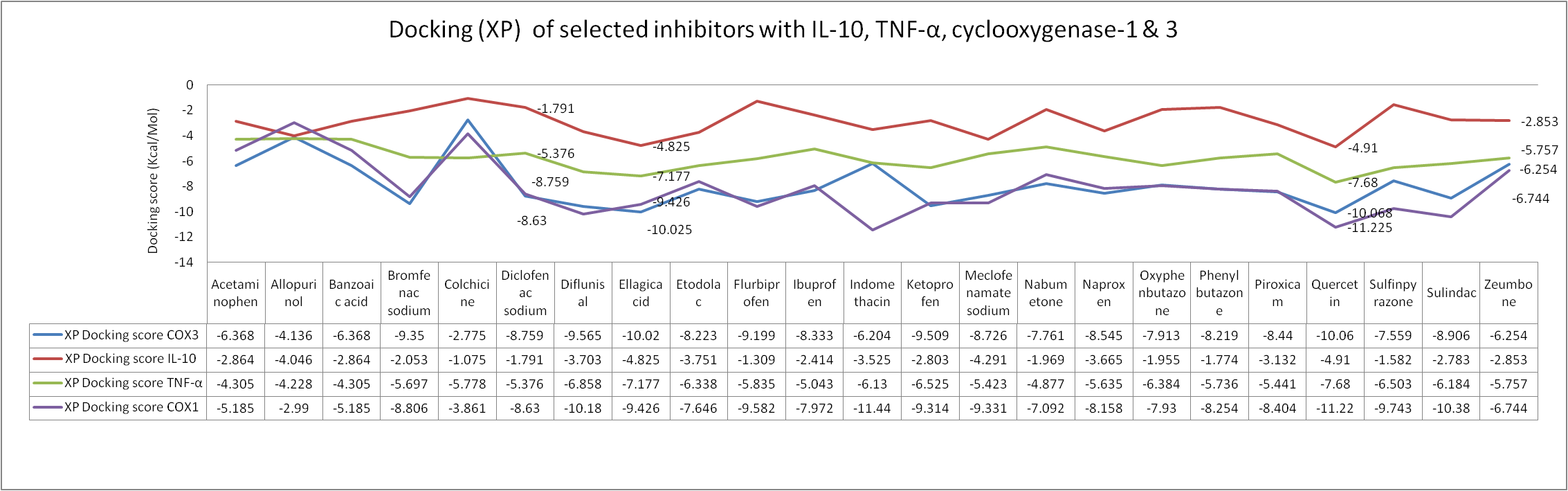


**Fig. S2. The target-ligand docking score monitored using standard docking procedure. Standard (selective, nonselective COX inhibitors) and natural ligands isolated in the present study were selected to decipher comparative inhibitory potential.**

XP (Extra Precision) docking score (Kcal/Mol) of COX-3 with Quercetin, Ellagic acid, Zerumbone,and Diclofenac Sodium complex is -10.068, -10.025, -6.25 and -8.759. Minimum docking score represents good binding affinities with the receptor. For TNF- α with Quercetin, Ellagic acid, Zerumbone,and Diclofenac Sodium complex is -7.68, -7.177, -5.757and -5.376 respectively. For IL-10 with Quercetin, Ellagic acid, Zerumbone, and Diclofenac Sodium complex are -4.91, -4.825, -2.835 and -1.791 respectively. For COX-1 with Quercetin, Ellagic acid, Zerumbone, and Diclofenac Sodium complex are -11.22, -9.426, -6.744 and -8.63 respectively.


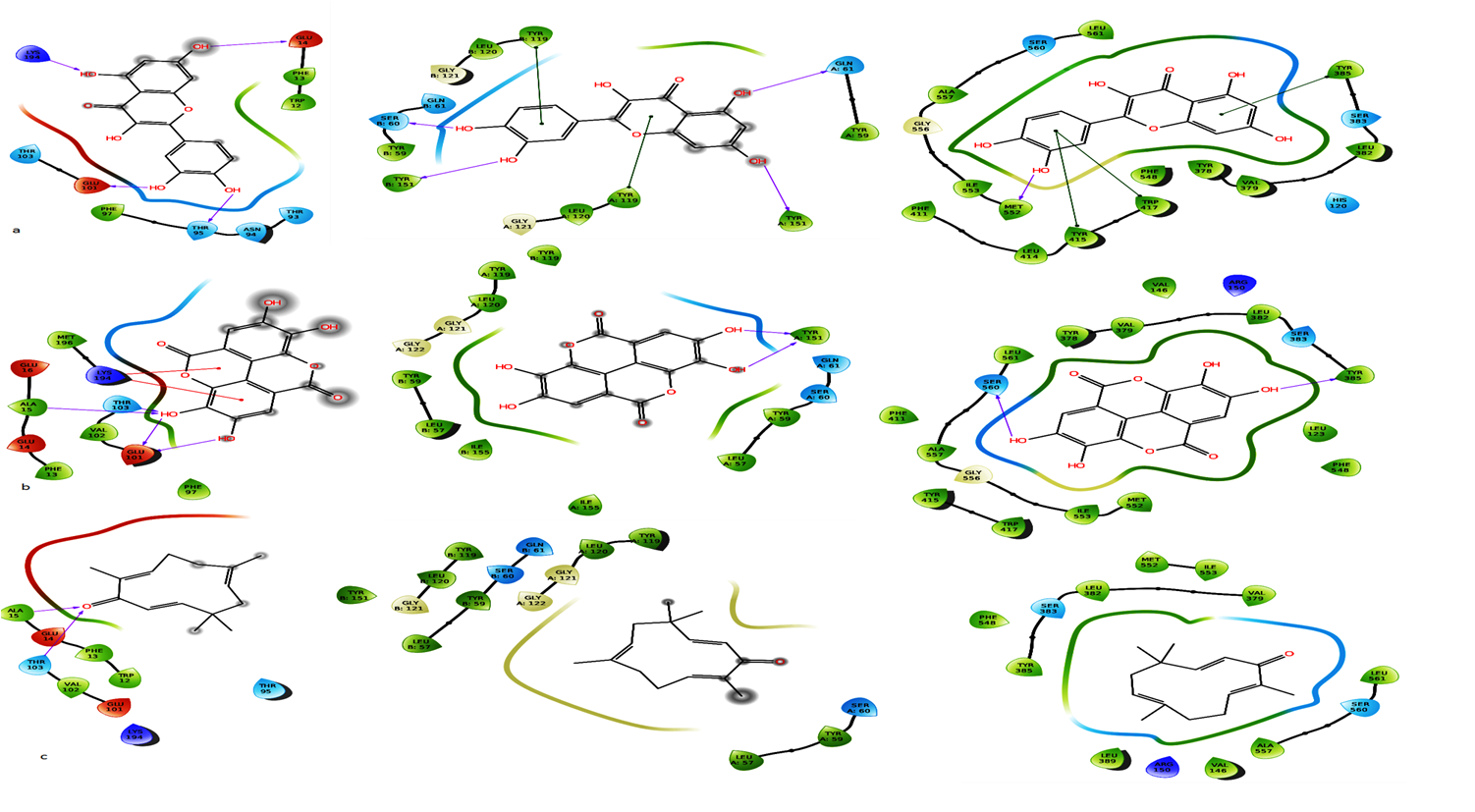


**Fig.S3.** The 2D hydrogen bond interaction of IL-10 with (a) Quercetin (b) Ellagic acid and (c) Zerumbone. TNF- α with (a) Quercetin (b) Ellagic acid and (c) Zerumbone. COX-3 with (a) Quercetin (b) Ellagic acid and (c) Zerumbone.

**
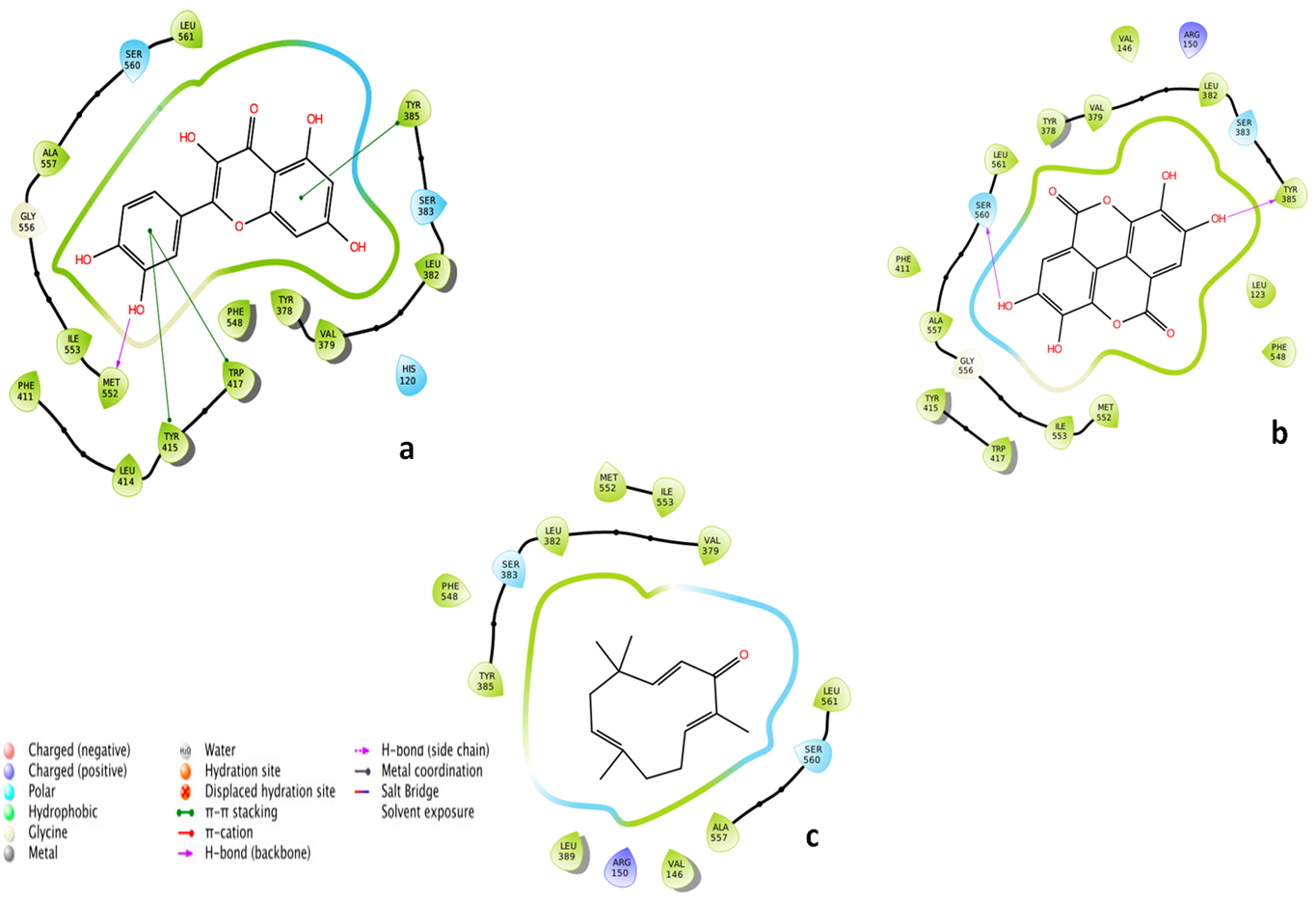
**

**Fig.S4.** The target-ligand Hydrogen bond interaction. (a) Quercetin-COX-3 (MET-552) (b) Ellagic acid-COX-3 (SER-560, TYR-385), (c) Zerumbone-COX-3 (NA) after MDS.

**
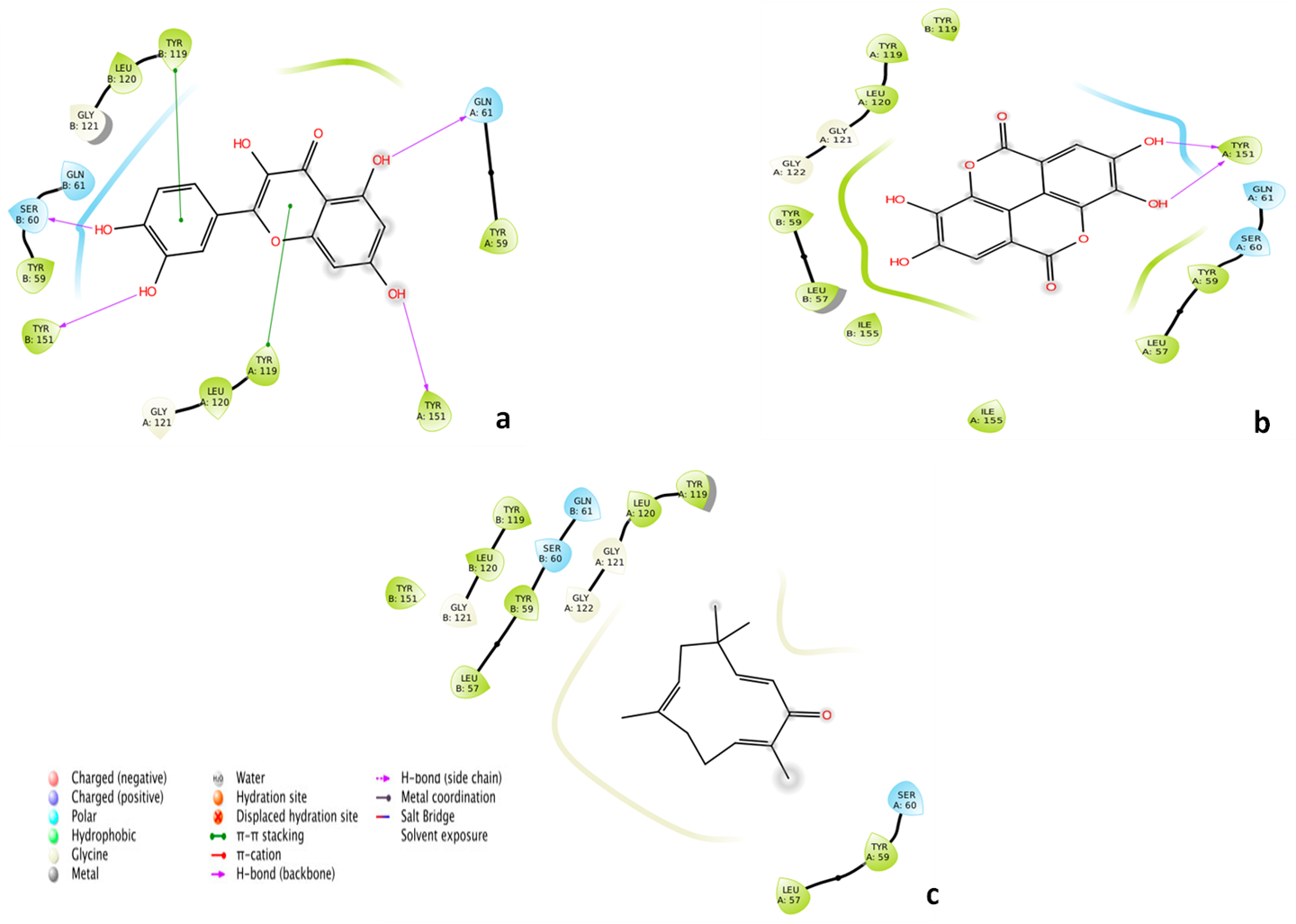
**

**Fig.S5.** The target-ligand Hydrogen bond interaction. (a) Quercetin- TNF- α (SER-60, TYR-151,GLN-61, TYR151) (b) Ellagic acid- TNF- α (TYR-151), (c) Zerumbone- TNF- α (NA) after MDS.

**
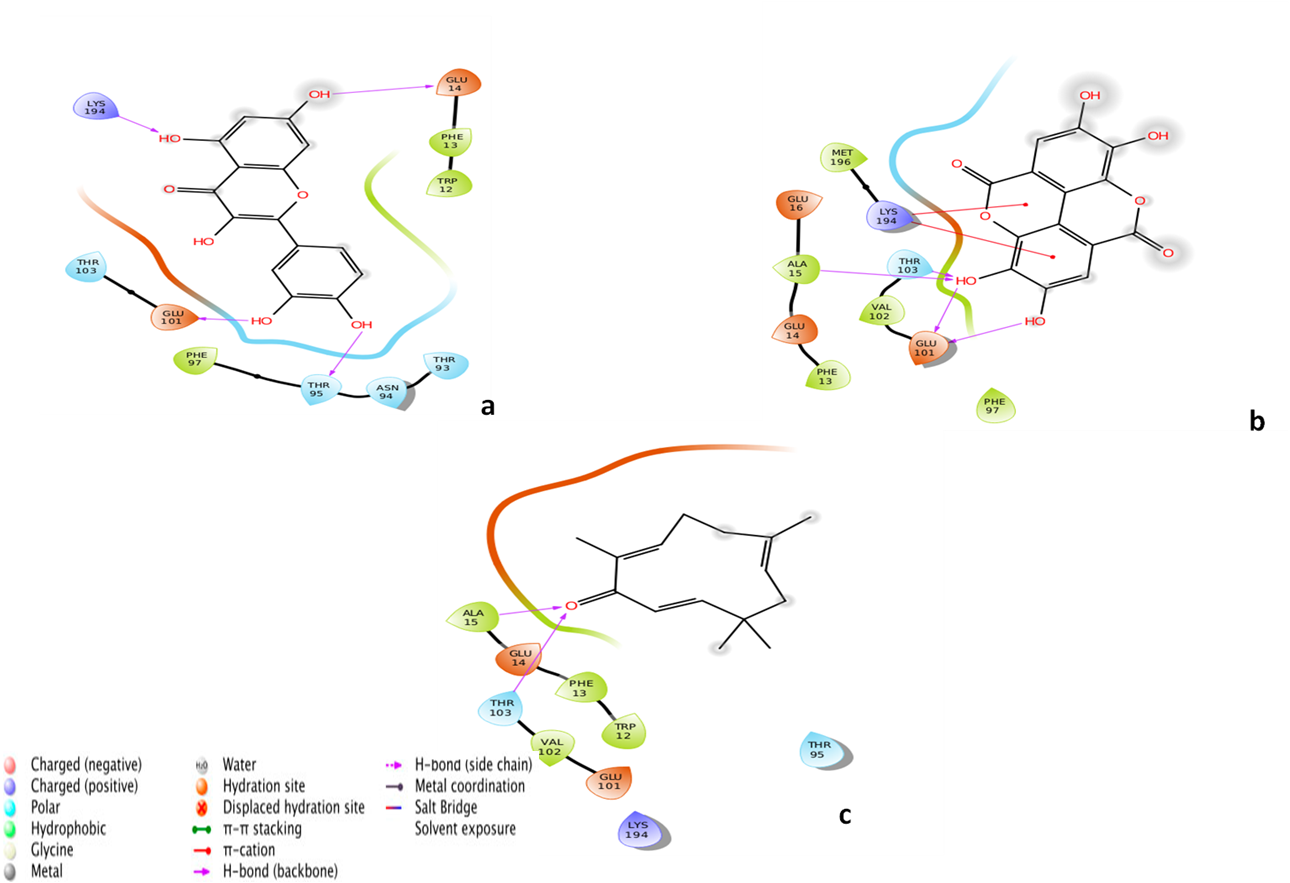
**

**Fig.S6.** The target-ligand Hydrogen bond interaction. (a) Quercetin- IL-10 (GLU-14, THR-95, GLU-101, LYS-194) (b) Ellagic acid- IL-10 (ALA-15, THR-103, GLU-101), (c) Zerumbone- IL-10(ALA-15, THR-103) after MDS

**
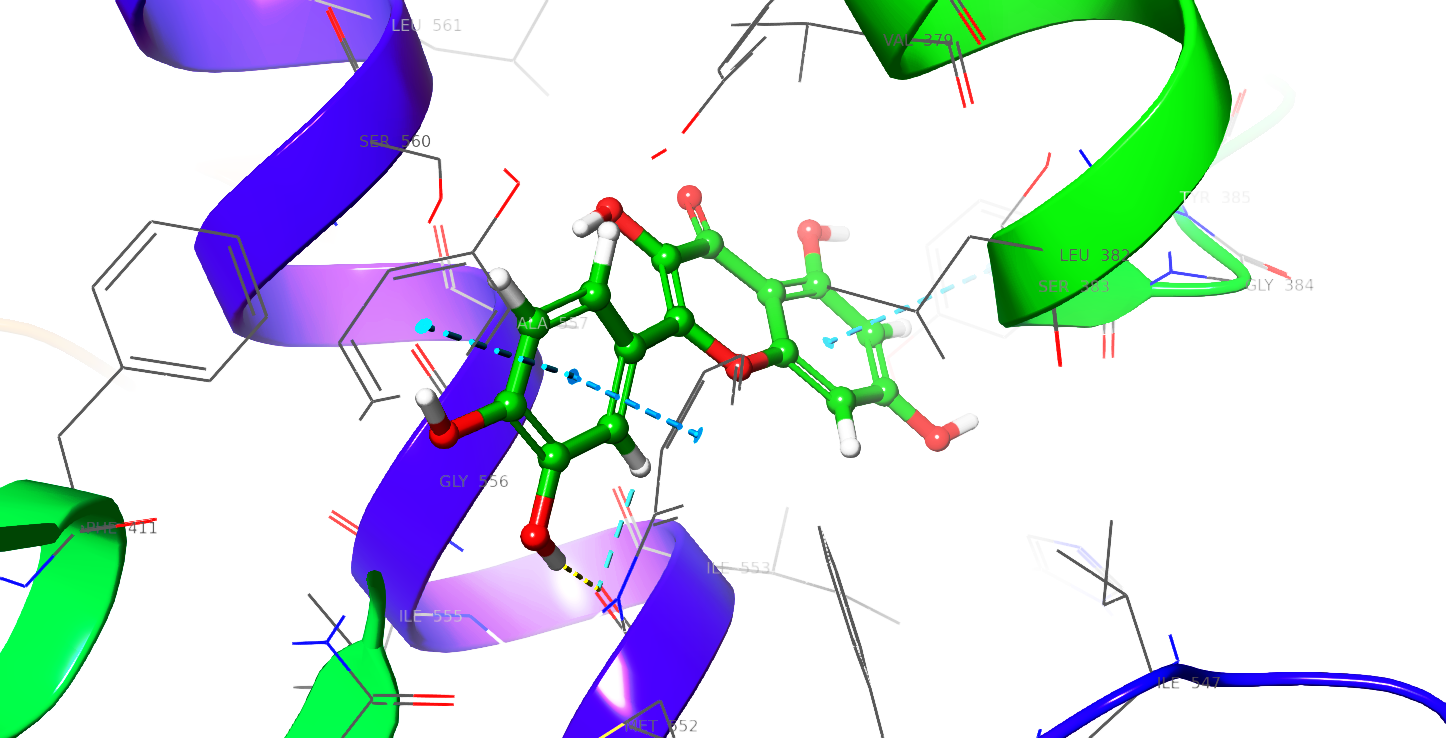
**

**Fig.S7.** The target-ligand Hydrogen bond interaction - Quercetin-COX-3 (MET-553) after MDS.

**
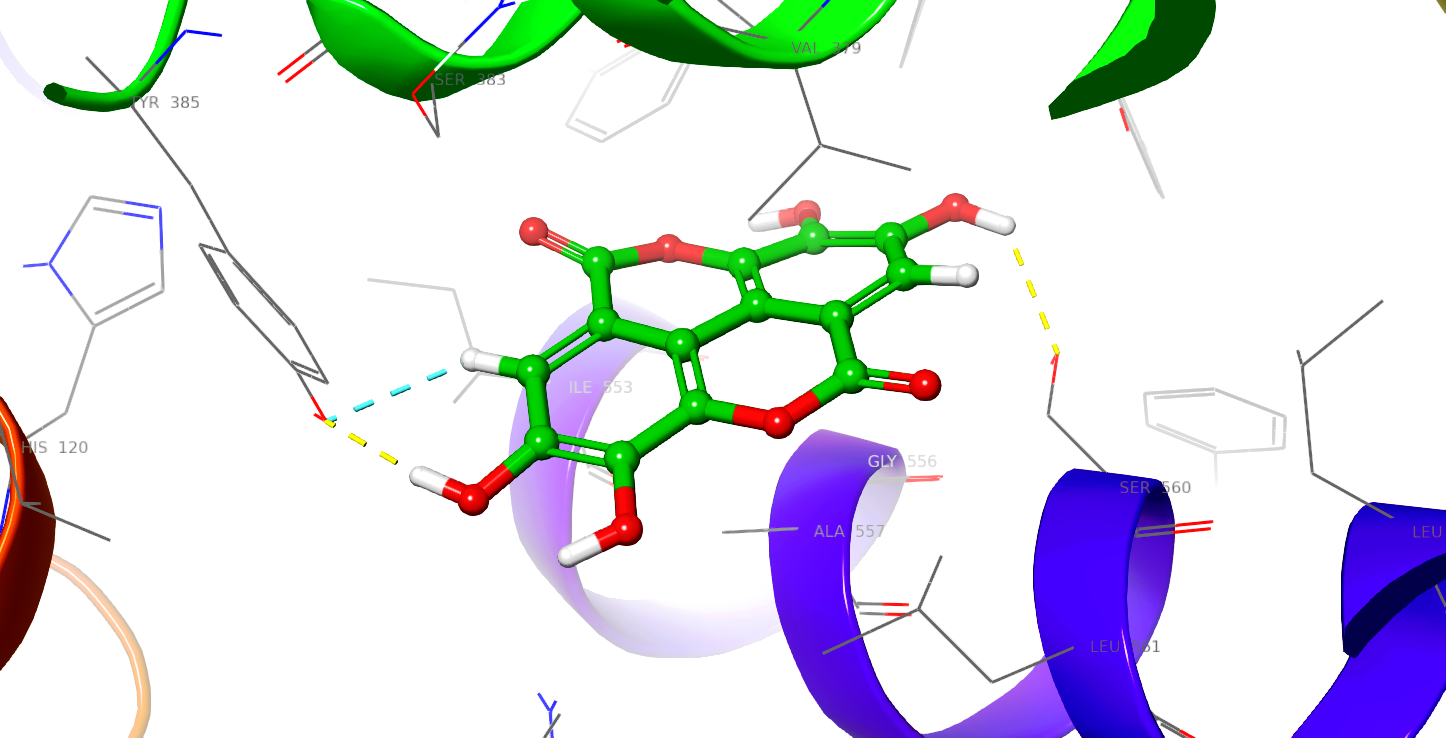
**

**Fig.S8.** The target-ligand Hydrogen bond interaction – Ellagic acid- COX-3 (SER-560, TYR-385) after MDS.

**
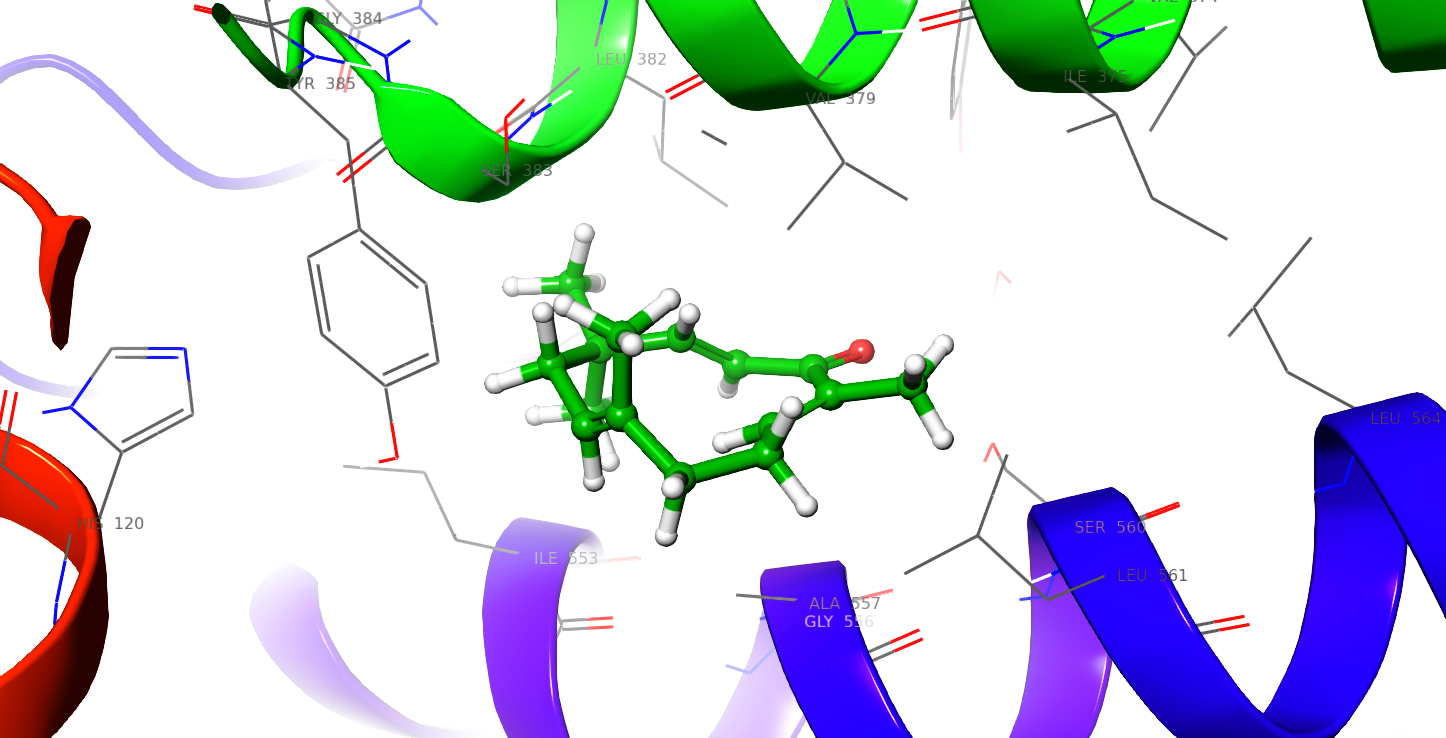
**

**Fig.S9.** The target-ligand Hydrogen bond interaction – Zerumbone - COX-3 (NA) after MDS


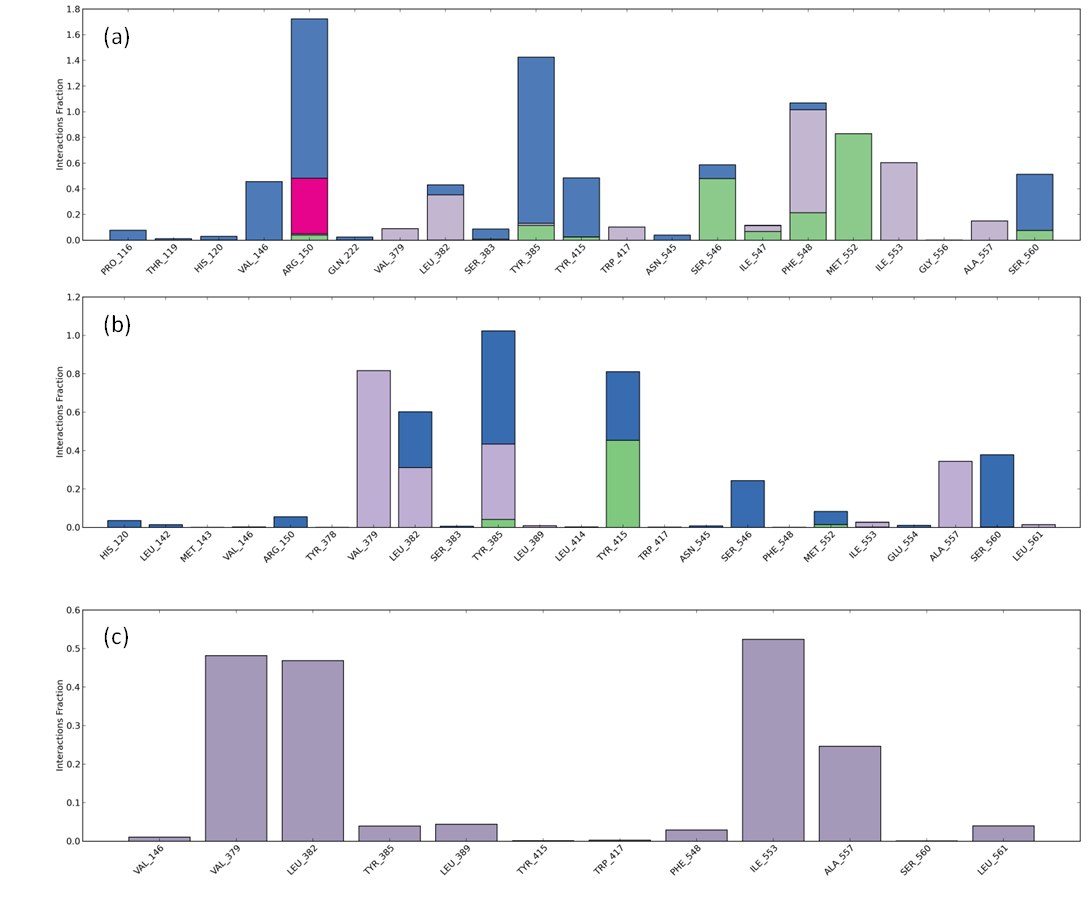


**Fig.S10.** Residual interaction fraction and amino acids histogram for COX-3 with (a) Quercetin (b) Ellagic acid and (c) Zerumbone after MDS.


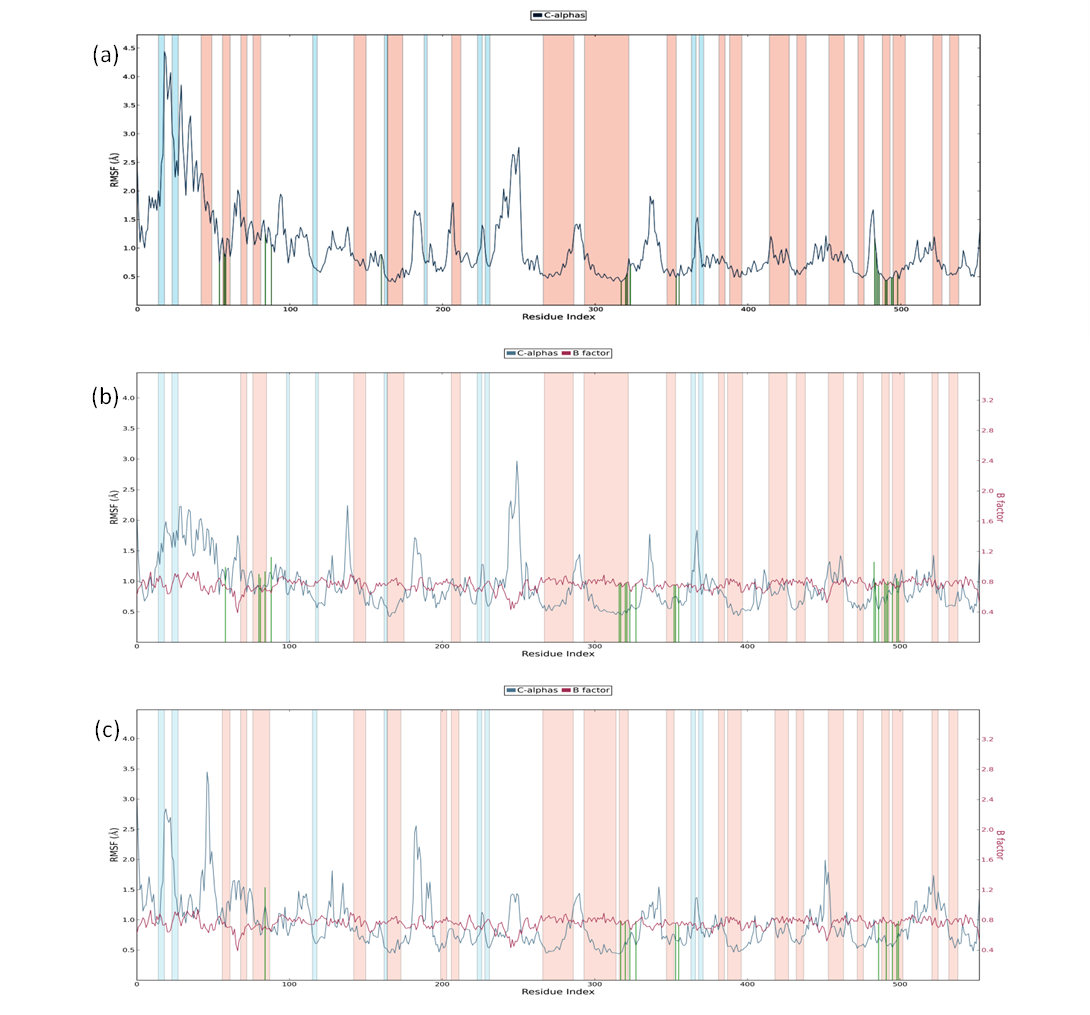


**Fig.S11.** RMSF (A^0^) with amino acids Residue Index for C alpha and B-factor of COX-3 with (a) Quercetin (b) Ellagic acid and (c) Zerumbone.


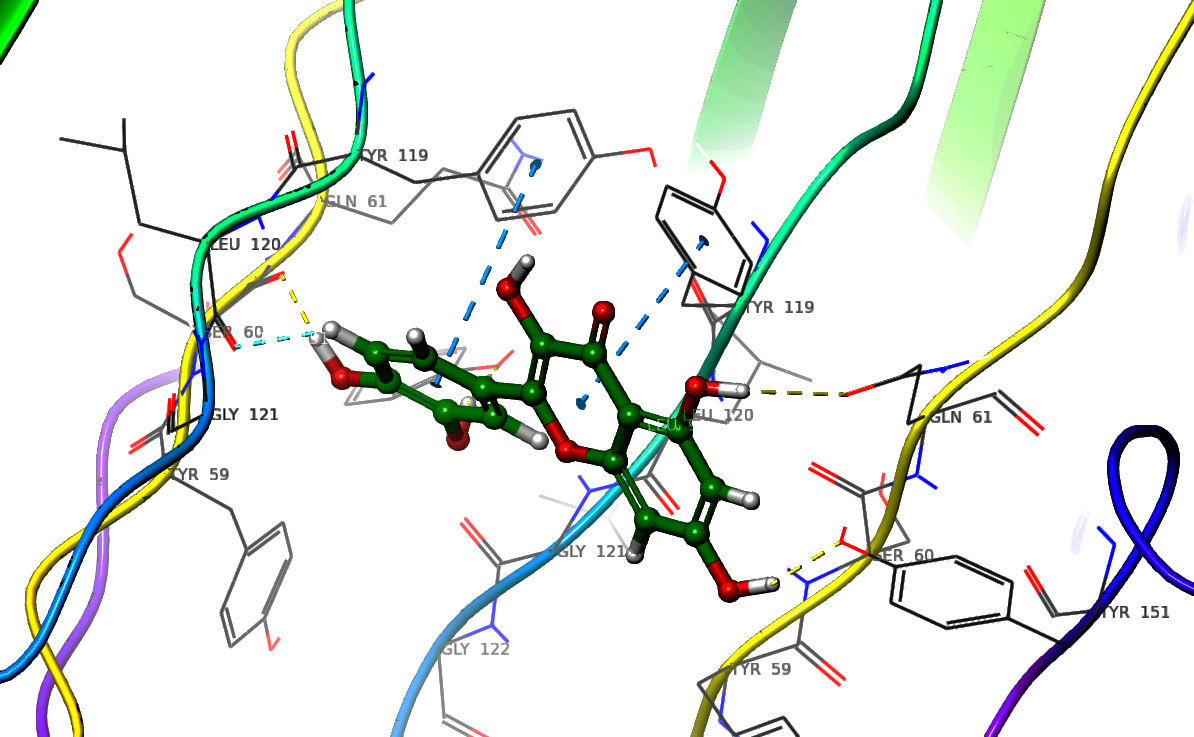


**Fig.S12.** The target-ligand Hydrogen bond interaction – Quercetin – TNF- α (SER-60, GLN-61, TYR-119, TYR-151) after MDS.

**
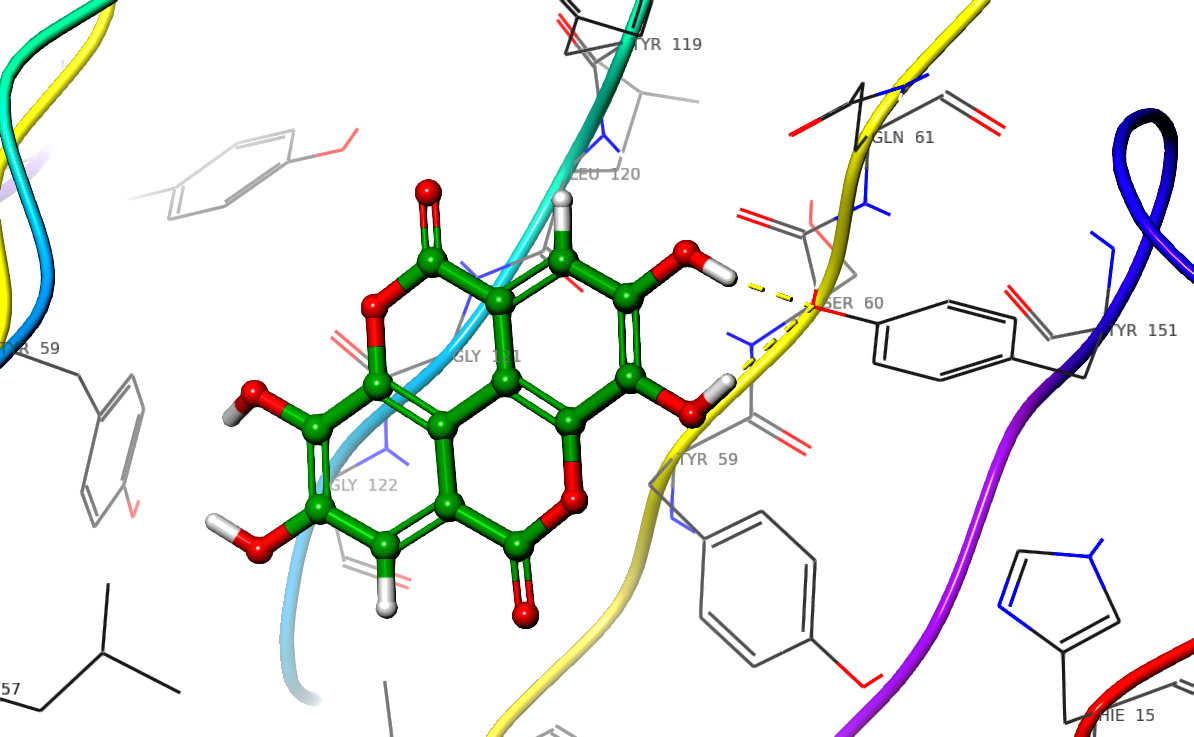
**

**Fig.S13.** The target-ligand Hydrogen bond interaction – Ellagic acid – TNF- α (TYR-151) after MDS.

**
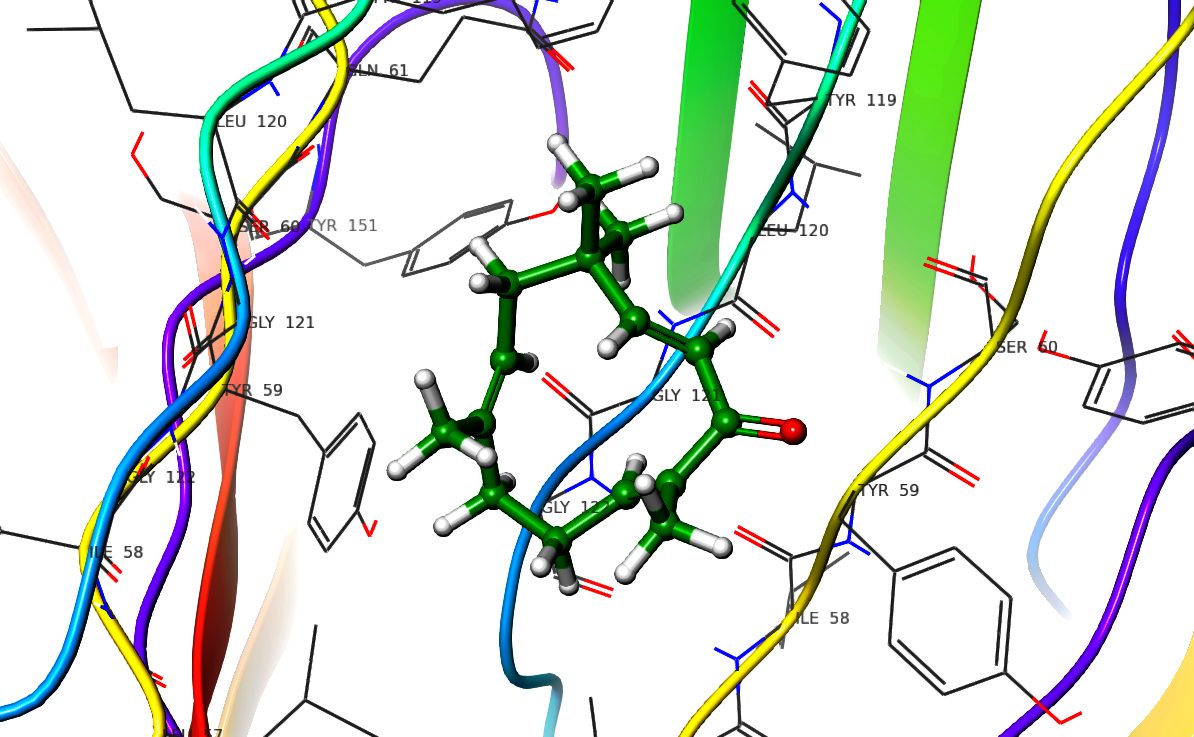
**

**Fig.S14.** The target-ligand Hydrogen bond interaction – Zerumbone – TNF- α (NA) after MDS.


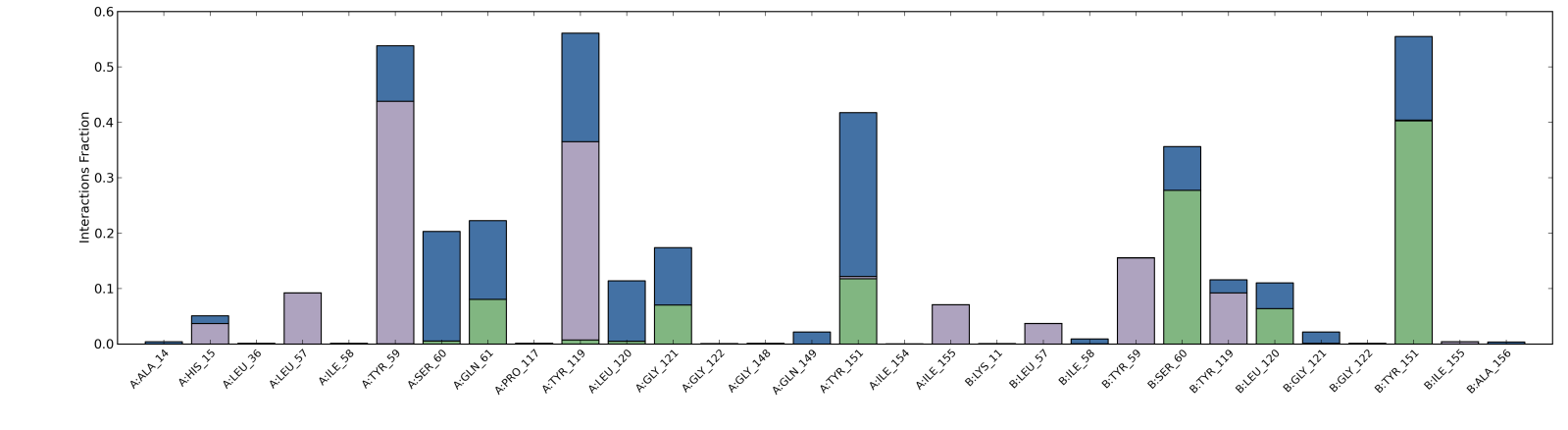

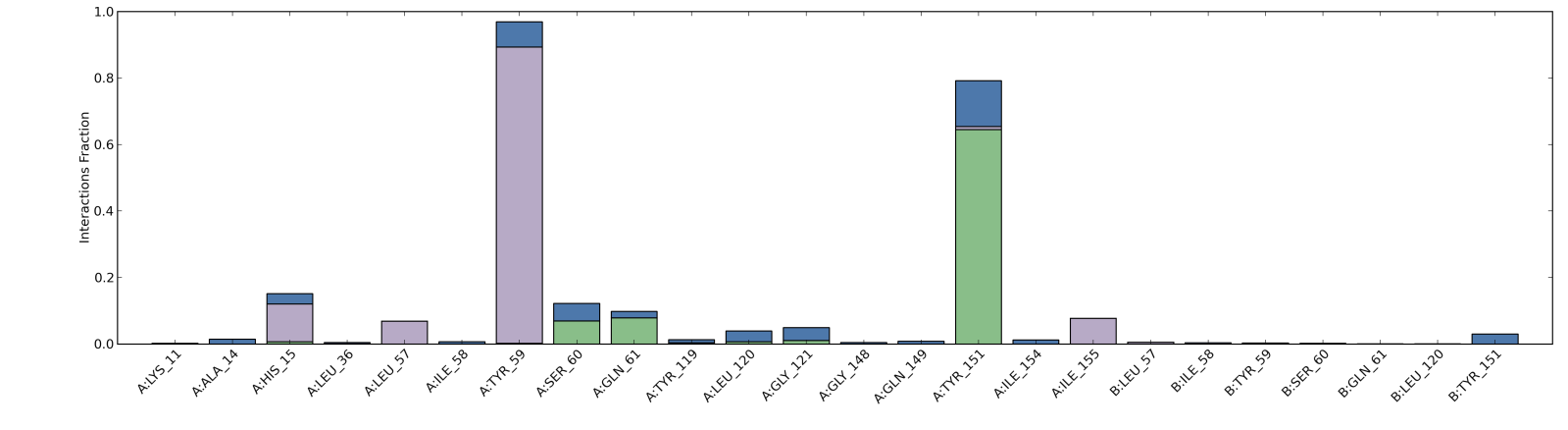

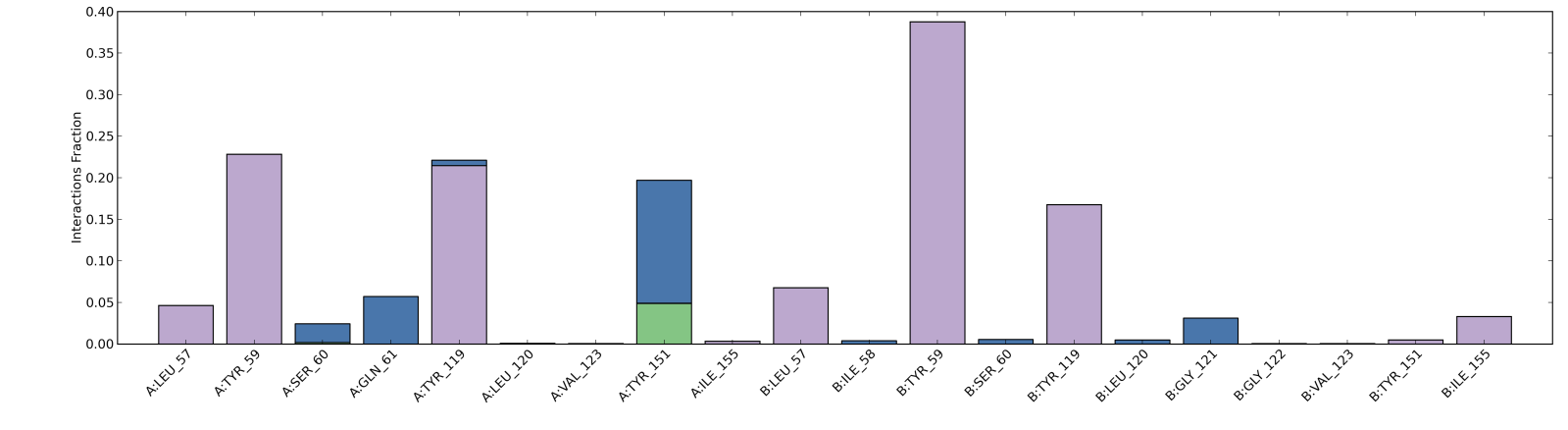


(a)

(b)

(c)

**Fig.S15.** Residual interaction fraction and amino acids histogram for TNF-α with (a) Quercetin (b) Ellagic acid and (c) Zerumbone.


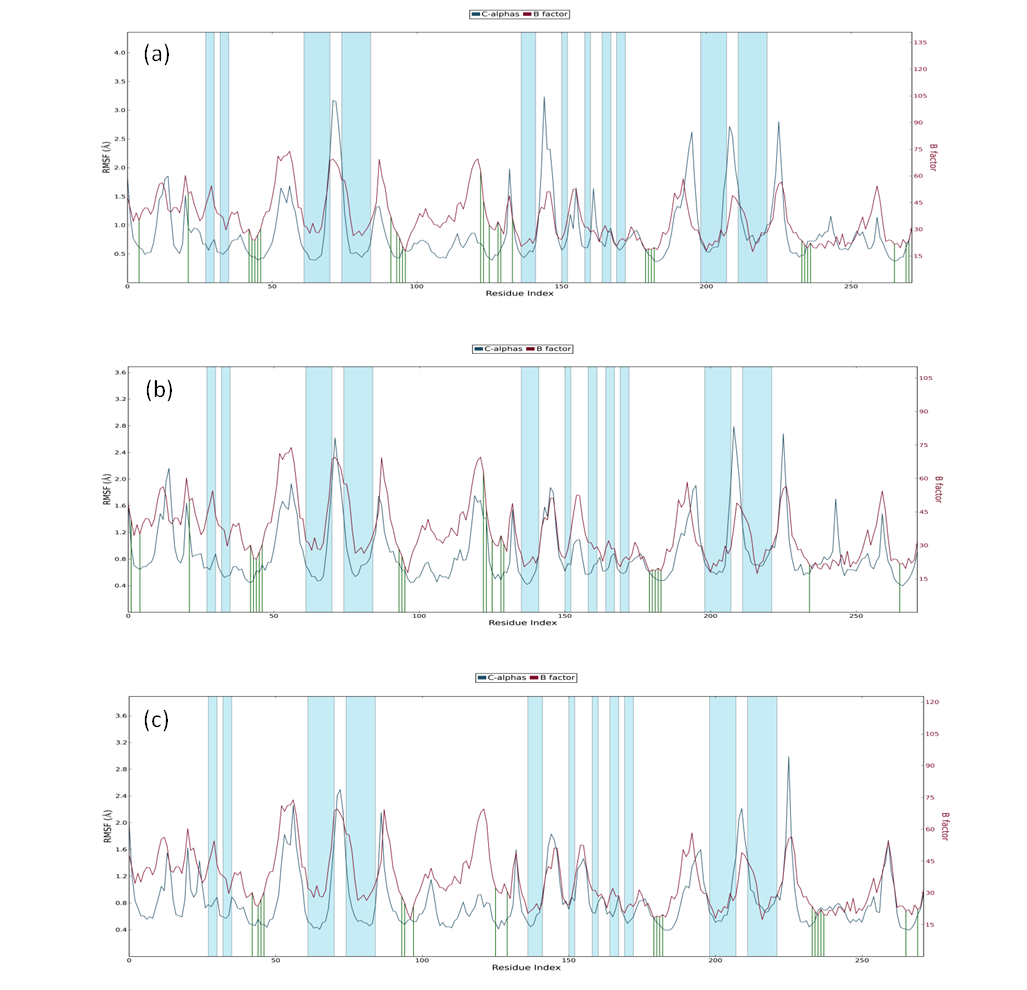


**Fig.S16.** RMSF (A^0^) with amino acids Residue Index for C alpha and B-factor of TNF-α with (a) Quercetin (b) Ellagic acid and (c) Zerumbone.

**
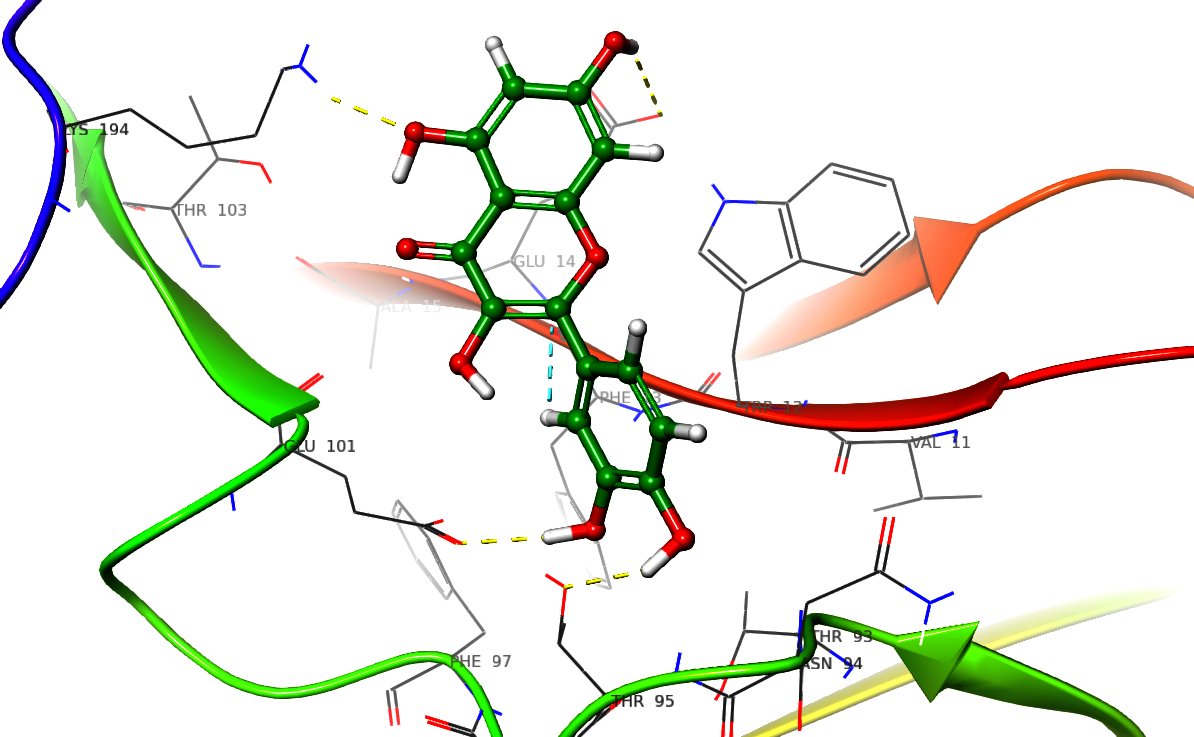
**

**Fig.S17.** The target-ligand Hydrogen bond interaction – Quercetin – IL-10 (GLU-14, THR-95, GLU-101, LYS-194).

**
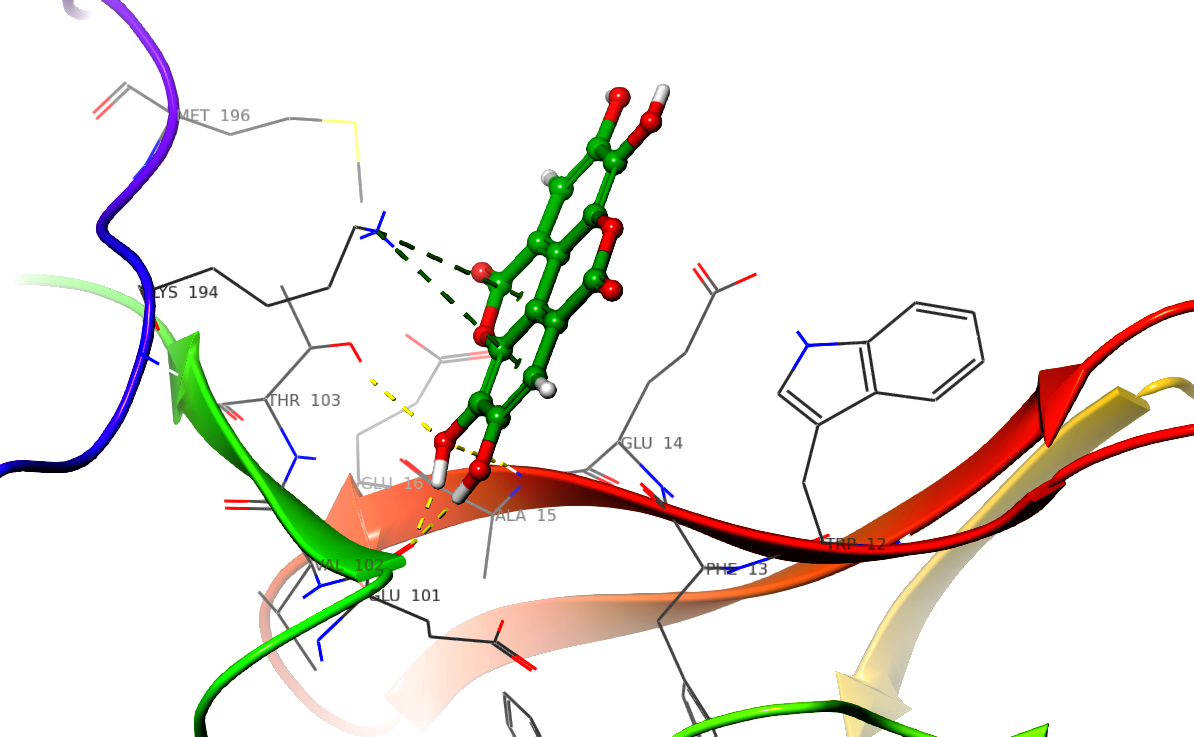
**

**Fig.S18.** The target-ligand Hydrogen bond interaction – Ellagic acid – IL-10 (ALA-15, GLU-101, THR-103).


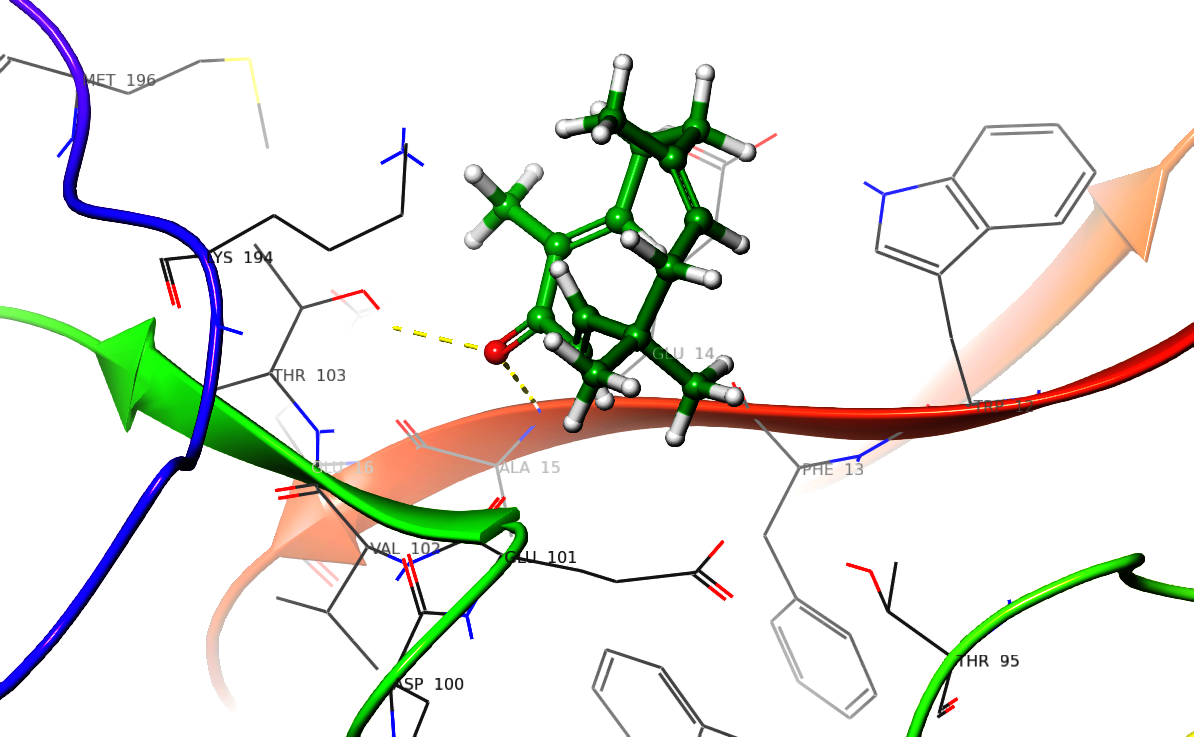


**Fig.S19.** The target-ligand Hydrogen bond interaction – Zerumbone – IL-10 (ALA-115, THR-103).


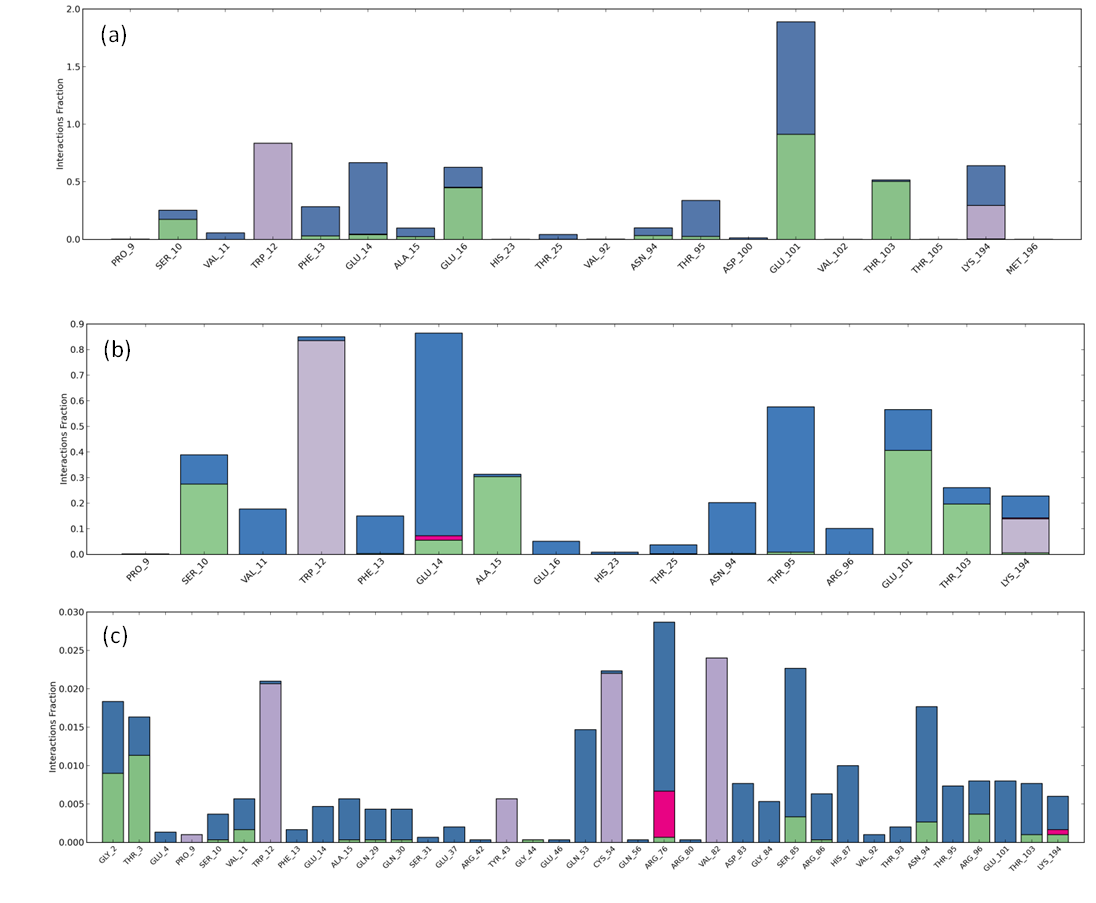


**Fig.S20.** Residual interaction fraction and amino acids histogram for IL-10 with (a) Quercetin (b) Ellagic acid and (c) Zerumbone.


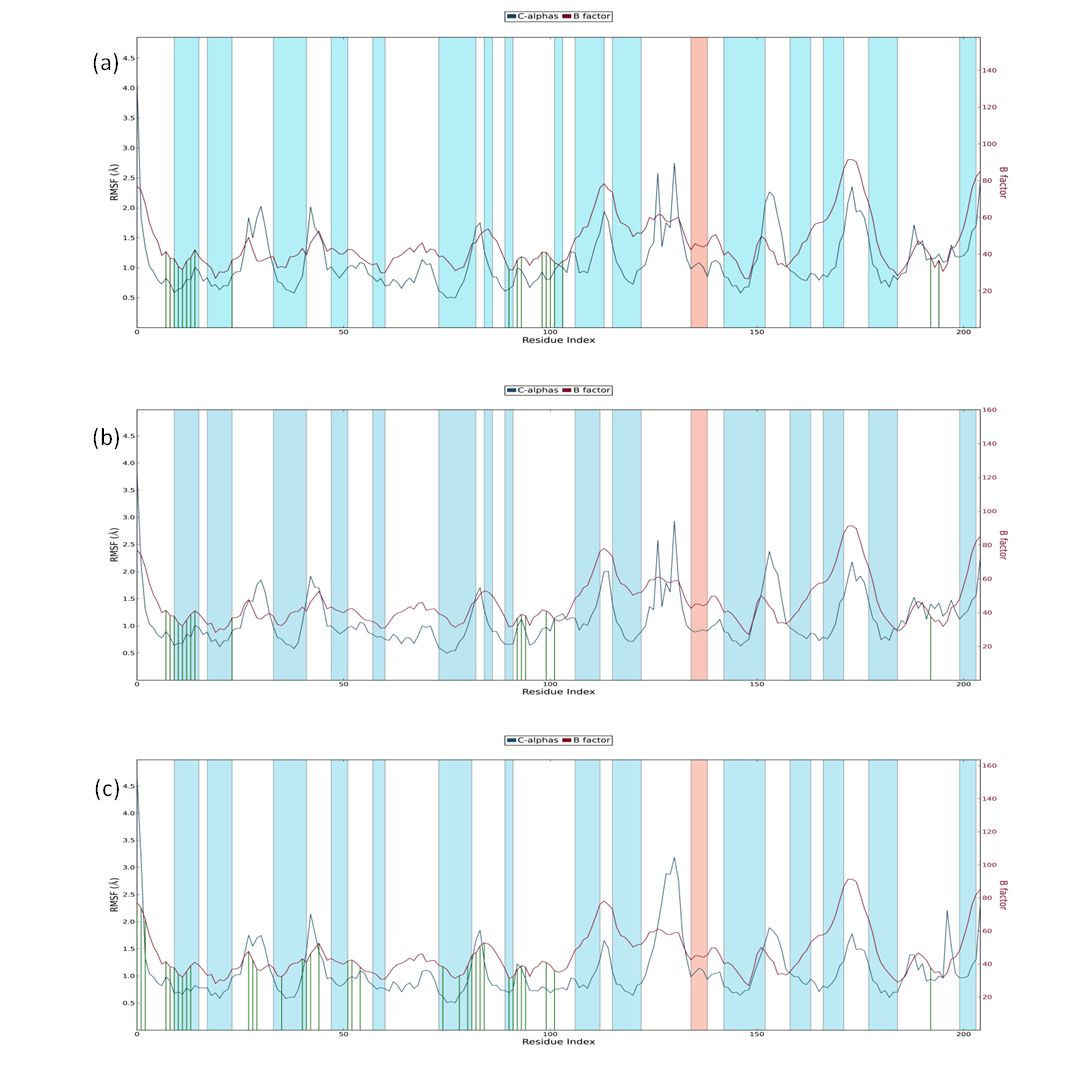


**Fig.S21.** RMSF (A^0^) with amino acids Residue Index for C alpha and B-factor of IL-10 with (a) Quercetin (b) Ellagic acid and (c) Zerumbone.


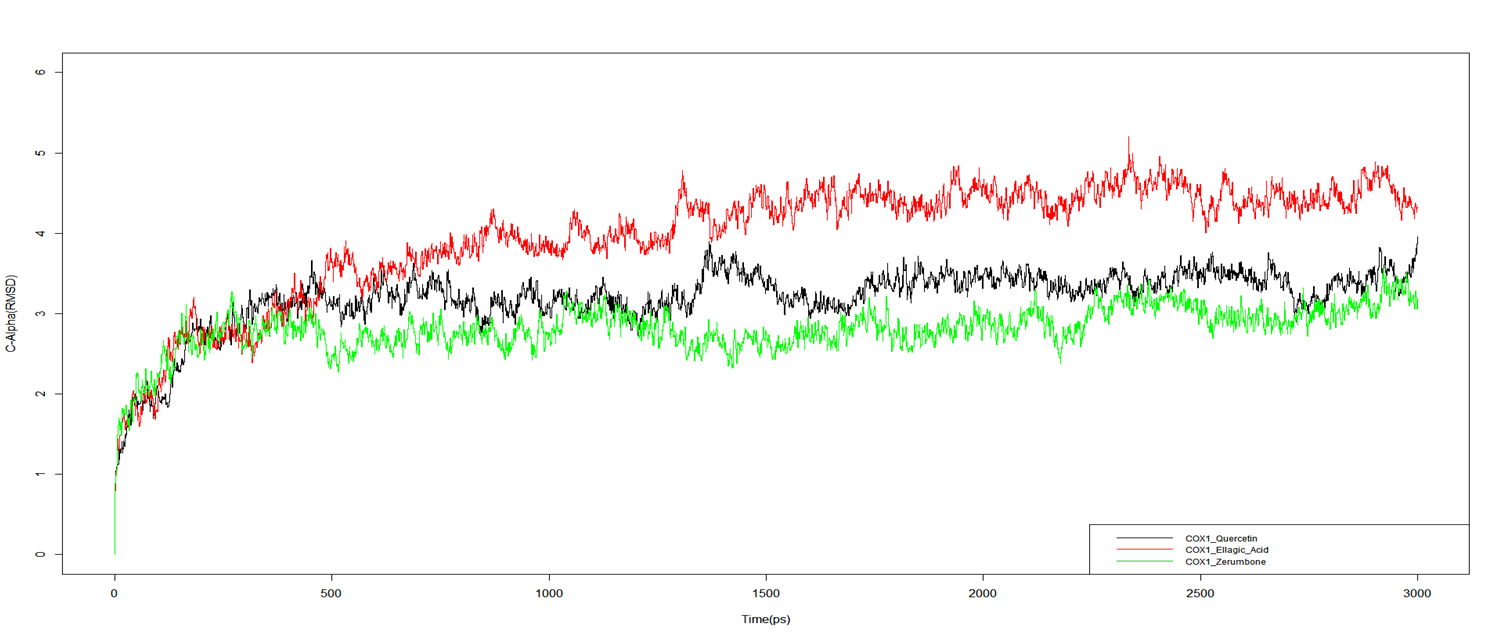


**Fig.S22.** The structural stability of model monitored by the molecular dynamics simulations of COX-1 with Quercetin, Ellagic acid, andZerumbone. Ellagic acid exhibits greater RMSD value as the time progresses (30 ns long MD trajectory) while Quercetin and zerumbone representing almost constant stability pattern at after 12 ns except for approximately 22ns. Only Quercetin achieves stable trajectory as time progresses.


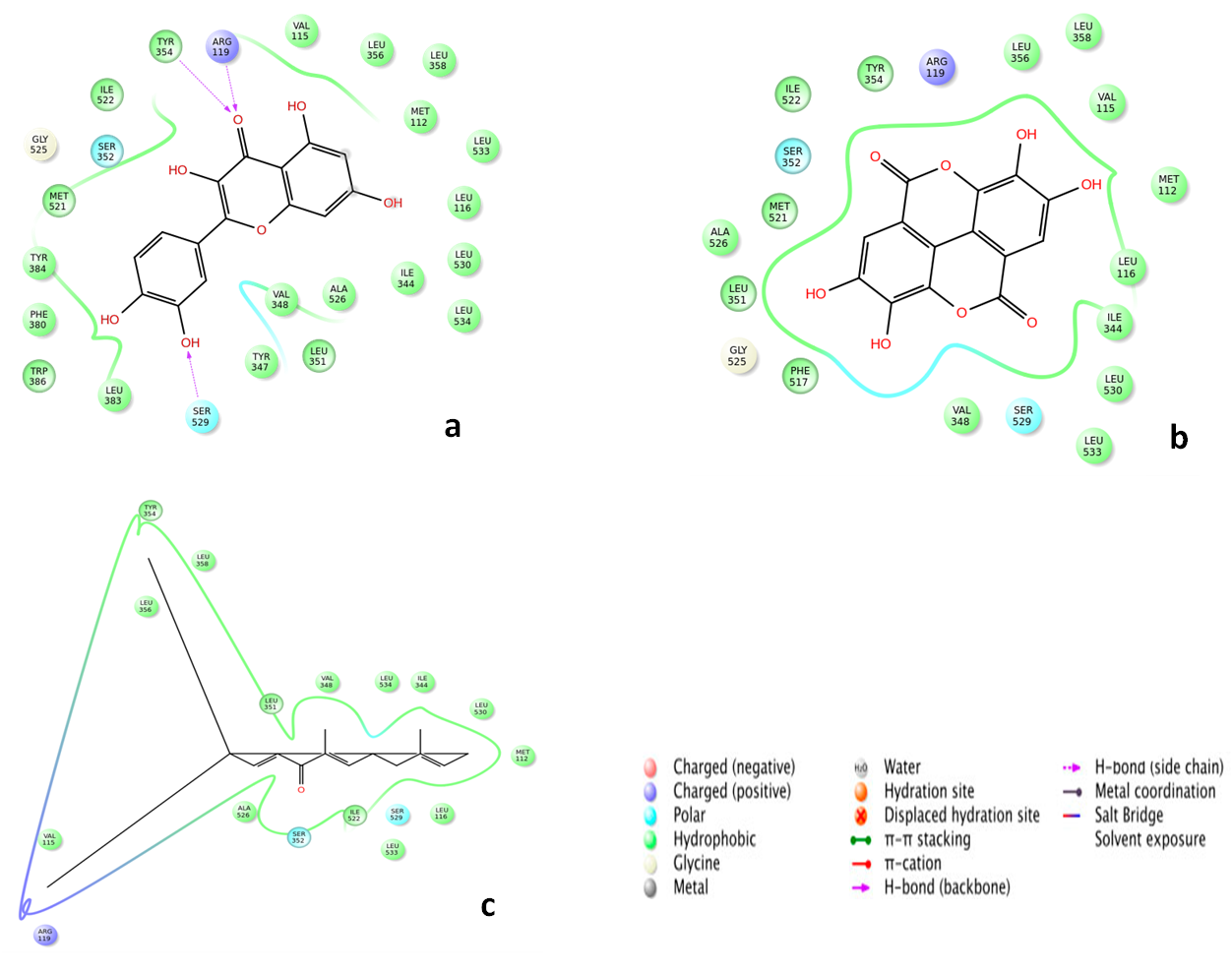


**Fig.S23.** The 2D hydrogen bond interaction of COX-1 with (a) Quercetin (b) Ellagic acid and (c) Zerumbone. Further corroborated by Partial Least Contacts (PLC) graphs.

**
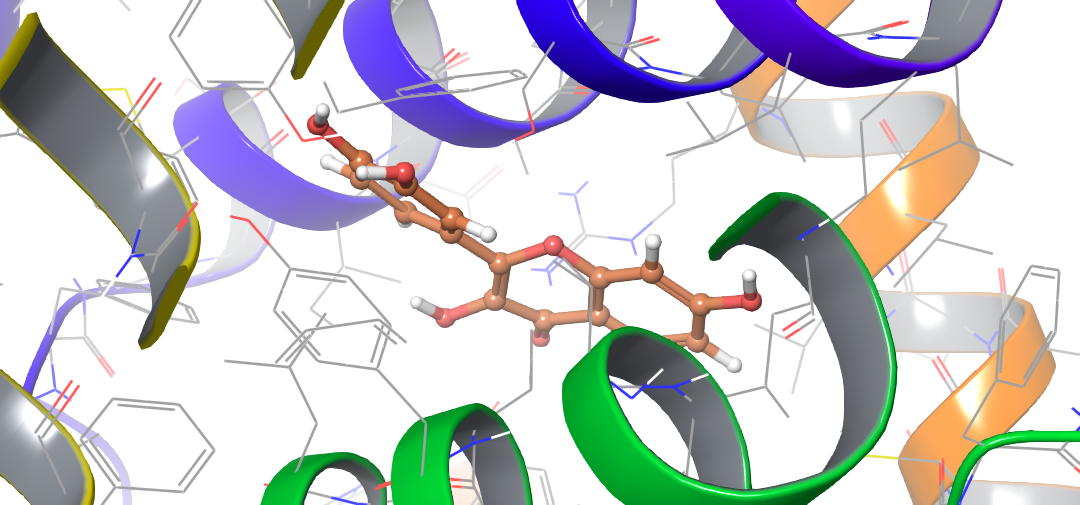
**

**Fig.S24.** The target-ligand Hydrogen bond interaction - Quercetin-COX-1 (ARG-119, TYR-354, and SER-529).

**
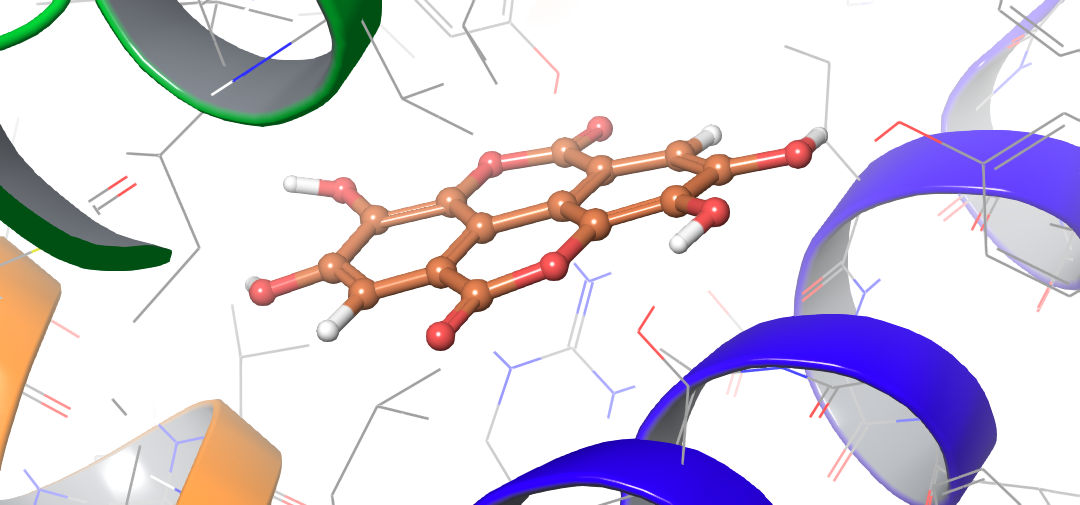
**

**Fig.S25.** The target-ligand Hydrogen bond interaction – Ellagic Acid- COX-1 (NA).

**
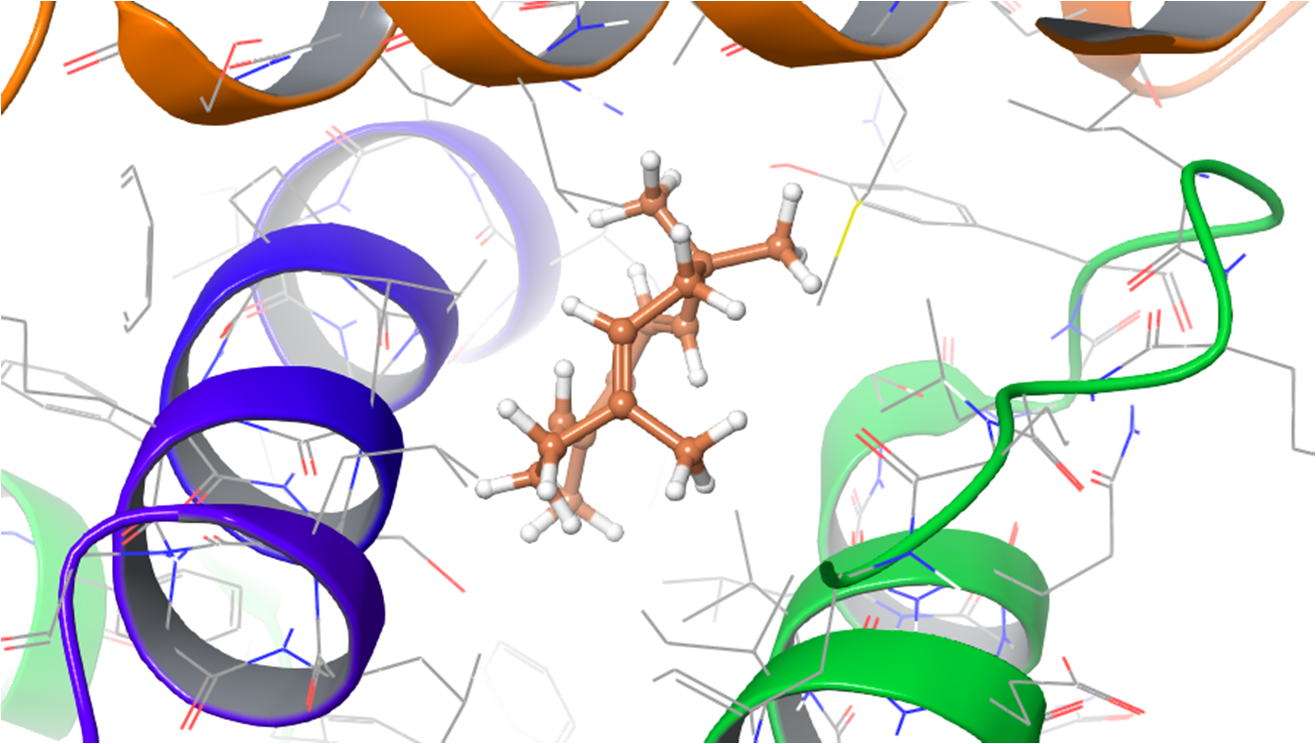
**

**Fig.S26.** The target-ligand Hydrogen bond interaction - Zerumbone- COX-1 (NA).

**
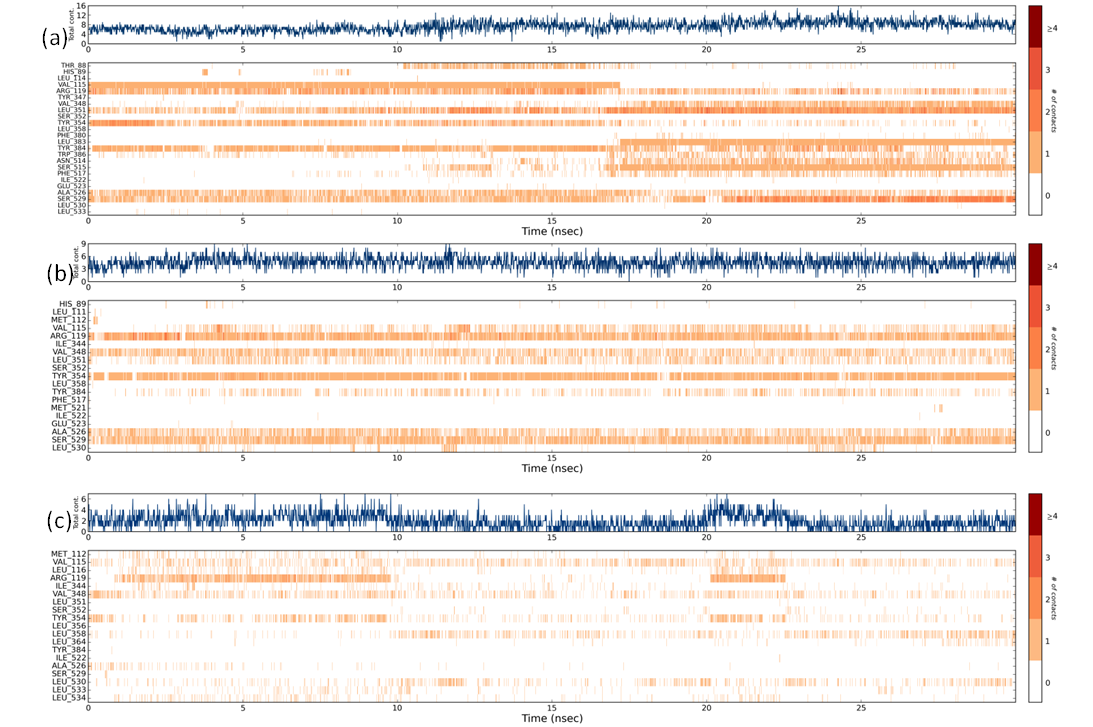
**

**Fig**.**S27**. Partial Least contact simulation graph at 30 ns with amino acids of COX-1 with (a) Quercetin (b) Ellagic acid and (c) Zerumbone. The scale on the right side of the graph indicates no of contacts (IL-10-Ellagic acid representing more number of PL contact simulation while IL-10 and Zerumbone no contact points the reason may be due to poor hydrogen bond interactions (No hydrogen bond donor).


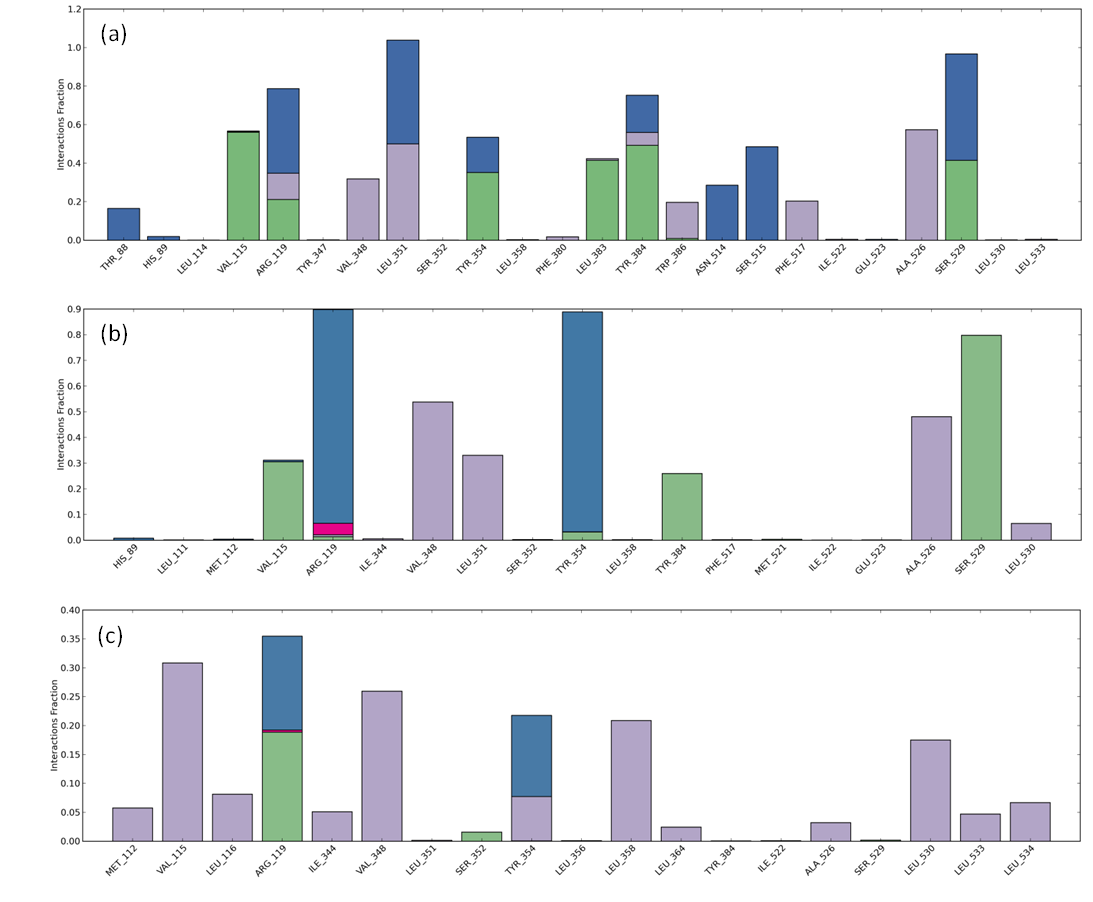


**Fig.S28.** Residual interaction fraction and amino acids histogram for COX-1 with (a) Quercetin (b) Ellagic acid and (c) Zerumbone.


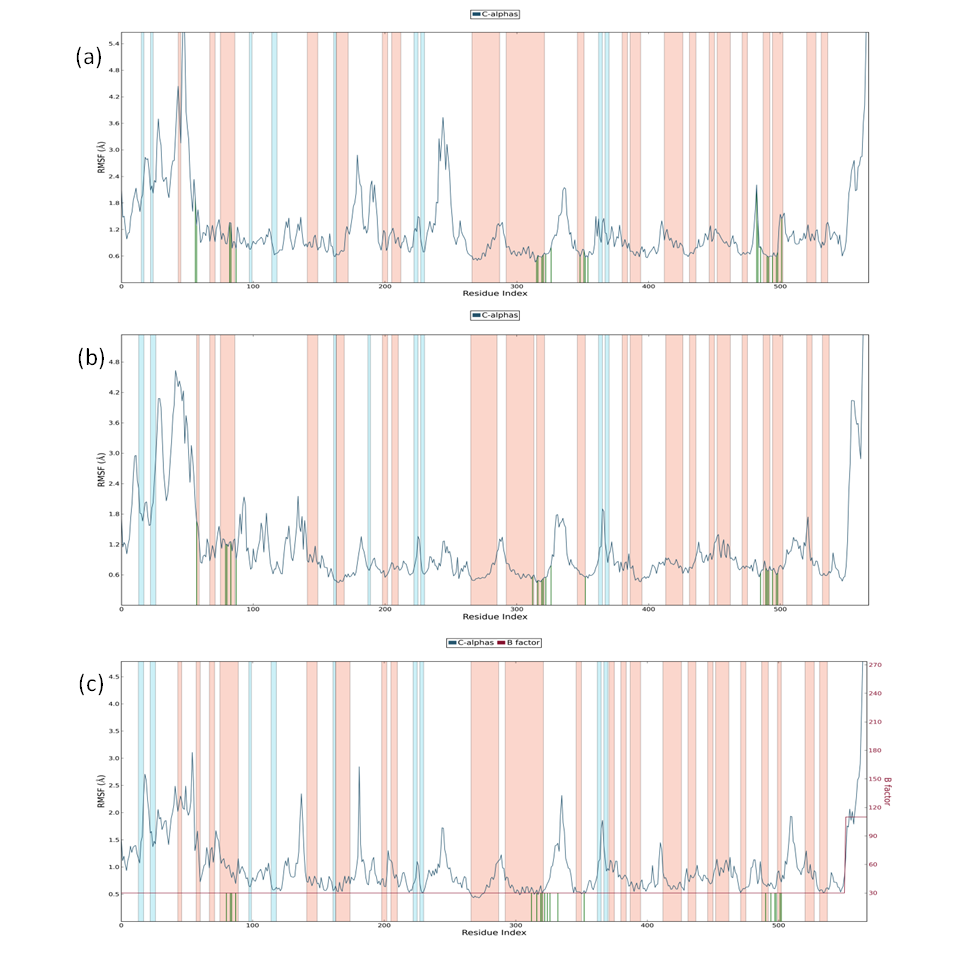


**Fig.S29.** RMSF (A^0^) with amino acids Residue Index for C alpha and B-factor of COX-1 with (a) Quercetin (b) Ellagic acid and (c) Zerumbone.


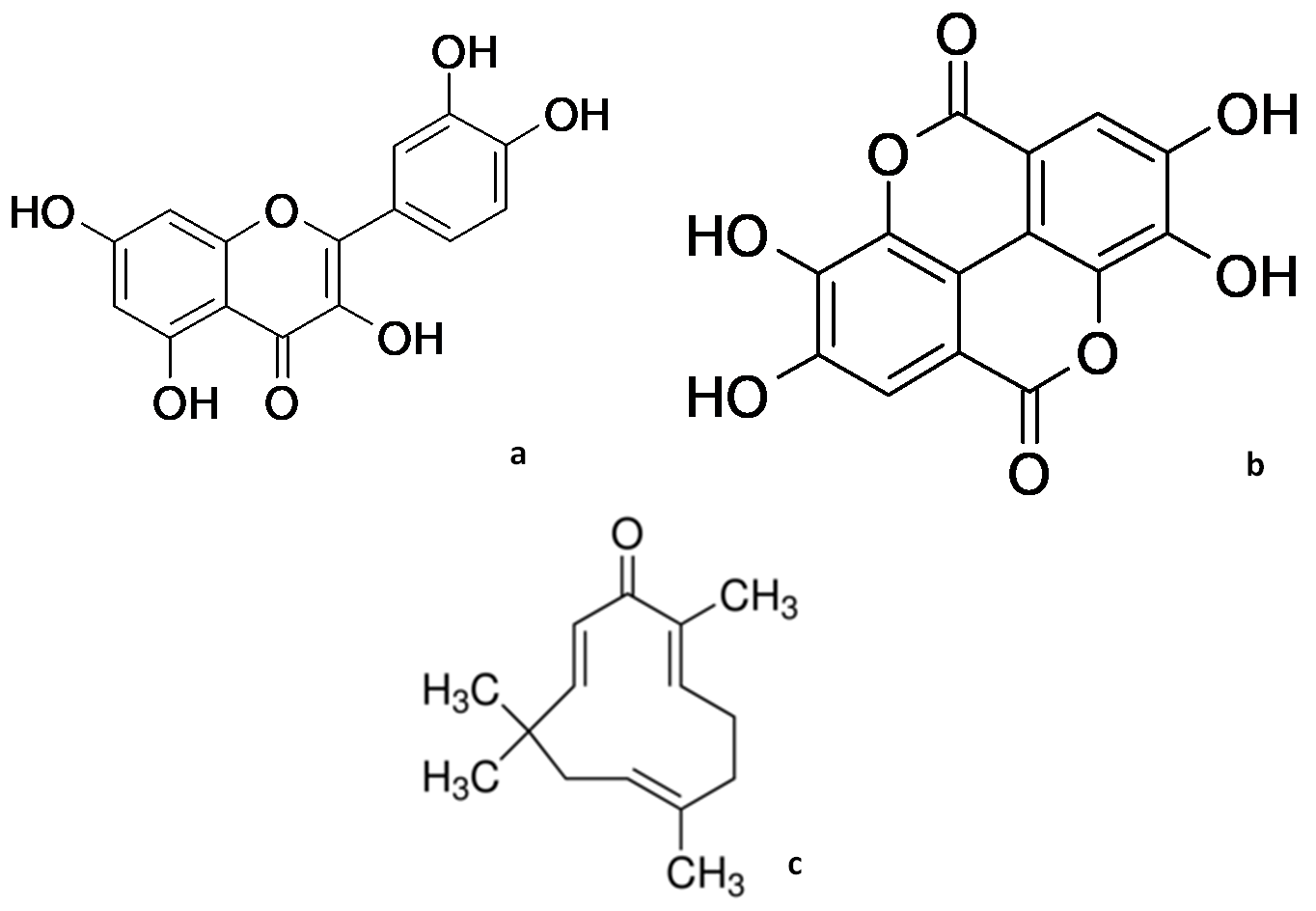


**Fig.S30.** 2D structures of (a) Quercetin, (b) Ellagic acid and (c) Zerumbone, extracted from fruits *of Averrhoa carambola* Linn., leave of *Clerodendrum colebrookianum* Walp., plants and rhizome of *Zingiber zerumbet* Roscoe respectively.

**Supplementary Information: Tables**

**Table S1.** Distinction between cyclooxygenase 1, 2 and 3 isoform.

| **Particulars** | **COX-1** | **COX-2** | **COX-3** | **References** |
| --- | --- | --- | --- | --- |
| m-RNA (kb) | 2.8, 5.1kb | 2.6-2.8, 4.6 kb | 2.9, 5.2 kb | [3, 21] |
| Gene length (kb) | ~ 22 kb | Gene ~ 8kb length | Variant | [22] |
| Signal peptide (amino acids) | 20-26 | 17 | 31 (additional aa) | [10] |
| TATA box | No | Yes | - | [22] |
| Hydrophobic header (amino acids) | 17 | 18 | Present | [22] |
| Intron | 10 | 9 | 10 (additional intron retain) | [3, 22] |
| Exons | 11 | 10 | 11 | [3, 22] |
| Sequence length (without and with signal peptide) | 576-599 | 581-604 | 629-630 | [8, 22] |
| Chromosomal location | 9 | 1 | 9 | [4, 21-22] |
| Mass (kDa) | Approx. 70 | Approx.70 | Approx. 72 | [7, 21] |
| Structure | Animal (1994) | Human (1996) | Homology Model in the present study | [23] |
| Nature | Constitutive | Inducible | - | [22] |
| RNA half life (hrs) | >12 | <3 | >12 | [9] |
| Glycosylation site/s | 3 | 2-4 | 4 | [24] |
| Catalysis (electron) | 2 e | 60 %- 2 e  40 % - 1e | - | [24] |
| Amino acids variation | ILE-523  ILE-434  HIS-513 | VAL-523  VAL-434  ARG-513 | ILE-523  ILE-434  HIS-513 | [24] |
| Active site | Small, less accommodating | Large, more accommodating | - | [24] |
| Allosterism (negative) | Present | Absent | Low concentration enough to produce an effect | [24] |
| Acetaminophen affinity | Poor | Poor | Good | [7, 25] |
| Blood-brain crossing | Poor | Poor | Good | [5, 6] |
| Signaling | Peripheral | Nuclear and G-protein Coupled Receptor | - | [24] |
| Substrate affinity | Poor | Good | Poor | [24] |
| Cofactor | Fe^++^ | Fe^++^ | Not required | [22] |

Table S2. Physicochemical and drug likeliness properties of the standard (selective and nonselective inhibitors) Cyclooxygenase enzyme calculated by open source tool DruLiTo [26], Marvin suite, and drug bank. Zerumbone has shown no pKa value due to the absence of ionizable atom.

| **Drug name** | **Molecular formula** | **No of rotation bond** | **No of Hydrogen Bond acceptor** | **No of hydrogen Bond Donors** | **Molecular weight** | **Pka** | **LogP** | **Polarizability** | **Refractivity** | **Polar surface area** | **LogS** |
| --- | --- | --- | --- | --- | --- | --- | --- | --- | --- | --- | --- |
| **Phenylbutazone^33^** | C_19_H_20_N_2_O_2_ | 5 | 2 | 0 | 308.3743 g/mol | 4.5 | 3.16 | 34.15 Å^3^ | 88.76 m3·mol^-1^ | 40.62 Å^2^ | -3.81 |
| **Sulindac^33^** | C_20_H_17_FO_3_S | 4 | 5 | 1 | 356.410583 g/mol | 4.7 | 3.42 | 37.2 Å3 | 99.56 m3·mol^-1^ | 73.6 A^2 | -4.2 |
| **Colchicine^33^** | C_22_H_25_NO_6_ | 5 | 6 | 1 | 399.437 g/mol | 1.85 | 1.03 | 42.41 Å3 | 111.38 m3·mol-1 | 83.09 Å^2^ | -4.2 |
| **Acetaminophen^33^** | C_8_H_9_NO_2_ | 1 | 2 | 2 | 151.16256 g/mol | 9.38 | 0.46 | 15.52 Å3 | 42.9 m^3^·mol^-1^ | 49.3 A^2 | -1.03 |
| **Benzoic acid^33^** | C_7_H_6_O_2_ |  | 2 | 1 | 122.12134 g/mol | 4.19 | 1.87 | 11.97 Å3 | 33.31 m3·mol-1 | 37.3 Å^2^ | -1.55 |
| **Bromfenacsodium^33^** | C_15_H_11_BrNNa_O_3 | 4 | 4 | 1 | 356.146509 g/mol | 3.81 | 3.4 | 29.93 Å3 | 80.26 m3·mol-1 | 83.2 A^^2^ | -4.4 |
| **Diclofenac sodium^33^** | C_14_H_10_C_l2_NNaO_2_ | 4 | 3 | 1 | 318.130469 g/mol | 4 | 4.98 | 27.93 Å3 | 75.46 m3·mol-1 | 52.2 A^^2^ | -4.8 |
| **Diflunisal^33^** | C_13_H_8_F_2_O_3_ | 2 | 5 | 2 | 250.197626 g/mol |  | 4.44 | 22.29 Å3 | 60.86 m3·mol-1 | 57.5 A^2 | -3.5 |
| **Etodolac^33^** | C_17_H_21_NO_3_ | 4 | 3 | 2 | 287.35354 g/mol | 4.65 | 2.5 | 31.94 Å3 | 81.16 m3·mol-1 | 62.3 A^2 | -3.9 |
| **Flurbiprofen^33^** | C_15_H_13_FO_2_ | 3 | 3 | 1 | 244.260923 g/mol | 4.42 | 4.16 | 25.23 Å3 | 67.29 m3·mol-1 | 37.3 A^2 | -4 |
| **Ibuprofen^33^** | C_13_H_18_O_2_ | 4 | 2 | 1 | 206.28082 g/mol | 4.91 | 3.97 | 23.76 Å3 | 60.73 m3·mol-1 | 37.3 A^2 | -3.99 |
| **Ketoprofen^33^** | C_16_H_14_O_3_ | 4 | 3 | 1 | 254.28056 g/mol | 3.88 | 3.29 | 26.56 Å3 | 72.52 m3·mol-1 | 54.37 Å2 | -4.1 |
| **Meclofenamate sodium^33^** | C_14_H_10_C_l2_NNaO_2_ | 3 | 3 | 2 | 318.130469 g/mol | 3.79 | 5 | 28.44 Å3 | 76.45 m3·mol-1 | 49.33 Å2 | -4.9 |
| **Nabumetone^33^** | C_15_H_16_O_2_ | 4 | 2 | 0 | 228.28634 g/mol | 19.59 | 3.08 | 26.17 Å3 | 68.43 m3·mol-1 | 26.3 Å2 | -5.1 |
| **Naproxen^33^** | C_14_H_14_O_3_ | 3 | 3 | 1 | 230.25916 g/mol | 4.15 | 3.18 | 24.81 Å3 | 64.85 m3·mol-1 | 46.5 A^2 | -4.16 |
| **Oxyphenbutazone^33^** | C_19_H_20_N_2_O_3_ | 5 | 3 | 1 | 324.3737 g/mol | 4.87 | 2.79 | 35.14 Å3 | 90.74 m3·mol-1 | 60.8 A^2 |  |
| **Piroxicam^33^** | C_15_H_13_N_3_O_4_S | 2 | 6 | 2 | 331.34642 g/mol | 6.3 |  | 32.27 Å3 | 87.04 m3·mol-1 | 108 A^2 | -4.16 |
| **Allopurinol^33^** | C_5_H_4_N_4_O | 0 | 2 | 2 | 136.11146 g/mol | 7.83 | -0.55 | 11.67 Å3 | 54.24 m3·mol-1 | 65.85 Å2 | -1.4 |
| **Sulfinpyrazone^33^** | C_23_H_20_N_2_O_3_S | 6 | 3 | 0 |  | 3.25 | 2.30 | 42.15 Å3 | 113.62 m3·mol-1 | 57.69 Å2 | -3.1 |
| **Indomethacin^33^** | C_19_H_16_ClN | 4 | 4 | 1 | 357.78764 g/mol | 4.5 | 4.27 | 36.64 Å3 | 94.81 m3·mol-1 | 68.53 Å2 | -4.62 |
| **Ellagic acid ^33^** | C_14_H_6_O_8_ | 0 | 6 | 4 | 302.1926 g/mol | 5.54 | 1.59 | 26.34 Å^3^ | 70.61 m^3^·mol^-1^ | 133.52 Å^2^ | -2.6 |
| **Zerumbone^34,35^** | C_15_H_22_O | 0 | 1 | 0 | 218.34 g/mol | - | 4.143 | 27.02 Å^3^ | 72.49 m^3^·mol^-1^ | 17.07 Å^2^ |  |
| **Quercetin ^33^** | C_15_H_10_O_7_ | 1 | 7 | 5 | 302.2357 g/mol | 6.44 | 1.81 | 28.54 Å^3^ | 76.86 m^3^·mol^-1^ | 127.45 Å^2^ | -3.1 |

**Table S3**. Various amino acid residue surrounding and potential bond interaction between a ligand (Quercetin, Ellagic acid, Zerumbone) and receptor (IL-10, TNF- α, COX-3). Important residues interaction essential for the functionis observed as mentioned in Table 1.

| **Receptor** | **Interleukin-10** | | | **TNF-α** | | | **COX-3** | | |
| --- | --- | --- | --- | --- | --- | --- | --- | --- | --- |
| Bond type/ ligand | **QCT** | **ELA** | **ZBN** | **QCT** | **ELA** | **ZBN** | **QCT** | **ELA** | **ZBN** |
| Charged (positive) | LYS-194 | LYS-194 | LYS-194 | **-** | **-** | **-** | **-** | ARG-150 | ARG -150 |
| Charged (negative) | GLU-14  GLU-101 | GLU -14  GLU -16  GLU -101 | GLU -14  GLU -101 | - | - | - | - | - | - |
| Polar | ASN-94  THR-93  THR-95  THR-103 | THR-103 | THR-95  THR-103 | GLN-60 (B)  SER-60 (B)  GLN-61 (A) | SER-60 (A)  GLN-61(B) | SER-60(A,B)  GLN-61(B) | HIS-120  SER-383  SER-560 | SER-383  SER-560 | SER-383  SER-560 |
| Hydrophobic | TRP-12  PHE-13  PHE-97 | PHE-13  ALA-15  PHE-97  VAL-102  MET-196 | TRP-12  PHE-13  ALA-15  VAL-102 | TYR-59(A,B)  TYR-119 (A,B)  LEU-120(A,B)  TYR-151(A,B) | TYR -59(A,B)  LEU -57(A,B)  TYR-119(A,B)  LEU-120(A)  TYR-151  ILE-155(B) | LEU-57(A,B)  TYR-59(A,B)  TYR-119(A,B)  LEU-120(A,B)  TYR-151(B)  ILE-155(A) | TYR-378  VAL-379  LEU-382  TYR-385  PHE-411  ILE-412  TYR-415  TRP-417  PHE-548  MET-552  ILE-553  ALA-557  LEU-561 | LEU-123  VAL-146  LEU-382  TYR-385  TYR-378  VAL-379  PHE-411  TYR-415  TRP-417  PHE-548  MET-552  ILE-553  ALA-557  LEU-561 | VAL-146  VAL-379  LEU-382  TYR-385  PHE-548  MET-552  ILE-553  LEU-389  ALA-557  LEU-561 |
| Glycine | - | - | - | GLY-121(A,B) | GLY-121(A)  GLY-122(A) | GLY-121(A,B)  GLY-122 | GLY-556 | GLY-556 | - |
| H-bond backbone | GLU-14  THR-95  GLU-101  LYS-194 | ALA-15  GLU-101 (2)  THR-103 | ALA-15  THR-103 | GLN-61  TYR-151 | TYR-151 | - | MET-552 | TYR-385  SER-560 | - |
| π –cation | - | LYS-194 (2) | - | - | - | - | - | - | - |
| π – π stacking | - | - | - | TYR-119(A,B) | - | - | TYR-385  TYR-415  TYR-417 | - | - |

**Table S4**. Specific hydrogen bond interaction between IL-10, TNF-α, COX-3 with Quercetin, Ellagic acid,and Zerumbone respectively. The residues in bracket *viz*. [π-cation] and {π-π- stacking} represented other bond interactions.

| Ligand  Target | **Virtual Screening** | | |
| --- | --- | --- | --- |
|  | **Quercetin** | **Ellagic Acid** | **Zerumbone** |
| IL-10 | **GLU-14**  **THR-95**  **GLU-101**  **LYS-194** | **ALA-15**  **GLU-101 (2)**  **THR-103**  [LYS-194 (2)] | **ALA-15**  **THR-103** |
| TNF-α | **GLN-61**  **TYR-151**  **SER-60 (B)**  **TYR-151 (B)**  {TYR-119 (A), (B)} | **TYR-151 (2)** | **NA** |
| COX-3 | **MET-552**  {TYR-385, TRP-417, TYR-415} | **SER-560**  **TYR-385** | **NA** |
| COX-1 | **TYR-354**  **ARG-119**  **SER-529** | **NA** | **NA** |

**Table S5.** Bond interaction table shows the specific role of representative amino acid residues that likely to participate at the active site during cyclooxygenase-ligand docking.

| S/N | Residue/  Position | Description/function |
| --- | --- | --- |
| 1 | ARG 120 | Double ionic bond. Important for NSAIDs containing carboxylates. Substrate orientation. GLU-524 forms salt bridge with ARG-120. Intermolecular interaction [1, 10,27-29]. |
| 2 | HIS 207 | Haem binding. Coordination with Fe^++^ bond [27]. |
| 3 | TYR-348 | Hydrogen bond abstraction (C-13 of arachidonate conformation) [29]. |
| 4 | VAL-349 | Hydrogen bond abstraction (C-13 of arachidonate conformation) [29]. |
| 5 | TYR-355 | Another ionic residue. Intermolecular interaction with HIS-190 [10, 27]. |
| 6 | TYR-385 | Forms reactive tyrosyl residue. Abstracts hydrogen bond and undergoes cyclization and/or oxygenation reaction. Redox reaction. Rate limiting step of cyclooxygenase catalysis [1, 5, 10, 27, 28]. |
| 7 | TRP-387 | Hydrophobic interaction. Hydrogen bond abstraction. Efficient endoperoxidase formation through stabilizing interaction [29]. |
| 8 | HIS-388 | Haem binding. Coordination with Fe^++^ bond [27]. |
| 9 | ALA-410 | Proper folding [28]. |
| 10 | ILE-523 | Differential binding mode by stearic hindrance (of oxygen attack) [10, 28]. |
| 11 | GLN-524 | Another ionic residue. Hydrogen bond network, salt bridge with HIS-513 [10, 27-28]. |
| 12 | SER-530 | Correct orientation, alternative posing; pushed to the top of the binding pocket. Hydrophobic pocket. Covalent modification. Not involved in catalysis. Acetylated by aspirin [1, 5, 10, 27-28]. |
| 13 | LEU-534 | Hydrophobic interaction. Hydrogen bond abstraction. A stearic hindrance that blocks premature oxygenation [29]. |
| 14 | Hydrophobic residues at binding site  LEU-117, ARG-120, PHE-205,PHE-209, VAL-344, ILE-348, VAL-349, LEU-352, SER-353, TYR-355,LEU-359, PHE-381, LEU-384, TYR-385, TRP-387, PHE-518, ILE-523, GLY-526, ALA-527, SER-530, LEU-531, GLY-533, LEU-534 | Active site residues that favour binding with ligands [1] |

**Supplementary Information: Scatter plots**

**RS-1: scatter plot COX3**

df <- read.table(file= "clipboard", sep = "\t", header=TRUE)

x <- df[,1];

y1 <- df[,2];

y2 <- df[,3];

y3 <- df[,4];

plot(x,y1, type ='scatter', col = 'black',xlab = 'Time(ps)', ylab='C-Alpha(RMSD)', ylim = range(0:4));

lines(x,y2, type='scatter', col = 'red');

lines(x,y3, type='scatter', col = 'green');

lnames<- c('COX3_Quercetin', 'COX3_Ellagic Acid', 'COX3_Zerumbone');

legend('bottomright', lnames, col = 1:3, lty = 1,cex=0.75, lwd= 1, y.intersp=0.75,x.intersp=0.5);

**RS-2: scatter plot TNFα**

df <- read.table(file= "clipboard", sep = "\t", header=TRUE)

x <- df[,1];

y1 <- df[,2];

y2 <- df[,3];

y3 <- df[,4];

plot(x,y1, type ='scatter', col = 'black',xlab = 'Time(ps)',ylab = 'C-Alpha(RMSD)', ylim = range(0:3));

lines(x,y2, type='scatter', col = 'red');

lines(x,y3, type='scatter', col = 'green');

lnames<- c('TNFa_Quercetin', 'TNFa_Ellagic_Acid', 'TNFa_Zerumbone');

legend('bottomright', lnames, col = 1:3, lty = 1,cex=0.75, lwd= 1, y.intersp=0.75,x.intersp=0.5);

**RS-3: scatter plot IL10**

df <- read.table(file= "clipboard", sep = "\t", header=TRUE)

x <- df[,1];

y1 <- df[,2];

y2 <- df[,3];

y3 <- df[,4];

plot(x,y1, type ='scatter', col = 'black',xlab = 'Time(ps)', ylab='C-Alpha(RMSD)', ylim = range(0:4));

lines(x,y2, type='scatter', col = 'red');

lines(x,y3, type='scatter', col = 'green');

lnames<- c('IL10_Quercetin', 'IL10_Ellagic_Acid', 'IL10_Zerumbone');

legend('bottomright', lnames, col = 1:3, lty = 1,cex=0.75, lwd= 1, y.intersp=0.75,x.intersp=0.5);

**RS-4: scatter plot COX1**

df <- read.table(file= "clipboard", sep = "\t", header=TRUE)

x <- df[,1];

y1 <- df[,2];

y2 <- df[,3];

y3 <- df[,4];

plot(x,y1, type ='scatter', col = 'black',xlab = 'Time(ps)', ylab='C-Alpha(RMSD)', ylim = range(0:6));

lines(x,y2, type='scatter', col = 'red');

lines(x,y3, type='scatter', col = 'green');

lnames<- c('COX1_Quercetin', 'COX1_Ellagic_Acid', 'COX1_Zerumbone');

legend('bottomright', lnames, col = 1:3, lty = 1,cex=0.75, lwd= 1, y.intersp=0.75,x.intersp=0.5);

**References**

1. Thuresson, E.D. *et al*. Prostaglandin Endoperoxide H synthase-1 the functions of cyclooxygenase active site residues in the binding, positioning, and oxygenation of arachidonic acid. *Journal of Biological Chemistry* **276(13)**, 10347-57 (2001).
2. Li S, Dou W, Tang Y, Goorha S, Ballou LR, Blatteis CM. Acetaminophen: antipyretic or hypothermic in mice? In either case, PGHS-1b (COX-3) is irrelevant. *Prostaglandins & Other Lipid Mediators* **85(3-4),** 89-99 2008.
3. Chandrasekharan NV, Dai H, Roos KLT, Evanson NK, Tomsik J, Elton TS, Simmons DL. COX-3, a cyclooxygenase-1 variant inhibited by acetaminophen and other analgesic/antipyretic drugs: Cloning, structure, and expression. PNAS 2002; 99(21): 13926-31.
4. Qin N, Codd E, Flores C, Zhang SP. Human cyclooxygenase-3 enzyme and uses thereof. Washington, DC: U.S. Patent and Trademark Office. U.S. Patent 2004; Application. No. 10/783297.
5. Simmons D, Chandrasekharan NV. Cyclooxygenase variants and methods of use. Washington, DC: U.S. Patent and Trademark Office. U.S. Patent. 2007; No.: 7179627.
6. Simmons D, Chandrasekharan NV.  Antibodies to cyclooxygenase variants*.* Washington, DC: U.S. Patent and Trademark Office. U.S. Patent. 2009; No.: 7601816.
7. Simmons DL, Lee JJ, Hunter JC, Logon G. Use of cox-3 binding molecules for modulating autophagy. U.S. Patent Application*,* 2015; No.: 14/505338.
8. Simmons DL, Botting RM, Hla T. Cyclooxygenase isozymes: the biology of prostaglandin synthesis and inhibition. Pharmacological reviews 2004; 56(3): 387-37.
9. Cui JG, Kuroda H, Chandrasekharan NV, Pelaez RP, Simmons DL, Bazan NG, Lukiw WJ. Cyclooxygenase-3 gene expression in Alzheimer hippocampus and in stressed human neural cells. Neurochemical research 2004; 29(9): 1731-37.
10. Filizola M, Perez JJ, Palomer A, Mauleon D. Comparative molecular modeling study of the three-dimensional structures of prostaglandin endoperoxide H2 synthase 1 and 2 (COX-1 and COX-2). Journal of Molecular Graphics and Modelling 1997; 15(5): 290-300.
11. Funk CD, Funk LB, Kennedy ME, Pong AS, Fitzgerald GA. Human platelet/erythroleukemia cell prostaglandin G/H synthase: cDNA cloning, expression, and gene chromosomal assignment. The FASEB Journal 1991; 5(9): 2304-12. (47)
12. Gupta S, Jadaun A, Kumar H, Raj U, Varadwaj PK, Rao AR. Exploration of new drug-like inhibitors for serine/threonine protein phosphatase 5 of Plasmodium falciparum: a docking and simulation study. Journal of Biomolecular Structure and Dynamics 2015; 33(11): 2421-41.
13. Gupta S, Singh Y, Kumar H, Raj U, Rao AR, Varadwaj PK. Identification of Novel Abiotic Stress Proteins in *Triticum aestivum* Through Functional Annotation of Hypothetical Proteins. Interdisciplinary Sciences: Computational Life Sciences 2016; 10(1): 205-20.
14. Oksuz E, Atalar F, Tanırverdi G, Bilir A, Shahzadi A, Yazici Z. Therapeutic potential of cyclooxygenase-3 inhibitors in the management of glioblastoma. Journal of neuro-oncology 2016; 126(2): 271-78.
15. Smith A. Analgesics: Time for a new COX?  Nature Reviews Drug Discovery 2002; 1(11): 839.
16. Altschul, SF, Gish, W, Miller, W, Myers, EW, & Lipman, DJ. Basic local alignment search tool. Journal of molecular biology 1990; 215(3): 403-410. Available from: [http://www.niper.gov.in/pi dev_tools/DruLiToWeb/DruLi ToContact us.html](http://www.niper.gov.in/pi%20dev_tools/DruLiToWeb/DruLi%20ToContact%20us.html).
17. Saitou N. and Nei M. The neighbor-joining method: A new method for reconstructing phylogenetic trees. Molecular Biology and Evolution 1987; 4: 406-425.
18. Felsenstein J. Confidence limits on phylogenies: An approach using the bootstrap. Evolution 1985; 39: 783-791.
19. Nei M. and Kumar S.  Molecular Evolution and Phylogenetics. Oxford University Press, New York; 2000.
20. Kumar S., Stecher G., Li M., Knyaz C., and Tamura K. MEGA X: Molecular Evolutionary Genetics Analysis across computing platforms. Molecular Biology and Evolution 2018; 35: 1547-1549.
21. Schwab JM, Schluesener HJ, Laufer S. COX-3: just another COX or the solitary elusive target of paracetamol? The Lancet 2003; 361: 981-82.
22. Chandrasekharan NV, Simmons DL. The cyclooxygenases. Genome biology 2004; 5(9): 241
23. Picot D, Loll PJ Garavito RM. The X-ray crystal structure of the membrane protein prostaglandin H2 synthase-1. Nature 1994; 367(6460): 243.
24. Smith WL, DeWitt DL Garavito RM. Cyclooxygenases: structural, cellular, and molecular biology. Annual review of biochemistry 2000; 69(1): 145-82.
25. Kis B, Snipes JA, Busija, DW. Acetaminophen and the cyclooxygenase-3 puzzle: sorting out facts, fictions, and uncertainties. Journal of Pharmacology and Experimental Therapeutics 2005; 315(1): 1-7.
26. DruLiTo: Drug likeliness Tool. Niper. Protocols 2015. Open-source tool
27. Takada Y, Murakami A, Aggarwal BB. Zerumbone abolishes NF-κB and IκBa kinase activation leading to suppression of antiapoptotic and metastatic gene expression, upregulation of apoptosis, and downregulation of invasion. Oncogene 2005; 24: 6957–69.
28. Muthu N, Lee SY, Phua KK, Bhore SJ. Nutritional, Medicinal and Toxicological Attributes of Star-Fruits (*Averrhoa carambola* L.): A Review. Bioinformation 2016; 12(12): 420-24.
29. Willoughby DA, Moore AR, Colville-Nash PR. COX-1, COX-2, and COX-3 and the future treatment of chronic inflammatory disease. The Lancet 2000; 355: 646-648.
30. Bijina B, Chellappan S, Krishna JG, Basheer SM, Elyas KK, Bahkali AH, Chandrasekaran M. Protease inhibitor from *Moringa oleifera* with potential for use as therapeutic drug and as seafood preservative. Saudi Journal of Biological Sciences 2011; 18: 273–281.
31. Das B, Choudhury MD, Dey A, Talukdar AD, Nongalleima K, Deb L. Antioxidant and Anti-Inflammatory Activity of Aqueous and Methanolic Extracts of Rhizome Part of *Drynaria quercifolia* (L.) J. Smith. Int J Pharm Pharm Sci 2014; 6(6): 43-49.

**Isolation of Quercetin type compound from hydro-alcoholic extract (50% ethanol) of** ***Averrhoa carambola* Linn. fruits**

In HPLC analysis, 8 different peaks have been observed after injection of 20μl of samples, where one peak of ethyl acetate fraction of *C. colebrookianum* aqueous extract (EtFCc) was nearly similar to signals of standard Quercetin. Accordingly, an isolation strategy was designed as per published literature. The isolated fractions have monitored by thin layer chromatography (TLC). In TLC method mobile phase used Butanol: Acetic acid: Water (13:3:5) and Iodine Chamber or UV light or H_2_SO_4_ used as detecting methods. The fraction shown similar Rf valued as standard Quercetin (Sigma, USA) has designated as sample LD-A (Fig-S31).


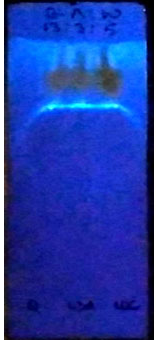


**Fig-S31**

**HPLC Analysis of the test samples**

The HPLC system - LC-2010 CHT & LC-2010AHT (Shimadzu Corporation, Kyoto, Japan) consisted of a UV-VIS detector and a reverse phase C18, (250 x 4.6 mm, 5 mm particle sizes) Column (Mark, Germany) was used. Running conditions included: injection volume, 10μl; mobile phase, HPLC grade Acetonitrile (Rankem, Delhi) and water (acidified with 0.01N of KH2PO4 (Rankem, Delhi), pH was adjusted to 2.8 using ortho-phosphoric acid). The flow rate has maintained 1.3 ml/min and elutes monitored at 370nm. The isolated compound (LD-A) from *Averrhoa carambola* fruits was dissolved in 100% HPLC grade methanol, vortex and filtered through an ultra-membrane filter (pore size 0.45 μm; E-Merck, Darmstadt, Germany) prior to injection in HPLC. The Standard Compound Quercetin (Sigma) was dissolved in 100% HPLC grade methanol (Rankem, Delhi) and filtered prior to inject in HPLC under similar condition. Chromatograms given below in fig. S32 & S33.

**NOTE:**

10 mg Sample dissolved in 10 ml methanol

1 mg standard dissolved in 1 ml methanol

**
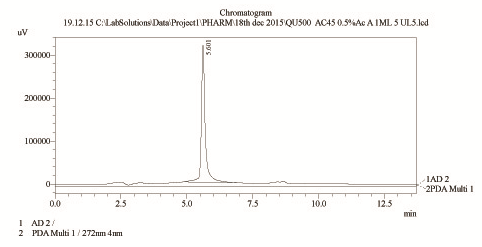
**

**Fig. S32:** Chromatogram of Standard Quercetin (Sigma)


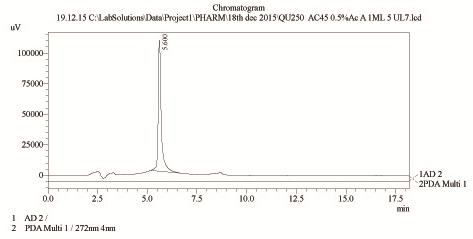


**Fig. S33:** Chromatogram of isolated compound (LD-A) from *Averrhoa carambola* fruits

**Isolation of Ellagic acid like compound from Ethyl acetate fraction of *Clerodendrum colebrookianum* Walp leaves**

The isolated fractions similarly were monitored by thin layer chromatography (TLC) and fraction shown similar Rf valued as standard Ellagic acid has designated as sample LD-B (Fig. S34).


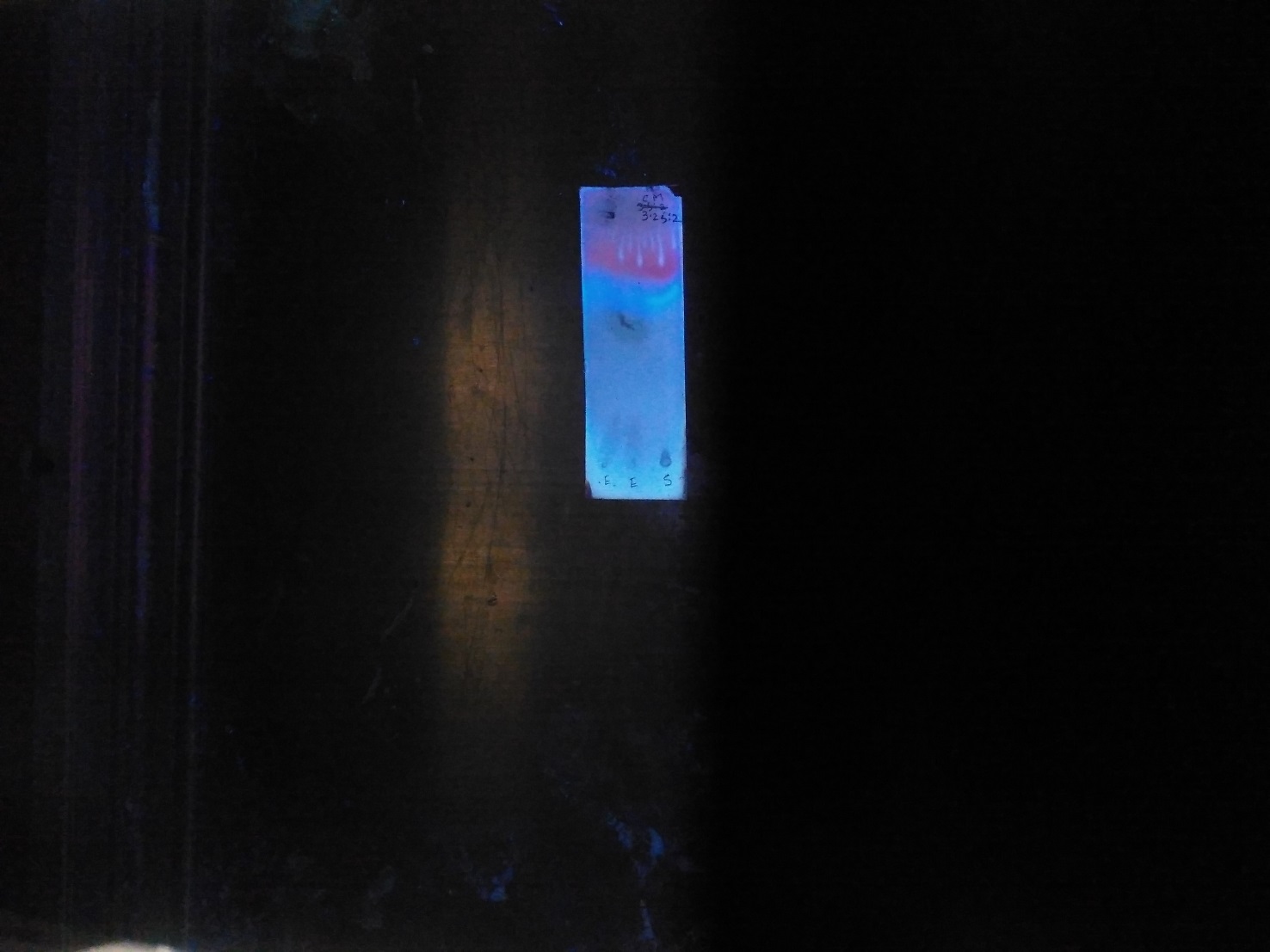


**Fig.-S34**

**HPLC Analysis of the test samples**

The HPLC system - LC-2010 CHT & LC-2010AHT (Shimadzu Corporation, Kyoto, Japan) consisted of a UV-VIS detector and a reverse phase C18, (250 x 4.6 mm, 5 mm particle sizes) Column (Mark, Germany) was used. Running conditions included: injection volume, 10μl; mobile phase, HPLC grade Acetonitrile (Rankem, Delhi) and water (acidified with 0.01N of KH2PO4 (Rankem, Delhi), pH was adjusted to 2.8 using ortho-phosphoric acid). The flow rate has maintained 1.3 ml/min and elutes monitored at 370nm. The isolated compound (LD-B) from ethyl acetate fraction of *C. colebrookianum* leaves was dissolved in 100% HPLC grade methanol, vortex and filtered through an ultra-membrane filter (pore size 0.45 μm; E-Merck, Darmstadt, Germany) prior to injection in HPLC. The Standard compounds i.c. Ellagic acid (Sigma) was dissolved in 100% HPLC grade methanol (Rankem, Delhi) and filtered prior to inject in HPLC under similar condition. Chromatograms given below in fig S35 & S36.

**NOTE:**

10 mg Sample dissolved in 10 ml methanol

1 mg standard dissolved in 1 ml methanol

**
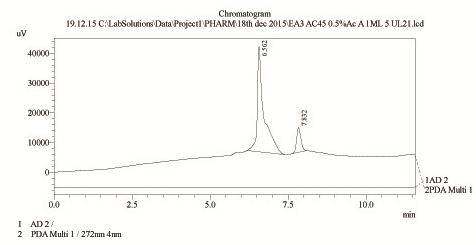
**

**Fig. S35:** Chromatogram of Standard Ellagic acid (Sigma)


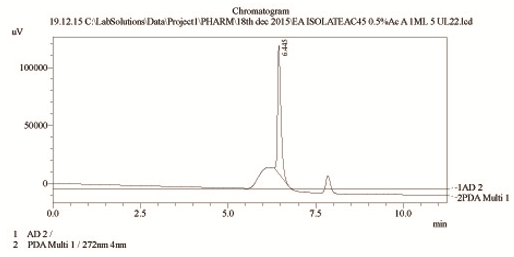


**Fig. S36:** Chromatogram of isolated compound (LD-B) from ethyl acetate fraction of *C. colebrookianum* leaves

**Zerumbone (ZBN) like compound isolated from *Zingiber zerumbet* Roscoe (ZzR) Rhizome**

The dried powder of ZzR was extracted in a Soxhlet extractor with ethanol (95%) and isolated ZBN as per the method described by Huang et al., 2005. The presence of ZBN was confirmed in the isolates by comparing them with commercial ZBN (Sigma) through high-performance liquid chromatography (HPLC), and further purification and identification were performed.

**HPLC Analysis of the test samples**

The HPLC system consisted of a Waters 600 E pump, A Waters 2996 photodiode array detector and an inline Waters AF-degasser. A 20 µl injection volume was used in all analysis. The chromatographic separation was performed using symmetry RP_18_ (250X4 mm, 5µm particle size, HPLC column (Merck, KgGA) at 25^0^C. A mixture of solvent (A) 60 % acetonitrile and solvent (B) 40 % water was used as a mobile phase to separate the compound at a flow rate of 0.4 ml/min. The mobile phase was filtered through a 0.45 µm membrane filters (Waters, USA). Samples of HPLC were filtered through a 0.22 µm membrane filters. Chromatograms given below in fig. S37 & S38.

254 nm detector.

**Fig. – S37:** Chromatogram of isolated compound from ethanol (95%) extract of *Zingiber zerumbet* Roscoe (ZzR) Rhizome

**Fig. – S38:** Chromatogram of Standard Zerumbone compound (Sigma, USA)

**Western blotting original images**

**COX-3 -A**


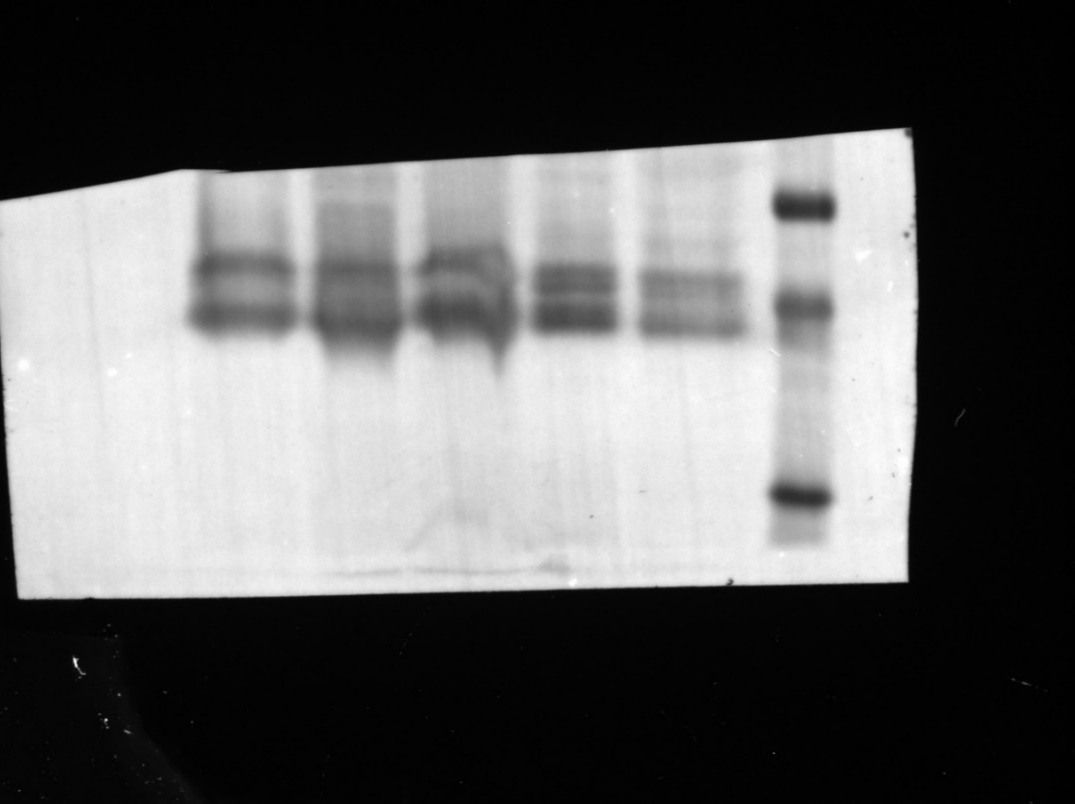


COX-3

**NFκB – B**


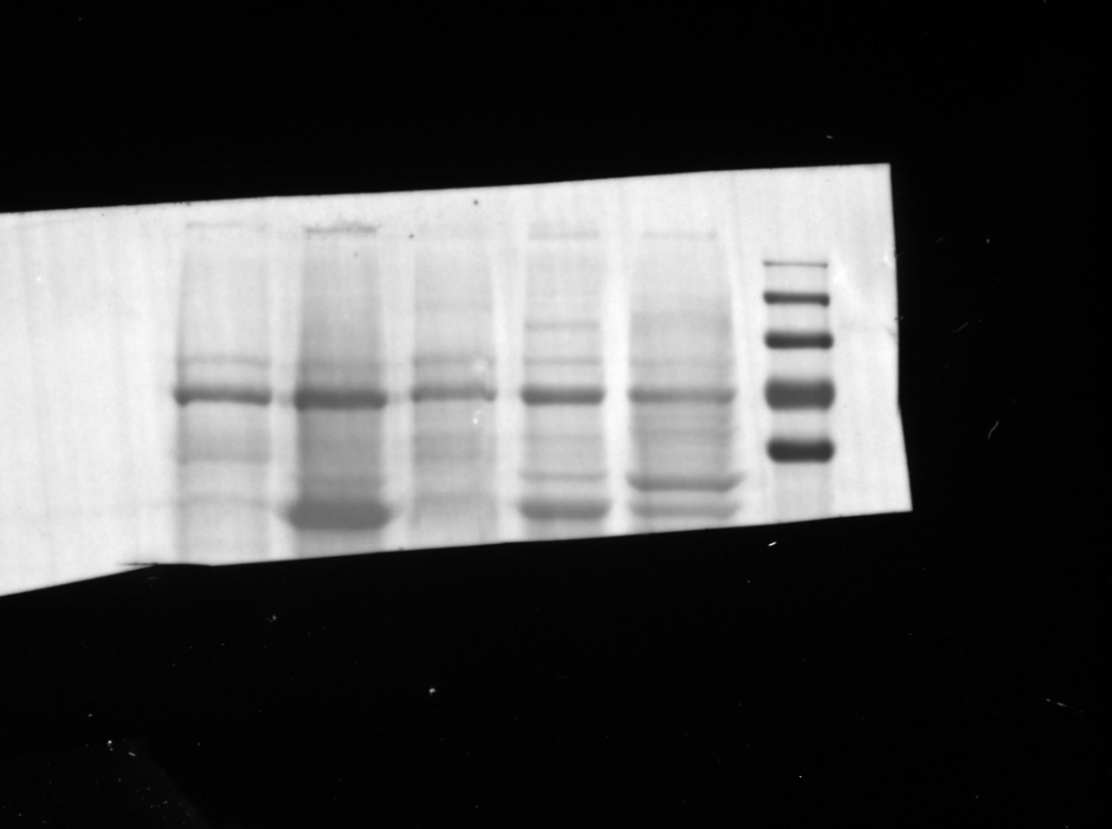


NF*K*B

**ß-actin**


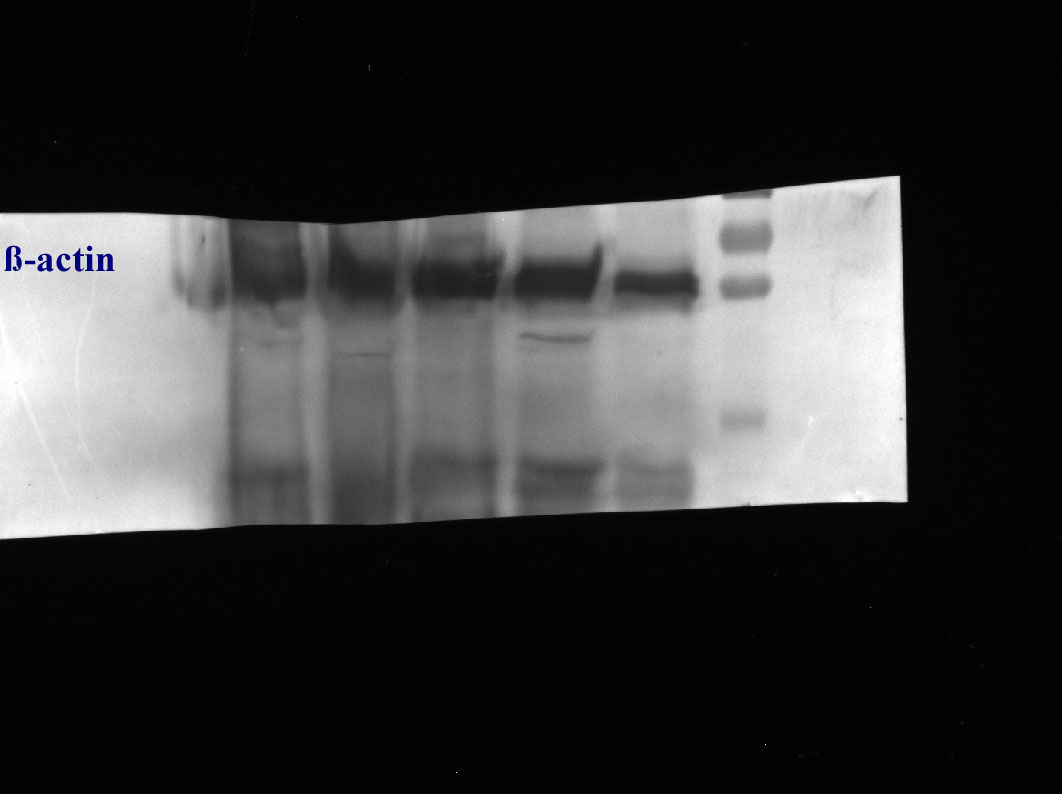


NFKB cropped Marker


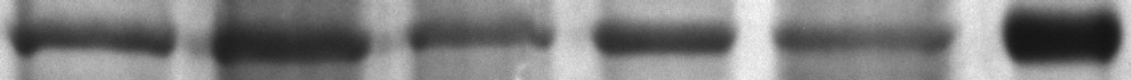


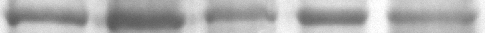


COX-3 cropped Marker


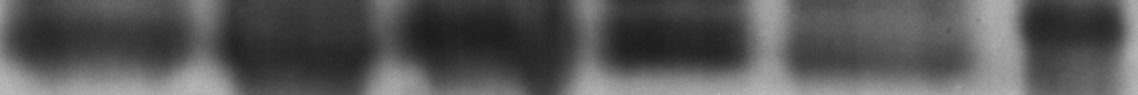


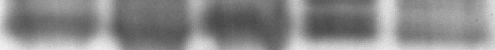


b-actin cropped Marker


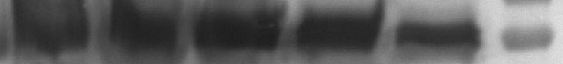


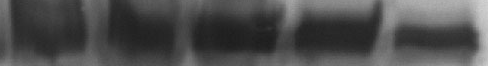


**Integrated Density value**

| **Treatment** | **beta actin** | **cox3** | **NfkB** | **COX-3** | **Mean ±**  **SEM** | **NFKB** | **Mean**  **±**  **SEM** |
| --- | --- | --- | --- | --- | --- | --- | --- |
|  | Beta-actin (BA) | COX-3 (C3) | NFKB (NF) | C3/BA (COX-3 IDV) |  | NF/BA (NFKB IDV) |  |
| Normal 1 | 92.76527263 | 32.895761 | 40.9647327 | 0.354612883 | 0.3537456407  **±**  0.0008589 | 0.441595561 | 0.4408991403  **±**  0.0007039 |
| Normal 2 | 93.12435241 | 33.021547 | 41.1246851 | 0.354596259 |  | 0.441610428 |  |
| Normal 3 | 92.45362111 | 32.546243 | 40.6325743 | 0.352027780 |  | 0.439491432 |  |
| n1 AC 1 | 93.34734252 | 65.644079 | 61.4172411 | 0.703223864 | 0.702636234  **±**  0.004453 ***C | 0.657943113 | 0.6553403703  **±**  0.002480 ***C |
| n1 AC 2 | 93.75328512 | 65.125346 | 60.9754623 | 0.694646016 |  | 0.650382141 |  |
| n1 AC 3 | 92.98254122 | 66.021214 | 61.1542321 | 0.710038822 |  | 0.657695857 |  |
| n2 ZBN 1 | 96.16278582 | 56.297371 | 45.2904027 | 0.585438230 | 0.5860606703  **±**  0.002605 *** | 0.470976400 | 0.4709583963  **±**  0.001381*** |
| n2 ZBN 2 | 95.87652143 | 56.648732 | 44.9236542 | 0.590850932 |  | 0.468557406 |  |
| n2 ZBN 3 | 96.21452146 | 55.986542 | 45.5423147 | 0.581892849 |  | 0.473341383 |  |
| n3 ELA 1 | 89.53034842 | 35.023204 | 59.0123143 | 0.391188062 | 0.3943430537  **±**  0.002477 *** | 0.659131963 | 0.663570071  **±**  0.002821 |
| n3 ELA 2 | 88.83214542 | 34.876543 | 58.8754621 | 0.392611738 |  | 0.662772038 |  |
| n3 ELA 3 | 89.01254387 | 35.536421 | 59.5321423 | 0.399229361 |  | 0.668806212 |  |
| n5 QCT 1 | 95.39399253 | 53.276067 | 66.2314785 | 0.558484513 | 0.5587662603  **±**  0.0009843 *** | 0.694294019 | 0.6921550227  **±**  0.003480*** |
| n5 QCT 2 | 94.87356214 | 52.865432 | 65.0215453 | 0.557219849 |  | 0.685349468 |  |
| n5 QCT 3 | 95.65478924 | 53.623541 | 66.6543215 | 0.560594419 |  | 0.696821581 |  |

**Raw Original data**

**In-vivo anti-arthritic activity (Rat Hind Paw Edema and pain Intensity)**

| **Treatment** | **Hind Paw Edema of rats** | | | | | **PAM (force in gf of knee joint)** | | | |
| --- | --- | --- | --- | --- | --- | --- | --- | --- | --- |
|  | Normal | 0 hour | 1^st^ hour | 7^th^ day | 14^th^ day | Normal | 1 hour | 7^th^ day | 14^th^ day |
| **Control**  (water 5ml/kg+0.1mL normal saline) | 0.28  ±  0.02 | 0.36  ±  0.01 | 0.31  ±  0.01 | 0.29  ±  0.02 | 0.29  ±  0.02 | 222.32  ±  9.92 | 222.16  ±  11.52 | 228.02  ±  10.43 | 229.98  ±  10.57 |
| **Arthritic Control**  (water 5ml/kg + 0.1mL Complete Freund’s adjuvant-induced arthritis) | 0.25  ±  0.014 | 0.32  ±  0.004 | 0.34  ±  0.004 | 0.42  ±  0.005  *****c** | 0.45  ±  0.011  *****c** | 205.46  ±  9.23 | 195.38  ±  10.32 | 63.46  ±  3.014  *****c** | 55.02  ±  3.559  *****c** |
| **Standard**  (50mg/kg/day Diclofenac sodium + 0.1mL Complete Freund’s adjuvant-induced arthritis) | 0.25  ±  0.010 | 0.40  ±  0.014 | 0.42  ±  0.010 | 0.43  ±  0.007 | 0.38  ±  0.011  ***** | 251.4  ±  7.868 | 210.85  ±  12.683 | 174.45  ±  9.851  ******* | 180.85  ±  8.672  ******* |
| **Test-1**  (50mg/kg/day Ellagic acid + 0.1mL Complete Freund’s adjuvant-induced arthritis) | 0.31  ±  0.012 | 0.41  ±  0.011 | 0.43  ±  0.012 | 0.37  ±  0.027 | 0.30  ±  0.011  ******* | 217.46  ±  11.102 | 219.38  ±  9.639 | 141.08  ±  11.782  ******* | 147  ±  11.011  ******* |
| **Test-2**  (50mg/kg Querceitin + 0.1mL Complete Freund’s adjuvant-induced arthritis) | 0.28  ±  0.021 | 0.38  ±  0.014 | 0.38  ±  0.012 | 0.42  ±  0.011 | 0.36  ±  0.011  ****** | 197.22  ±  19.157 | 198.62  ±  19.143 | 59.82  ±  5.501 | 62.04  ±  5.354 |
| **Test-3**  (50mg/kg Zerumbone + 0.1mL Complete Freund’s adjuvant-induced arthritis) | 0.30  ±  0.019 | 0.38  ±  0.022 | 0.39  ±  0.023 | 0.44  ±  0.016 | 0.34  ±  0.013  ******* | 189.38  ±  22.90 | 191.22  ±  22.62 | 117.22  ±  13.06  ****** | 122.22  ±  13.12  ******* |

Averages values of raw data were expressed as a Mean ± SEM, n=5, For numerical results, one-way analyses of variance (ANOVA) with Tukey-Kramer Multiple Comparisons posttests were performed using GraphPad InStat Version 3 (GraphPad Software). The minimum value of *p*<0.05 was considered as significant. *^C^*p*<0.05*,***^C^*p*<0.01, ***^C^*p***<**0.001 compared to Control group; **p*<0.05, ***p*<0.01, ****p*<0.001 compared to Arthritic control group.

**In-vivo anti-arthritic activity (Paw Edema and pain Intensity)**

| **Sl. No.** | **Treatment** | **Hind Paw Edema of rats** | | | | | **PAM (force in gf of hind paw)** | | | |
| --- | --- | --- | --- | --- | --- | --- | --- | --- | --- | --- |
|  |  | Normal | 0 hour | 1 hour | 7^th^ day | 14^th^ day | Normal | 1 hour | 7^th^ day | 14^th^ day |
|  | **Control**  (water 5ml/kg+0.1mLnormal saline) | 0.23 | 0.33 | 0.27 | 0.24 | 0.24 | 235.3 | 239.5 | 240.3 | 243.5 |
|  |  | 0.33 | 0.39 | 0.35 | 0.33 | 0.34 | 243.5 | 245.3 | 250.5 | 250.3 |
|  |  | 0.29 | 0.35 | 0.30 | 0.29 | 0.30 | 189.7 | 185.3 | 193.3 | 195.5 |
|  |  | 0.32 | 0.39 | 0.33 | 0.32 | 0.32 | 233.5 | 235.4 | 240.5 | 245.3 |
|  |  | 0.25 | 0.33 | 0.29 | 0.25 | 0.27 | 209.6 | 205.3 | 215.5 | 215.3 |
| **Mean ± SEM** | | 0.28  ±  0.02 | 0.36  ±  0.01 | 0.31  ±  0.01 | 0.29  ±  0.02 | 0.29  ±  0.02 | 222.32  ±  9.92 | 222.16  ±  11.52 | 228.02  ±  10.43 | 229.98  ±  10.57 |
|  | **Arthritic Control**  (water 5ml/kg + 0.1mL Complete Freund’s adjuvant-induced arthritis) | 0.24 | 0.33 | 0.34 | 0.42 | 0.43 | 233.5 | 227.3 | 73.5 | 67.5 |
|  |  | 0.23 | 0.31 | 0.33 | 0.41 | 0.45 | 215.3 | 205.5 | 65.3 | 57.3 |
|  |  | 0.29 | 0.33 | 0.35 | 0.42 | 0.43 | 193.3 | 185.5 | 59.3 | 49.5 |
|  |  | 0.28 | 0.32 | 0.34 | 0.43 | 0.45 | 179.5 | 165.3 | 55.7 | 47.3 |
|  |  | 0.22 | 0.31 | 0.35 | 0.44 | 0.49 | 205.7 | 193.3 | 63.5 | 53.5 |
| **Mean ± SEM** | | 0.25  ±  0.014 | 0.32  ±  0.004 | 0.34  ±  0.004 | 0.42  ±  0.005 | 0.45  ±  0.011 | 205.46  ±  9.23 | 195.38  ±  10.32 | 63.46  ±  3.014 | 55.02  ±  3.559 |
|  | **Standard**  (50mg/kg/day Diclofenac sodium + 0.1mL Complete Freund’s adjuvant-induced arthritis) | 0.28 | 0.45 | 0.45 | 0.45 | 0.39 | 232.4 | 225.3 | 145.3 | 187.9 |
|  |  | 0.27 | 0.42 | 0.43 | 0.44 | 0.40 | 267.5 | 207.3 | 185.5 | 193.3 |
|  |  | 0.24 | 0.40 | 0.40 | 0.42 | 0.34 | 257.5 | 247.3 | 187.3 | 189.5 |
|  |  | 0.22 | 0.38 | 0.40 | 0.41 | 0.39 | 230.3 | 189.5 | 145.3 | 155.3 |
|  |  | 0.25 | 0.37 | 0.41 | 0.44 | 0.36 | 250.3 | 199.3 | 179.7 | 185.3 |
| **Mean ± SEM** | | 0.25  ±  0.010 | 0.40  ±  0.014 | 0.42  ±  0.010 | 0.43  ±  0.007 | 0.38  ±  0.011 | 251.4  ±  7.868 | 210.85  ±  12.683 | 174.45  ±  9.851 | 180.85  ±  8.672 |
|  | **Test-1**  (50mg/kg/day Ellagic acid + 0.1mL Complete Freund’s adjuvant-induced arthritis) | 0.27 | 0.44 | 0.46 | 0.47 | 0.31 | 243.5 | 240.3 | 173.5 | 185.3 |
|  |  | 0.34 | 0.43 | 0.45 | 0.37 | 0.32 | 225.3 | 227.3 | 145.3 | 150.5 |
|  |  | 0.33 | 0.41 | 0.43 | 0.35 | 0.33 | 197.5 | 203.5 | 125.7 | 125.5 |
|  |  | 0.32 | 0.40 | 0.40 | 0.32 | 0.29 | 235.3 | 235.5 | 155.4 | 150.7 |
|  |  | 0.31 | 0.38 | 0.40 | 0.33 | 0.27 | 185.7 | 190.3 | 105.5 | 125.3 |
| **Mean ± SEM** | | 0.31  ±  0.012 | 0.41  ±  0.011 | 0.43  ±  0.012 | 0.37  ±  0.027 | 0.30  ±  0.011 | 217.46  ±  11.102 | 219.38  ±  9.639 | 141.08  ±  11.782 | 147  ±  11.011 |
| **Sl. No.** | **Treatment** | **Hind Paw Edema** | | | | | **PAM data** | | | |
|  |  | Normal | 0 hour | 1 hour | 7^th^ day | 14^th^ day | Normal | 1 hour | 7^th^ day | 14^th^ day |
|  | **Test-2**  (50mg/kg Querceitin + 0.1mL Complete Freund’s adjuvant-induced arthritis) | 0.22 | 0.37 | 0.36 | 0.41 | 0.37 | 245.5 | 247.3 | 75.3 | 75.5 |
|  |  | 0.33 | 0.41 | 0.40 | 0.43 | 0.34 | 183.5 | 183.3 | 53.5 | 55.6 |
|  |  | 0.25 | 0.33 | 0.35 | 0.38 | 0.35 | 225.7 | 225.7 | 67.5 | 70.3 |
|  |  | 0.30 | 0.39 | 0.41 | 0.43 | 0.33 | 197.5 | 201.3 | 59.3 | 63.5 |
|  |  | 0.30 | 0.39 | 0.40 | 0.43 | 0.39 | 133.9 | 135.5 | 43.5 | 45.3 |
| **Mean ± SEM** | | 0.28  ±  0.021 | 0.38  ±  0.014 | 0.38  ±  0.012 | 0.42  ±  0.011 | 0.36  ±  0.011 | 197.22  ±  19.157 | 198.62  ±  19.143 | 59.82  ±  5.501 | 62.04  ±  5.354 |
|  | **Test-3**  (50mg/kg Zerumbone + 0.1mL Complete Freund’s adjuvant-induced arthritis) | 0.24 | 0.32 | 0.33 | 0.44 | 0.34 | 235.3 | 235.5 | 150.3 | 155.5 |
|  |  | 0.28 | 0.33 | 0.35 | 0.39 | 0.32 | 187.5 | 185.3 | 97.5 | 103.5 |
|  |  | 0.33 | 0.40 | 0.41 | 0.43 | 0.32 | 193.5 | 195.3 | 107.3 | 115.3 |
|  |  | 0.35 | 0.43 | 0.45 | 0.49 | 0.39 | 105.3 | 109.5 | 85.3 | 87.5 |
|  |  | 0.31 | 0.40 | 0.43 | 0.45 | 0.35 | 225.3 | 230.5 | 145.7 | 149.3 |
| **Mean ± SEM** | | 0.30  ±  0.019 | 0.38  ±  0.022 | 0.39  ±  0.023 | 0.44  ±  0.016 | 0.34  ±  0.013 | 189.38  ±  22.90 | 191.22  ±  22.62 | 117.22  ±  13.06 | 122.22  ±  13.12 |

Averages values of raw data were expressed as a Mean ± SEM, n=5, For numerical results, one-way analyses of variance (ANOVA) with Tukey-Kramer Multiple Comparisons posttests were performed using GraphPad InStat Version 3 (GraphPad Software). The minimum value of *p*<0.05 was considered as significant. *^c^*p*<0.05*, ***^c^*p*<0.01, ***^c^*p***<**0.001 compared to Control group; **p*<0.05, ***p*<0.01, ****p*<0.001 compared to Arthritic control group.

**Effect of zerumbone (ZBN), Ellagic acid (ELA), Quercetin (QCT) and standard Diclofenac sodium (DfS) on Inhibition of Albumin Denaturation Assay**

| **Treatment** | **Inhibition of Albumin Denaturation Assay** | | | | | | | | | **Mean**  % inhibition  ±  SEM |
| --- | --- | --- | --- | --- | --- | --- | --- | --- | --- | --- |
|  | **T-1** | | | **T-2** | | | **T-3** | | |  |
|  | Concentration (μg/ml) | Abs at 660 nm | % inhibition | Concentration (μg/ml) | Abs at 660 nm | % inhibition | Concentration (μg/ml) | Abs at 660 nm | % inhibition |  |
| Control | - | 8.1 | - | - | 7.9 |  | - | 8.4 |  |  |
| **Standard Diclofenac sodium (DfS)** | 10 | 2.5 | 69.14 | 10 | 2.3 | 70.89 | 10 | 2.8 | 66.67 | 68.90 ± 1.224 |
|  | 20 | 1.9 | 76.54 | 20 | 1.6 | 79.75 | 20 | 2.1 | 75.00 | 77.10 ± 1.399 |
|  | 40 | 1.5 | 81.48 | 40 | 1.3 | 83.54 | 40 | 1.7 | 79.76 | 81.59 ± 1.093 |
|  | 80 | 0.7 | 91.36 | 80 | 0.5 | 93.67 | 80 | 0.9 | 89.29 | 91.44 ± 1.265 |
|  | 100 | 0.3 | 96.30 | 100 | 0.2 | 97.47 | 100 | 0.5 | 94.05 | 95.94 ± 1.004 |
| **Zerumbone (ZBN)** | 10 | 3.5 | 56.79 | 10 | 3.3 | 58.23 | 10 | 3.9 | 53.57 | 56.20 ± 1.378 |
|  | 20 | 2.5 | 69.14 | 20 | 2.1 | 73.42 | 20 | 2.8 | 66.67 | 69.74 ± 1.972 |
|  | 40 | 1.6 | 80.25 | 40 | 1.4 | 82.28 | 40 | 1.9 | 77.38 | 79.97 ± 1.421 |
|  | 80 | 1.0 | 87.65 | 80 | 0.8 | 89.87 | 80 | 1.4 | 83.33 | 86.95 ± 1.920 |
|  | 100 | 0.9 | 88.88 | 100 | 0.5 | 93.67 | 100 | 1.1 | 86.90 | 89.82 ± 2.009 |
| **Ellagic acid (ELA)** | 10 | 3.9 | 51.85 | 10 | 3.5 | 55.69 | 10 | 4.1 | 51.19 | 52.91 ± 1.403 |
|  | 20 | 2.7 | 66.66 | 20 | 2.5 | 68.35 | 20 | 3.5 | 58.33 | 64.45 ± 3.098 |
|  | 40 | 2.6 | 67.90 | 40 | 2.1 | 73.42 | 40 | 2.7 | 67.85 | 69.72 ± 1.848 |
|  | 80 | 1.1 | 86.41 | 80 | 0.9 | 88.61 | 80 | 1.7 | 79.76 | 84.93 ± 2.660 |
|  | 100 | 0.8 | 90.12 | 100 | 0.5 | 93.67 | 100 | 1.1 | 86.90 | 90.23 ± 1.955 |
| **Quercetin (QCT)** | 10 | 2.9 | 64.20 | 10 | 2.7 | 65.82 | 10 | 3.3 | 60.71 | 63.58 ± 1.508 |
|  | 20 | 2.1 | 74.10 | 20 | 1.9 | 75.95 | 20 | 2.7 | 67.86 | 72.64 ± 2.447 |
|  | 40 | 1.9 | 76.54 | 40 | 1.5 | 81.01 | 40 | 2.1 | 75.00 | 77.52 ± 1.802 |
|  | 80 | 1.6 | 80.25 | 80 | 0.8 | 89.87 | 80 | 1.9 | 77.65 | 82.59 ± 3.717 |
|  | 100 | 0.7 | 91.36 | 100 | 0.4 | 94.94 | 100 | 0.9 | 89.29 | 91.86 ± 1.650 |

Averages values of raw data were expressed as a Mean ± Standard Error Mean (SEM), n=3

**Effect of zerumbone (ZBN), Ellagic acid (ELA), Quercetin (QCT) and standard Diclofenac sodium (DfS) on Heat Induced Haemolysis Assay**

| **Treatment** | **Heat Induced Haemolysis Assay** | | | | | | | | | **Mean**  % inhibition  ±  SEM |
| --- | --- | --- | --- | --- | --- | --- | --- | --- | --- | --- |
|  | **T-1** | | | **T-2** | | | **T-3** | | |  |
|  | Concentration (μg/ml) | Abs at 560 nm | % inhibition | Conc. (μg/ml) | Abs at 560 nm | % inhibition | Conc. (μg/ml) | Abs at 560 nm | % inhibition |  |
| Control | - | 0.90 | - | - | 0.80 | - | - | 0.90 | - |  |
| **Standard Diclofenac sodium (DfS)** | 10 | 0.80 | 11.11 | 10 | 0.70 | 12.50 | 10 | 0.75 | 16.67 | 13.43 ± 1.67 |
|  | 20 | 0.60 | 33.33 | 20 | 0.50 | 37.50 | 20 | 0.54 | 38.89 | 36.57 ± 1.67 |
|  | 40 | 0.22 | 77.78 | 40 | 0.30 | 62.50 | 40 | 0.29 | 67.78 | 69.35 ± 4.48 |
|  | 80 | 0.07 | 92.22 | 80 | 0.06 | 92.50 | 80 | 0.09 | 90.00 | 91.57 ± 0.79 |
|  | 100 | 0.03 | 96.67 | 100 | 0.03 | 96.25 | 100 | 0.04 | 95.56 | 96.16 ± 0.32 |
| **Zerumbone (ZBN)** | 10 | 0.60 | 33.33 | 10 | 0.60 | 25.00 | 10 | 0.65 | 27.78 | 28.70 ± 2.45 |
|  | 20 | 0.50 | 44.44 | 20 | 0.50 | 37.50 | 20 | 0.56 | 38.89 | 40.28 ± 2.12 |
|  | 40 | 0.40 | 55.56 | 40 | 0.40 | 50.00 | 40 | 0.47 | 47.78 | 51.11 ± 2.31 |
|  | 80 | 0.30 | 66.67 | 80 | 0.30 | 62.50 | 80 | 0.26 | 71.11 | 66.76 ± 2.49 |
|  | 100 | 0.20 | 77.78 | 100 | 0.20 | 75.00 | 100 | 0.17 | 81.11 | 77.96 ± 1.78 |
| **Ellagic acid (ELA)** | 10 | 0.87 | 03.33 | 10 | 0.79 | 01.25 | 10 | 0.85 | 05.56 | 03.38 ± 1.24 |
|  | 20 | 0.80 | 11.11 | 20 | 0.70 | 12.50 | 20 | 0.77 | 14.44 | 12.68 ± 0.96 |
|  | 40 | 0.70 | 22.22 | 40 | 0.60 | 25.00 | 40 | 0.67 | 25.56 | 24.26 ± 1.03 |
|  | 80 | 0.60 | 33.33 | 80 | 0.50 | 37.50 | 80 | 0.52 | 42.22 | 37.52 ± 2.58 |
|  | 100 | 0.30 | 66.67 | 100 | 0.30 | 62.50 | 100 | 0.34 | 62.22 | 63.80 ± 1.44 |
| **Quercetin (QCT)** | 10 | 0.70 | 22.22 | 10 | 0.60 | 25.00 | 10 | 0.72 | 20.00 | 22.41 ± 1.45 |
|  | 20 | 0.60 | 33.33 | 20 | 0.50 | 37.50 | 20 | 0.57 | 36.67 | 35.83 ± 1.27 |
|  | 40 | 0.20 | 77.78 | 40 | 0.20 | 75.00 | 40 | 0.26 | 71.11 | 74.59 ± 1.97 |
|  | 80 | 0.09 | 90.00 | 80 | 0.10 | 87.50 | 80 | 0.10 | 88.89 | 88.80 ± 0.72 |
|  | 100 | 0.04 | 95.56 | 100 | 0.05 | 93.75 | 100 | 0.05 | 94.44 | 94.58 ± 0.53 |

Averages values of raw data were expressed as a Mean ± Standard Error Mean (SEM), n=3

**Effect of zerumbone (ZBN), Ellagic acid (ELA), Quercetin (QCT) and standard Diclofenac sodium (DfS) on Proteinase inhibition assay**

| **Treatment** | **Protease Inhibition Assay** | | | | | | | | | **Mean**  % inhibition  ±  SEM |
| --- | --- | --- | --- | --- | --- | --- | --- | --- | --- | --- |
|  | **T-1** | | | **T-2** | | | **T-3** | | |  |
|  | Concentration (μg/ml) | Abs at 280 nm | % inhibition | Conc. (μg/ml) | Abs at 280 nm | % inhibition | Conc. (μg/ml) | Abs at 280 nm | % inhibition |  |
| Control |  | 8.3 |  |  | 7.5 |  | - | 7.9 | - |  |
| **Standard Diclofenac sodium (DfS)** | 10 | 2.9 | 65.06 | 10 | 2.7 | 64.00 | 10 | 2.8 | 64.56 | 64.54 ± 0.31 |
|  | 20 | 2.3 | 72.29 | 20 | 2.0 | 73.33 | 20 | 2.1 | 73.42 | 73.01 ± 0.36 |
|  | 40 | 1.5 | 81.93 | 40 | 1.2 | 84.00 | 40 | 1.3 | 83.54 | 83.16 ± 0.63 |
|  | 80 | 0.9 | 89.16 | 80 | 0.8 | 89.33 | 80 | 0.8 | 89.87 | 89.45 ± 0.21 |
|  | 100 | 0.6 | 92.77 | 100 | 0.4 | 94.67 | 100 | 0.5 | 93.67 | 93.70 ± 0.55 |
| **Zerumbone (ZBN)** | 10 | 2.1 | 74.69 | 10 | 2.0 | 73.33 | 10 | 2.2 | 72.15 | 73.39 ± 0.73 |
|  | 20 | 1.2 | 85.54 | 20 | 1.2 | 84.00 | 20 | 1.4 | 82.28 | 83.94 ± 0.94 |
|  | 40 | 0.7 | 91.57 | 40 | 0.6 | 92.00 | 40 | 0.8 | 89.87 | 91.15 ± 0.65 |
|  | 80 | 0.2 | 97.59 | 80 | 0.3 | 96.00 | 80 | 0.3 | 96.20 | 96.59 ± 0.50 |
|  | 100 | 0.02 | 99.76 | 100 | 0.03 | 99.60 | 100 | 0.05 | 99.37 | 99.58 ± 0.11 |
| **Ellagic acid (ELA)** | 10 | 2.7 | 67.47 | 10 | 2.5 | 66.67 | 10 | 2.6 | 67.09 | 67.10 ± 0.23 |
|  | 20 | 1.5 | 81.93 | 20 | 1.3 | 82.67 | 20 | 1.5 | 81.01 | 81.87 ± 0.48 |
|  | 40 | 0.9 | 89.16 | 40 | 0.7 | 90.67 | 40 | 0.8 | 89.87 | 89.90 ± 0.44 |
|  | 80 | 0.5 | 93.98 | 80 | 0.3 | 96.00 | 80 | 0.3 | 96.20 | 95.39 ± 0.71 |
|  | 100 | 0.3 | 96.39 | 100 | 0.1 | 98.67 | 100 | 0.2 | 97.47 | 97.51 ± 0.66 |
| **Quercetin (QCT)** | 10 | 1.9 | 77.11 | 10 | 1.7 | 77.33 | 10 | 1.8 | 77.22 | 77.22 ± 0.06 |
|  | 20 | 1.2 | 85.54 | 20 | 1.0 | 86.67 | 20 | 1.2 | 84.81 | 85.67 ± 0.54 |
|  | 40 | 0.7 | 91.57 | 40 | 0.5 | 93.33 | 40 | 0.6 | 92.41 | 92.44 ± 0.51 |
|  | 80 | 0.4 | 95.18 | 80 | 0.2 | 97.33 | 80 | 0.3 | 96.20 | 96.24 ± 0.62 |
|  | 100 | 0.09 | 98.91 | 100 | 0.1 | 98.67 | 100 | 0.07 | 99.11 | 98.89 ± 0.13 |

Averages values of raw data were expressed as a Mean ± Standard Error Mean (SEM), n=3

**In-vivo anti-arthritic activity (serum and joint tissue extract TNF-α level at 450 nm)**

| **Sl no** | **Samples & Dose** | **Absorbance at 450 nm** | | **Mean** | **Concentration**  **in pg/mL** |
| --- | --- | --- | --- | --- | --- |
|  |  | **T1** | **T2** |  |  |
|  | Blank | 0.0823 | 0.0930 | 0.08765 | 000 |
|  | Standard 500 | 1.6118 | 1.5992 | 1.60550 | 500 |
|  | Standard 250 | 0.8702 | 0.9238 | 0.89700 | 250 |
|  | Standard 125 | 0.5186 | 0.5114 | 0.51500 | 125 |
|  | Standard 62.5 | 0.3478 | 0.3492 | 0.34850 | 62.5 |
|  | Standard 31.2 | 0.2277 | 0.2391 | 0.23340 | 31.2 |
|  | **Trend line Equation**  **Y – 0.134**  **X = -------------**  **0.003** | | | | |

Averages values of raw data were expressed as a Mean ± SEM, n=5, For numerical results, one-way analyses of variance (ANOVA) with Tukey-Kramer Multiple Comparisons posttests were performed using GraphPad InStat Version 3 (GraphPad Software). The minimum value of p<0.05 was considered as significant. *^c^p<0.05, **^c^p<0.01, ***^c^p<0.001 compared to Control group; *p<0.05, **p<0.01, ***p<0.001 compared to Arthritic control group.

**Test samples (Serum) TNF-α level at 450 nm**

| **Sl no** | **Samples & Dose** | **Absorbance at 450 nm** | | **Mean** | **Concentration**  **in pg/mL** | **Concentration in pg/mL**  **±**  **SEM** |
| --- | --- | --- | --- | --- | --- | --- |
|  |  | **T1** | **T2** |  |  |  |
|  | **Test samples (Serum)** |  |  | **“Y”** | **“X”** |  |
|  | Normal control group 1 | 0.2535 | 0.2253 | 0.2394 | 035.133 | 51.957  ±  14.508 |
|  | Normal control group 2 | 0.3679 | 0.3363 | 0.3521 | 072.700 |  |
|  | Normal control group 3 | 0.4233 | 0.4363 | 0.4298 | 098.600 |  |
|  | Normal control group 4 | 0.1936 | 0.2069 | 0.2003 | 022.083 |  |
|  | Normal control group 5 | 0.2393 | 0.2163 | 0.2278 | 031.267 |  |
|  | Arthritic Control group 1 | 1.3023 | 1.3344 | 1.3184 | 394.783 | 455.923  ±  16.009  C*** |
|  | Arthritic Control group 2 | 1.5363 | 1.6339 | 1.5851 | 483.700 |  |
|  | Arthritic Control group 3 | 1.3693 | 1.7393 | 1.5543 | 473.433 |  |
|  | Arthritic Control group 4 | 1.5633 | 1.4303 | 1.4968 | 454.267 |  |
|  | Arthritic Control group 5 | 1.3663 | 1.6003 | 1.5543 | 473.430 |  |
|  | Diclofenac Na treated Group 1 | 0.9873 | 1.0355 | 1.0114 | 292.467 | 258.337  ±  16.661  *** |
|  | Diclofenac Na treated Group 2 | 0.8893 | 1.0336 | 0.9615 | 275.817 |  |
|  | Diclofenac Na treated Group 3 | 0.7393 | 0.7036 | 0.7215 | 195.817 |  |
|  | Diclofenac Na treated Group 4 | 1.0663 | 0.7393 | 0.9028 | 256.267 |  |
|  | Diclofenac Na treated Group 5 | 0.9636 | 0.9323 | 0.9480 | 271.317 |  |
|  | Ellagic acid treated Group 1 | 0.2564 | 0.2761 | 0.2663 | 044.083 | 53.853  ±  4.884  *** |
|  | Ellagic acid treated Group 2 | 0.2639 | 0.2439 | 0.2539 | 039.967 |  |
|  | Ellagic acid treated Group 3 | 0.3363 | 0.3093 | 0.3228 | 062.933 |  |
|  | Ellagic acid treated Group 4 | 0.3069 | 0.3296 | 0.3183 | 061.417 |  |
|  | Ellagic acid treated Group 5 | 0.2933 | 0.3399 | 0.3166 | 060.867 |  |
|  | Querceitin treated Group 1 | 1.0962 | 1.0834 | 1.0898 | 318.600 | 345.963  ±  13.226  *** |
|  | Querceitin treated Group 2 | 1.3463 | 1.1436 | 1.2450 | 370.317 |  |
|  | Querceitin treated Group 3 | 1.2263 | 1.0639 | 1.1451 | 337.033 |  |
|  | Querceitin treated Group 4 | 1.4333 | 1.1363 | 1.2848 | 383.600 |  |
|  | Querceitin treated Group 5 | 1.2963 | 0.8933 | 1.0948 | 320.267 |  |
|  | Zerumbone Treated Group 1 | 0.3057 | 0.3150 | 0.3104 | 058.783 | 59.82  ±  7.931  *** |
|  | Zerumbone Treated Group 2 | 0.4339 | 0.3635 | 0.3987 | 088.233 |  |
|  | Zerumbone Treated Group 3 | 0.2693 | 0.2336 | 0.2515 | 039.150 |  |
|  | Zerumbone Treated Group 4 | 0.2953 | 0.3136 | 0.3045 | 56.8167 |  |
|  | Zerumbone Treated Group 5 | 0.3057 | 0.2993 | 0.3025 | 56.1667 |  |

**Test samples (Joint Tissue extract) TNF-α level at 450 nm**

| **Sl no** | **Samples & Dose** | **Absorbance at 450 nm** | | **Mean** | **Concentration**  **in pg/mL** | **Concentration in pg/mL**  **±**  **SEM** |
| --- | --- | --- | --- | --- | --- | --- |
|  |  | **T1** | **T2** |  |  |  |
|  | **Test samples (Joint Tissue extract)** |  |  | **“Y”** | **“X”** |  |
|  | Normal control group 1 | 0.2058 | 0.2652 | 0.2355 | 033.833 | 31.513  ±  5.138 |
|  | Normal control group 2 | 0.2039 | 0.1953 | 0.1996 | 021.867 |  |
|  | Normal control group 3 | 0.1869 | 0.1999 | 0.1934 | 019.800 |  |
|  | Normal control group 4 | 0.2953 | 0.2636 | 0.2795 | 048.483 |  |
|  | Normal control group 5 | 0.2359 | 0.2336 | 0.2348 | 033.583 |  |
|  | Arthritic Control group 1 | 1.0544 | 1.375 | 1.2147 | 360.233 | 341.287  ±  15.133  C*** |
|  | Arthritic Control group 2 | 1.3639 | 1.1336 | 1.2488 | 371.583 |  |
|  | Arthritic Control group 3 | 1.1366 | 0.8993 | 1.0180 | 294.650 |  |
|  | Arthritic Control group 4 | 1.0293 | 1.1363 | 1.0828 | 316.267 |  |
|  | Arthritic Control group 5 | 1.3963 | 1.0539 | 1.2251 | 363.700 |  |
|  | Diclofenac Na treated Group 1 | 0.2830 | 0.2261 | 0.2546 | 040.183 | 51.913  ±  7.442  *** |
|  | Diclofenac Na treated Group 2 | 0.2656 | 0.2336 | 0.2496 | 038.533 |  |
|  | Diclofenac Na treated Group 3 | 0.3396 | 0.3645 | 0.3521 | 072.683 |  |
|  | Diclofenac Na treated Group 4 | 0.3623 | 0.3096 | 0.3360 | 067.317 |  |
|  | Diclofenac Na treated Group 5 | 0.2395 | 0.2736 | 0.2566 | 040.850 |  |
|  | Ellagic acid treated Group 1 | 0.4221 | 0.4609 | 0.4415 | 102.500 | 96.437  ±  6.936  *** |
|  | Ellagic acid treated Group 2 | 0.3996 | 0.3793 | 0.3895 | 085.150 |  |
|  | Ellagic acid treated Group 3 | 0.5009 | 0.4839 | 0.4924 | 119.467 |  |
|  | Ellagic acid treated Group 4 | 0.3693 | 0.3796 | 0.3745 | 080.150 |  |
|  | Ellagic acid treated Group 5 | 0.4039 | 0.4336 | 0.4188 | 094.917 |  |
|  | Querceitin treated Group 1 | 0.7653 | 0.6954 | 0.7304 | 198.783 | 213.247  ±  8.481  *** |
|  | Querceitin treated Group 2 | 0.7937 | 0.8339 | 0.8138 | 226.600 |  |
|  | Querceitin treated Group 3 | 0.7693 | 0.7365 | 0.7529 | 206.300 |  |
|  | Querceitin treated Group 4 | 0.7356 | 0.7039 | 0.7198 | 195.250 |  |
|  | Querceitin treated Group 5 | 0.8435 | 0.8603 | 0.8519 | 239.300 |  |
|  | Zerumbone Treated Group 1 | 0.3975 | 0.4035 | 0.4005 | 088.833 | 105.510  ±  7.709  *** |
|  | Zerumbone Treated Group 2 | 0.4639 | 0.4963 | 0.4801 | 115.367 |  |
|  | Zerumbone Treated Group 3 | 0.3796 | 0.3969 | 0.3883 | 084.750 |  |
|  | Zerumbone Treated Group 4 | 0.5096 | 0.4803 | 0.4950 | 120.317 |  |
|  | Zerumbone Treated Group 5 | 0.4738 | 0.5039 | 0.4889 | 118.283 |  |

**In-vivo anti-arthritic activity (serum and joint tissue extract IL-10 level at 450 nm)**

| **Sl No** | **Samples & Dose** | **Absorbance at 450 nm** | | **Mean** | **Concentration**  **In pg/mL** |
| --- | --- | --- | --- | --- | --- |
|  |  | **T1** | **T2** |  |  |
|  | Blank | 0.0659 | 0.0486 | 0.05725 | 0000 |
|  | Standard 1000 | 1.0979 | 1.0999 | 1.09890 | 1000 |
|  | Standard 500 | 0.5949 | 0.5528 | 0.57385 | 0500 |
|  | Standard 250 | 0.3368 | 0.3136 | 0.32520 | 0250 |
|  | Standard 125 | 0.2010 | 0.2000 | 0.20050 | 0125 |
|  | Standard 62.5 | 0.1459 | 0.1255 | 0.13570 | 062.5 |
|  | Standard 31.2 | 0.0921 | 0.0746 | 0.08335 | 031.2 |
|  | **Trend line Equation**  **Y – 0.062**  **X = -----------------------**  **0.001** | | | | |

Averages values of raw data were expressed as a Mean ± SEM, n=5, For numerical results, one-way analyses of variance (ANOVA) with Tukey-Kramer Multiple Comparisons posttests were performed using GraphPad InStat Version 3 (GraphPad Software). The minimum value of p<0.05 was considered as significant. *^c^p<0.05, **^c^p<0.01, ***^c^p<0.001 compared to Control group; *p<0.05, **p<0.01, ***p<0.001 compared to Arthritic control group.

**Test samples (Serum) IL-10 level at 450 nm**

| **Sl no** | **Samples & Dose** | **Absorbance at 450 nm** | | **Mean** | **Concentration**  **in pg/mL** | **Concentration in pg/mL**  **±**  **SEM** |
| --- | --- | --- | --- | --- | --- | --- |
|  |  | **T1** | **T2** |  |  |  |
|  | **Test samples (Serum)** |  |  | **“Y”** | **“X”** |  |
|  | Normal control group 1 | 0.3795 | 0.4097 | 0.3946 | 332.60 | 321.06  ±  18.180 |
|  | Normal control group 2 | 0.3497 | 0.3639 | 0.3568 | 294.80 |  |
|  | Normal control group 3 | 0.4239 | 0.4336 | 0.4288 | 366.75 |  |
|  | Normal control group 4 | 0.3945 | 0.4209 | 0.4077 | 345.70 |  |
|  | Normal control group 5 | 0.3053 | 0.3496 | 0.3275 | 265.45 |  |
|  | Arthritic Control group 1 | 0.0935 | 0.0975 | 0.0955 | 033.50 | 39.83  ±  05.056  C*** |
|  | Arthritic Control group 2 | 0.1293 | 0.1056 | 0.1175 | 055.45 |  |
|  | Arthritic Control group 3 | 0.0963 | 0.0939 | 0.0951 | 033.10 |  |
|  | Arthritic Control group 4 | 0.1037 | 0.1163 | 0.1100 | 048.00 |  |
|  | Arthritic Control group 5 | 0.0896 | 0.0926 | 0.0911 | 029.10 |  |
|  | Diclofenac Na treated Group 1 | 0.1735 | 0.1957 | 0.1846 | 122.60 | 134.38  ±  12.793  *** |
|  | Diclofenac Na treated Group 2 | 0.2036 | 0.2375 | 0.2206 | 158.55 |  |
|  | Diclofenac Na treated Group 3 | 0.1639 | 0.1993 | 0.1816 | 119.60 |  |
|  | Diclofenac Na treated Group 4 | 0.1536 | 0.1733 | 0.1635 | 101.45 |  |
|  | Diclofenac Na treated Group 5 | 0.2439 | 0.2195 | 0.2317 | 169.70 |  |
|  | Ellagic acid treated Group 1 | 0.2063 | 0.1643 | 0.1853 | 123.30 | 123.90  ±  05.003  *** |
|  | Ellagic acid treated Group 2 | 0.1833 | 0.2152 | 0.1993 | 137.25 |  |
|  | Ellagic acid treated Group 3 | 0.1952 | 0.1736 | 0.1844 | 122.40 |  |
|  | Ellagic acid treated Group 4 | 0.1656 | 0.1723 | 0.1690 | 106.95 |  |
|  | Ellagic acid treated Group 5 | 0.1739 | 0.2093 | 0.1916 | 129.60 |  |
|  | Querceitin treated Group 1 | 0.1537 | 0.1895 | 0.1716 | 109.60 | 113.29  ±  06.341  *** |
|  | Querceitin treated Group 2 | 0.1859 | 0.2093 | 0.1976 | 135.60 |  |
|  | Querceitin treated Group 3 | 0.1796 | 0.1459 | 0.1628 | 100.75 |  |
|  | Querceitin treated Group 4 | 0.1965 | 0.1633 | 0.1799 | 117.90 |  |
|  | Querceitin treated Group 5 | 0.1556 | 0.1736 | 0.1646 | 102.60 |  |
|  | Zerumbone Treated Group 1 | 0.1319 | 0.1755 | 0.1537 | 091.70 | 100.87  ±  08.543  ** |
|  | Zerumbone Treated Group 2 | 0.2029 | 0.1796 | 0.1913 | 129.25 |  |
|  | Zerumbone Treated Group 3 | 0.1396 | 0.1525 | 0.1461 | 084.05 |  |
|  | Zerumbone Treated Group 4 | 0.1869 | 0.1603 | 0.1736 | 111.60 |  |
|  | Zerumbone Treated Group 5 | 0.1532 | 0.1463 | 0.1498 | 087.75 |  |

**Test samples (Joint Tissue extract) IL-10 level at 450 nm**

| **Sl no** | **Samples & Dose** | **Absorbance at 450 nm** | | **Mean** | **Concentration**  **in pg/mL** | **Concentration in pg/mL**  **±**  **SEM** |
| --- | --- | --- | --- | --- | --- | --- |
|  |  | **T1** | **T2** |  |  |  |
|  | **Test samples (Joint Tissue extract)** |  |  | **“Y”** | **“X”** |  |
|  | Normal control group 1 | 0.5344 | 0.5266 | 0.5305 | 468.50 | 460.19  ±  12.723 |
|  | Normal control group 2 | 0.4895 | 0.4732 | 0.4814 | 419.35 |  |
|  | Normal control group 3 | 0.5719 | 0.5332 | 0.5526 | 490.55 |  |
|  | Normal control group 4 | 0.5582 | 0.5223 | 0.5403 | 478.25 |  |
|  | Normal control group 5 | 0.5133 | 0.4993 | 0.5063 | 444.30 |  |
|  | Arthritic Control group 1 | 0.1431 | 0.1636 | 0.1534 | 091.35 | 89.03  ±  11.675  C*** |
|  | Arthritic Control group 2 | 0.1036 | 0.1405 | 0.1221 | 060.05 |  |
|  | Arthritic Control group 3 | 0.1553 | 0.1266 | 0.1410 | 078.95 |  |
|  | Arthritic Control group 4 | 0.2035 | 0.1823 | 0.1929 | 130.90 |  |
|  | Arthritic Control group 5 | 0.1525 | 0.1393 | 0.1459 | 083.90 |  |
|  | Diclofenac Na treated Group 1 | 0.3795 | 0.3575 | 0.3685 | 306.50 | 334.11  ±  15.385  *** |
|  | Diclofenac Na treated Group 2 | 0.4432 | 0.4262 | 0.4347 | 372.70 |  |
|  | Diclofenac Na treated Group 3 | 0.3858 | 0.4058 | 0.3958 | 333.80 |  |
|  | Diclofenac Na treated Group 4 | 0.3696 | 0.3423 | 0.3560 | 293.95 |  |
|  | Diclofenac Na treated Group 5 | 0.4436 | 0.4076 | 0.4256 | 363.60 |  |
|  | Ellagic acid treated Group 1 | 0.4002 | 0.3897 | 0.3950 | 332.95 | 332.52  ±  11.812  *** |
|  | Ellagic acid treated Group 2 | 0.3563 | 0.3745 | 0.3654 | 303.40 |  |
|  | Ellagic acid treated Group 3 | 0.4493 | 0.3939 | 0.4216 | 359.60 |  |
|  | Ellagic acid treated Group 4 | 0.4132 | 0.4266 | 0.4199 | 357.90 |  |
|  | Ellagic acid treated Group 5 | 0.3892 | 0.3523 | 0.3710 | 308.75 |  |
|  | Querceitin treated Group 1 | 0.2561 | 0.1985 | 0.2273 | 165.30 | 147.29  ±  14.253  * |
|  | Querceitin treated Group 2 | 0.1543 | 0.1739 | 0.1641 | 102.10 |  |
|  | Querceitin treated Group 3 | 0.2379 | 0.2532 | 0.2456 | 183.55 |  |
|  | Querceitin treated Group 4 | 0.2023 | 0.2333 | 0.2178 | 155.80 |  |
|  | Querceitin treated Group 5 | 0.1735 | 0.2099 | 0.1917 | 129.70 |  |
|  | Zerumbone Treated Group 1 | 0.3181 | 0.2825 | 0.3003 | 238.30 | 226.56  ±  09.933  *** |
|  | Zerumbone Treated Group 2 | 0.2535 | 0.2733 | 0.2634 | 201.40 |  |
|  | Zerumbone Treated Group 3 | 0.2832 | 0.2499 | 0.2666 | 204.55 |  |
|  | Zerumbone Treated Group 4 | 0.3037 | 0.3223 | 0.3130 | 251.00 |  |
|  | Zerumbone Treated Group 5 | 0.2935 | 0.3056 | 0.2996 | 237.55 |  |

**In-vivo anti-arthritic activity (serum and joint tissue extract IL-1β level at 450 nm and 550nm)**

| **Sl No** | **Samples & Dose** | **Absorbance at 450 nm** | | **Mean at 450** | **Absorbance at 550 nm** | | **Mean at 550** | **Absorbance**  450nm - 550nm | **Concentration**  **In pg/mL** |
| --- | --- | --- | --- | --- | --- | --- | --- | --- | --- |
|  |  | **T1** | **T2** |  | **T1** | **T2** |  |  |  |
|  | Blank | 0.0946 | 0.0902 | 0.0924 | 0.0613 | 0.0550 | 0.0584 | 0.0340 | 0000 |
|  | Standard 2500 | 0.9929 | 1.0547 | 1.0238 | 0.0473 | 0.0521 | 0.0497 | 0.9741 | 2500 |
|  | Standard 1000 | 0.4753 | 0.5221 | 0.4987 | 0.0477 | 0.0483 | 0.0480 | 0.4507 | 1000 |
|  | Standard 400 | 0.2163 | 0.2927 | 0.2545 | 0.0408 | 0.0478 | 0.0440 | 0.2105 | 0400 |
|  | Standard 160 | 0.1584 | 0.1458 | 0.1521 | 0.0449 | 0.0474 | 0.0462 | 0.1059 | 0160 |
|  | Standard 64 | 0.1297 | 0.1321 | 0.1309 | 0.0435 | 0.0512 | 0.0474 | 0.0838 | 0064 |
|  | Standard 25.6 | 0.1020 | 0.1174 | 0.1097 | 0.0519 | 0.0531 | 0.0525 | 0.0572 | 025.6 |

**Y – 0.0525**

**X = -----------------------**

**0.0003**

Averages values of raw data were expressed as a Mean ± SEM, n=5, For numerical results, one-way analyses of variance (ANOVA) with Tukey-Kramer Multiple Comparisons posttests were performed using GraphPad InStat Version 3 (GraphPad Software). The minimum value of p<0.05 was considered as significant. *^c^p<0.05, **^c^p<0.01, ***^c^p<0.001 compared to Control group; *p<0.05, **p<0.01, ***p<0.001 compared to Arthritic control group.

**In-vivo anti-arthritic activity (serum IL-1β level at 450 nm and 550nm)**

| **Sl No** | **Samples & Dose** | **Absorbance at 450 nm** | | **Mean at 450** | **Absorbance at 550 nm** | | **Mean at 550** | **Absorbance**  450nm - 550nm | **Concentration**  **In pg/mL** | **Concentration in pg/mL**  **±**  **SEM** |
| --- | --- | --- | --- | --- | --- | --- | --- | --- | --- | --- |
|  |  | **T1** | **T2** |  | **T1** | **T2** |  |  |  |  |
|  | **Test samples (Serum)** |  |  |  |  |  |  | **“Y”** | **“X”** |  |
|  | Normal control group 1 | 0.1004 | 0.1506 | 0.1255 | 0.0556 | 0.0534 | 0.0545 | 0.0710 | 061.67 | 68.44  ±  6.667 |
|  | Normal control group 2 | 0.1252 | 0.1435 | 0.1344 | 0.0596 | 0.0573 | 0.0585 | 0.0759 | 078.00 |  |
|  | Normal control group 3 | 0.1434 | 0.1239 | 0.1337 | 0.0531 | 0.0553 | 0.0542 | 0.0795 | 089.83 |  |
|  | Normal control group 4 | 0.1054 | 0.1336 | 0.1195 | 0.0495 | 0.0503 | 0.0499 | 0.0696 | 057.00 |  |
|  | Normal control group 5 | 0.1426 | 0.1093 | 0.1260 | 0.0586 | 0.0549 | 0.0568 | 0.0692 | 055.67 |  |
|  | Arthritic Control group 1 | 0.1949 | 0.2057 | 0.2003 | 0.0543 | 0.0483 | 0.0513 | 0.1490 | 321.67 | 332.30  ±  16.188  C*** |
|  | Arthritic Control group 2 | 0.2356 | 0.2035 | 0.2196 | 0.0653 | 0.0593 | 0.0623 | 0.1573 | 349.17 |  |
|  | Arthritic Control group 3 | 0.1936 | 0.1739 | 0.1838 | 0.0476 | 0.0436 | 0.0456 | 0.1382 | 285.50 |  |
|  | Arthritic Control group 4 | 0.2039 | 0.2356 | 0.2198 | 0.0539 | 0.0509 | 0.0524 | 0.1674 | 382.83 |  |
|  | Arthritic Control group 5 | 0.1869 | 0.2139 | 0.2004 | 0.0485 | 0.0539 | 0.0512 | 0.1492 | 322.33 |  |
|  | Diclofenac Na Group 1 | 0.1708 | 0.1697 | 0.1703 | 0.0503 | 0.0493 | 0.0498 | 0.1205 | 226.50 | 211.84  ±  11.326  *** |
|  | Diclofenac Na Group 2 | 0.1557 | 0.1887 | 0.1722 | 0.0476 | 0.0453 | 0.0465 | 0.1258 | 244.17 |  |
|  | Diclofenac Na Group 3 | 0.1846 | 0.1534 | 0.1690 | 0.0556 | 0.0573 | 0.0565 | 0.1126 | 200.17 |  |
|  | Diclofenac Na Group 4 | 0.1453 | 0.1732 | 0.1593 | 0.0523 | 0.0546 | 0.0535 | 0.1058 | 177.67 |  |
|  | Diclofenac Na Group 5 | 0.1732 | 0.1563 | 0.1648 | 0.0489 | 0.0492 | 0.0491 | 0.1157 | 210.67 |  |
|  | Ellagic acid treated Group 1 | 0.1315 | 0.1091 | 0.1203 | 0.0484 | 0.0504 | 0.0494 | 0.0709 | 061.33 | 67.63  ±  5.116  *** |
|  | Ellagic acid treated Group 2 | 0.1332 | 0.1203 | 0.1268 | 0.0532 | 0.0564 | 0.0548 | 0.0720 | 064.83 |  |
|  | Ellagic acid treated Group 3 | 0.1156 | 0.1233 | 0.1195 | 0.0453 | 0.0432 | 0.0443 | 0.0752 | 075.67 |  |
|  | Ellagic acid treated Group 4 | 0.1135 | 0.1439 | 0.1287 | 0.0506 | 0.0523 | 0.0515 | 0.0773 | 082.50 |  |
|  | Ellagic acid treated Group 5 | 0.1203 | 0.1139 | 0.1171 | 0.0476 | 0.0493 | 0.0485 | 0.0687 | 053.83 |  |
|  | Querceitin treated Group 1 | 0.1717 | 0.1533 | 0.1625 | 0.0557 | 0.0515 | 0.0536 | 0.1089 | 188.00 | 182.94  ±  8.079  *** |
|  | Querceitin treated Group 2 | 0.1635 | 0.1529 | 0.1582 | 0.0506 | 0.0519 | 0.0513 | 0.1070 | 181.50 |  |
|  | Querceitin treated Group 3 | 0.1429 | 0.1796 | 0.1613 | 0.0476 | 0.0493 | 0.0485 | 0.1128 | 201.00 |  |
|  | Querceitin treated Group 4 | 0.1356 | 0.1703 | 0.1530 | 0.0534 | 0.0556 | 0.0545 | 0.0985 | 153.17 |  |
|  | Querceitin treated Group 5 | 0.1552 | 0.1669 | 0.1611 | 0.0516 | 0.0509 | 0.0513 | 0.1098 | 191.00 |  |
|  | Zerumbone Treated Group 1 | 0.1053 | 0.1373 | 0.1213 | 0.0547 | 0.0531 | 0.0539 | 0.0674 | 049.67 | 58.84  ±  10.501  *** |
|  | Zerumbone Treated Group 2 | 0.1353 | 0.1203 | 0.1278 | 0.0523 | 0.0506 | 0.0515 | 0.0764 | 079.50 |  |
|  | Zerumbone Treated Group 3 | 0.1056 | 0.1196 | 0.1126 | 0.0479 | 0.0493 | 0.0486 | 0.0640 | 038.33 |  |
|  | Zerumbone Treated Group 4 | 0.1432 | 0.1232 | 0.1332 | 0.0532 | 0.0553 | 0.0543 | 0.0790 | 088.17 |  |
|  | Zerumbone Treated Group 5 | 0.1153 | 0.1296 | 0.1225 | 0.0579 | 0.0589 | 0.0584 | 0.0641 | 038.50 |  |

**In-vivo anti-arthritic activity (joint tissue extracts IL-1β level at 450 nm and 550nm)**

| **Sl No** | **Samples & Dose** | **Absorbance at 450 nm** | | **Mean at 450** | **Absorbance at 550 nm** | | **Mean at 550** | **Absorbance**  450nm - 550nm | **Concentration**  **In pg/mL** | **Concentration in pg/mL**  **±**  **SEM** |
| --- | --- | --- | --- | --- | --- | --- | --- | --- | --- | --- |
|  |  | **T1** | **T2** |  | **T1** | **T2** |  |  |  |  |
|  | **Sample (Joint Tissue Extract)** |  |  |  |  |  |  | **“Y”** | **“X”** |  |
|  | Normal control group 1 | 0.1009 | 0.1605 | 0.1307 | 0.0501 | 0.0489 | 0.0495 | 0.0812 | 095.67 | 89.20  ±  7.139 |
|  | Normal control group 2 | 0.1153 | 0.1392 | 0.1273 | 0.0456 | 0.0483 | 0.0470 | 0.0803 | 092.67 |  |
|  | Normal control group 3 | 0.1363 | 0.1193 | 0.1278 | 0.0559 | 0.0537 | 0.0548 | 0.0730 | 068.33 |  |
|  | Normal control group 4 | 0.1058 | 0.1453 | 0.1256 | 0.0486 | 0.0499 | 0.0493 | 0.0763 | 079.33 |  |
|  | Normal control group 5 | 0.1353 | 0.1456 | 0.1405 | 0.0563 | 0.0536 | 0.0550 | 0.0855 | 110.00 |  |
|  | Arthritic Control group 1 | 0.2346 | 0.2064 | 0.2205 | 0.0473 | 0.0493 | 0.0483 | 0.1722 | 399.00 | 386.80  ±  9.729  C*** |
|  | Arthritic Control group 2 | 0.2192 | 0.2363 | 0.2278 | 0.0523 | 0.0509 | 0.0516 | 0.1762 | 412.17 |  |
|  | Arthritic Control group 3 | 0.1936 | 0.2369 | 0.2153 | 0.0459 | 0.0463 | 0.0461 | 0.1692 | 388.83 |  |
|  | Arthritic Control group 4 | 0.2153 | 0.2006 | 0.2080 | 0.0489 | 0.0493 | 0.0491 | 0.1589 | 354.50 |  |
|  | Arthritic Control group 5 | 0.1953 | 0.2239 | 0.2096 | 0.0436 | 0.0429 | 0.0433 | 0.1664 | 379.50 |  |
|  | Diclofenac Na Group 1 | 0.1758 | 0.1654 | 0.1706 | 0.0516 | 0.0486 | 0.0501 | 0.1205 | 226.67 | 221.40  ±  5.195  *** |
|  | Diclofenac Na Group 2 | 0.1956 | 0.1623 | 0.1790 | 0.0582 | 0.0573 | 0.0578 | 0.1212 | 229.00 |  |
|  | Diclofenac Na Group 3 | 0.1636 | 0.1532 | 0.1584 | 0.0457 | 0.0439 | 0.0448 | 0.1136 | 203.67 |  |
|  | Diclofenac Na Group 4 | 0.1737 | 0.1693 | 0.1715 | 0.0553 | 0.0532 | 0.0543 | 0.1173 | 215.83 |  |
|  | Diclofenac Na Group 5 | 0.1756 | 0.1679 | 0.1718 | 0.0505 | 0.0489 | 0.0497 | 0.1221 | 231.83 |  |
|  | Ellagic acid treated Group 1 | 0.1453 | 0.1157 | 0.1305 | 0.0507 | 0.0487 | 0.0497 | 0.0808 | 094.33 | 95.50  ±  4.985  *** |
|  | Ellagic acid treated Group 2 | 0.1387 | 0.1292 | 0.1340 | 0.0539 | 0.0506 | 0.0523 | 0.0817 | 097.33 |  |
|  | Ellagic acid treated Group 3 | 0.1565 | 0.1031 | 0.1298 | 0.0476 | 0.0493 | 0.0485 | 0.0814 | 096.17 |  |
|  | Ellagic acid treated Group 4 | 0.1163 | 0.1396 | 0.1280 | 0.0503 | 0.0531 | 0.0517 | 0.0763 | 079.17 |  |
|  | Ellagic acid treated Group 5 | 0.1256 | 0.1433 | 0.1345 | 0.0473 | 0.0503 | 0.0488 | 0.0857 | 110.50 |  |
|  | Querceitin treated Group 1 | 0.1865 | 0.1654 | 0.1760 | 0.0497 | 0.0507 | 0.0502 | 0.1258 | 244.17 | 245.67  ±  10.085  *** |
|  | Querceitin treated Group 2 | 0.1832 | 0.1687 | 0.1760 | 0.0424 | 0.0453 | 0.0439 | 0.1321 | 265.33 |  |
|  | Querceitin treated Group 3 | 0.1769 | 0.1656 | 0.1713 | 0.0556 | 0.0532 | 0.0544 | 0.1169 | 214.50 |  |
|  | Querceitin treated Group 4 | 0.1787 | 0.1899 | 0.1843 | 0.0523 | 0.0497 | 0.0510 | 0.1333 | 269.33 |  |
|  | Querceitin treated Group 5 | 0.1643 | 0.1732 | 0.1688 | 0.0462 | 0.0453 | 0.0458 | 0.1230 | 235.00 |  |
|  | Zerumbone Treated Group 1 | 0.1405 | 0.1235 | 0.1320 | 0.0474 | 0.0454 | 0.0464 | 0.0856 | 110.33 | 114.00  ±  4.410  *** |
|  | Zerumbone Treated Group 2 | 0.1235 | 0.1532 | 0.1384 | 0.0523 | 0.0536 | 0.0530 | 0.0854 | 109.67 |  |
|  | Zerumbone Treated Group 3 | 0.1332 | 0.1463 | 0.1398 | 0.0498 | 0.0476 | 0.0487 | 0.0911 | 128.50 |  |
|  | Zerumbone Treated Group 4 | 0.1439 | 0.1352 | 0.1396 | 0.0503 | 0.0526 | 0.0515 | 0.0881 | 118.67 |  |
|  | Zerumbone Treated Group 5 | 0.1193 | 0.1403 | 0.1298 | 0.0476 | 0.0453 | 0.0465 | 0.0834 | 102.83 |  |
